# Neural precursor cells tune striatal connectivity through the release of IGFBPL1

**DOI:** 10.1038/s41467-022-35341-y

**Published:** 2022-12-08

**Authors:** Erica Butti, Stefano Cattaneo, Marco Bacigaluppi, Marco Cambiaghi, Giulia Maria Scotti, Elena Brambilla, Francesca Ruffini, Giacomo Sferruzza, Maddalena Ripamonti, Fabio Simeoni, Laura Cacciaguerra, Aurora Zanghì, Angelo Quattrini, Riccardo Fesce, Paola Panina-Bordignon, Francesca Giannese, Davide Cittaro, Tanja Kuhlmann, Patrizia D’Adamo, Maria Assunta Rocca, Stefano Taverna, Gianvito Martino

**Affiliations:** 1 Neuroimmunology Unit, Division of Neuroscience, Institute of Experimental Neurology (INSPE), IRCCS San Raffaele Hospital, Milan, Italy; 2 Vita-Salute San Raffaele University, Milan, Italy; 3 Department of Neurosciences, Biomedicine and Movement Sciences, University of Verona, Verona, Italy; 4 Omics Sciences Center, IRCCS San Raffaele Hospital, Milan, Italy; 5 Neuroimaging Research Unit, Institute of Experimental Neurology (INSPE), Division of Neuroscience, IRCCS San Raffaele Hospital, Milan, Italy; 6 Neuropathology Unit, Institute of Experimental Neurology (INSPE), Division of Neuroscience, IRCCS San Raffaele Hospital, Milan, Italy; 7 Department of Biomedical Sciences, Humanitas University, Pieve Emanuele, Milan, Italy; 8 Institute of Neuropathology, University Hospital Muster, Muster, Germany; 9 Molecular genetics of Intellectual Disability, Division of Neuroscience, IRCCS San Raffaele Hospital, Milan, Italy

**Keywords:** Neuroscience, Neural stem cells, Neurological disorders

## Abstract

The adult brain retains over life endogenous neural stem/precursor cells (eNPCs) within the subventricular zone (SVZ). Whether or not these cells exert physiological functions is still unclear. In the present work, we provide evidence that SVZ-eNPCs tune structural, electrophysiological, and behavioural aspects of striatal function via secretion of insulin-like growth factor binding protein-like 1 (IGFBPL1). In mice, selective ablation of SVZ-eNPCs or selective abrogation of IGFBPL1 determined an impairment of striatal medium spiny neuron morphology, a higher failure rate in GABAergic transmission mediated by fast-spiking interneurons, and striatum-related behavioural dysfunctions. We also found IGFBPL1 expression in the human SVZ, foetal and induced-pluripotent stem cell-derived NPCs. Finally, we found a significant correlation between SVZ damage, reduction of striatum volume, and impairment of information processing speed in neurological patients. Our results highlight the physiological role of adult SVZ-eNPCs in supporting cognitive functions by regulating striatal neuronal activity.

## Abstract (web summary)

The physiological role of endogenous neural protenitor cells of the subventricular zone in adult stage is not fully understood. Here the authors show that in mice, these cells tune neuronal activity of the striatum via insulin-like growth factor binding protein-like 1 and cognitive functions.

## Introduction

Endogenous neural stem/precursor cells (eNPCs) are present in two specific neurogenic niches of the brain, the subgranular zone (SGZ) in the dentate gyrus (DG) of the hippocampus and the subventricular zone (SVZ) adjacent to the lateral ventricles^1–3^. While during adult life SGZ-eNPCs are considered crucial in physiological processes aimed to maintain memory circuits integrity and cognitive adaptability, in both humans and rodents, less clear is the physiological role of SVZ-eNPCs^4,5^. SVZ-eNPCs account for olfactory bulb (OB) granule cell renewal in rodents. However, in humans, the integration of neurons in the OB accounts for less than 1% of the total neurons exchanged over a lifetime^6^. Indeed, there is evidence that human SVZ-eNPCs might contribute to striatal neurogenesis but only as a reactive process to tissue damage^7^. A possible explanation for these differences may lie in evolutionary changes in volume and functional performances of the different brain areas in humans compared to rodents. Another potential reason is that SVZ-eNPCs might also exert non-neurogenic functions, as recently shown in pathological conditions where such cells promote neuroprotection via the release of soluble molecules^8–13^.

SVZ-eNPCs are in close contact with blood and cerebrospinal fluid (CSF) compartments^14^. Moreover, they are located very close to and interconnected with the striatum, the main input area of the basal ganglia, which has relevance in regulating cognitive processes—particularly those involved in decision-making—that is increasingly appreciated. This regulatory activity is possible because the striatum receives massive excitatory inputs from the cortex and thalamus and contains an intricate network of GABAergic synaptic connections projected by several cell types, such as medium spiny neurons (MSNs) and local interneurons^15–17^. Among local interneurons, fast-spiking interneurons (FSI) play a key role in this process since they form perisomatic synapses with MSNs, resulting in a strong inhibitory activity^18^.

These premises prompted us to hypothesize that SVZ-eNPCs might exert a non-neurogenic physiological role on striatal areas involved in cognitive processes. We found that ablation of SVZ-eNPCs in mice caused morphological changes in striatal MSNs and functional deficits characterized by an altered GABAergic synaptic transmission specifically mediated by FSIs onto MSNs. These findings were paralleled by less efficient striatal-dependent cognitive tasks^19,20^. Such effects were induced by SVZ-eNPC-mediated expression of insulin-like growth factor-binding protein-like 1 (IGFBPL1), a protein that binds and stabilizes insulin-like growth factor 1 (IGF-1)^21,22^. In addition, we demonstrated that human foetal, adult, and induced pluripotent stem cell (iPS)-derived NPCs did indeed significantly express IGFBPL1. Finally, as a proof of principle, pathological injury of the SVZ in humans with a neurodegenerative disorder significantly correlated with striatal dysfunction and cognitive impairment affecting decision-related domains. Altogether, these data suggest a mechanism used by SVZ-eNPCs to exert essential non-neurogenic homeostatic functions in striatal areas.

## Results

### Ablation of eNPCs changes striatal medium spiny neuron morphology

**Fig. 1:**
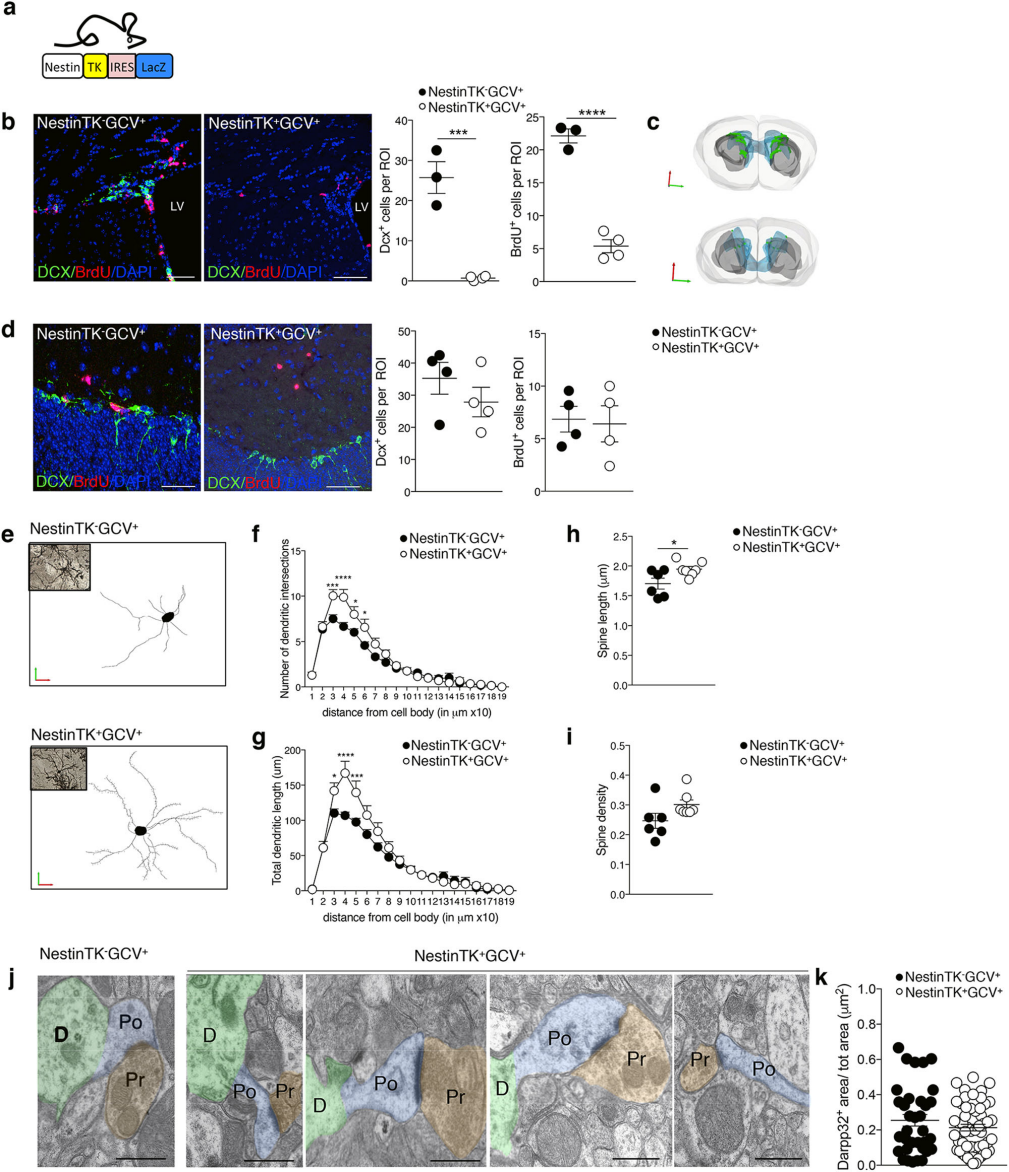
SVZ-eNPC ablation induces structural modifications of striatal MSNs. **a** Schematic representation of transgenic mouse model with structure of gene expression cassette. **b** Representative images and quantification of eNPC ablation after GCV treatment in NestinTK^-^GCV^+^ and NestinTK^+^GCV^+^ mice: neuroblasts labelled by doublecortin (DCX, in green) and transient amplifying cells for BrdU (in red). Nuclei in blue counterstained by DAPI. LV, lateral ventricle. Scale bar: 50 μm. *n* = 3 NestinTK^-^GCV^+^ and *n* = 4 NestinTK^+^GCV^+^ mice. Values represent mean ± SEM ***p = 0.0006; ****p = 8.9 × 10^−5^. Unpaired two-tailed *t*-test. **c** Representative 3D reconstructions of the forebrain staining for DCX (in green) of a representative NestinTK^-^GCV^+^ (up) and NestinTK^+^GCV^+^ (down) mouse after ablation. Brain hemispheres in light grey, ventricles in blue and striata in grey. **d** Representative images and quantification of DCX^+^ and BrdU^+^ positive cells in the hippocampus of NestinTK^+^GCV^+^ and NestinTK^-^GCV^+^ mice after GCV treatment. DCX (green) and BrdU (red). Nuclei in blue counterstained by DAPI. Scale bar: 50 μm. *n* = 4 mice per group. Values represent mean ± SEM. **e** Renderings obtained on Neurolucida of reconstructed MSNs from a representative NestinTK^-^GCV^+^ (up) and NestinTK^+^GCV^+^ (down) mouse. **f**, **g** Sholl analysis of medium spiny neurons (MSNs) from NestinTK^-^GCV^+^ and NestinTK^+^GCV^+^ *n* = 6–8 neurons per mouse; *n* = 6 NestinTK^−^GCV^+^ and *n* = 7 NestinTK^+^GCV^+^ mice. Values represent mean ± SEM. In **f** ***p = 0.0008, ****p < 0.0001, *p = 0.020, in **g** *p = 0.03, ****p < 0.0001, ***p = 0.0008. Two-way-ANOVA test followed by Sidak post-test. **h**, **i** Striatal MSNs spine length (**h**) and spine density (**i**) in NestinTK^−^GCV^+^ and NestinTK^+^GCV^+^ mice. *n* = 6 NestinTK^−^GCV^+^ and *n* = 7 NestinTK^+^GCV^+^ mice; *n* = 6–8 neurons per mouse. Values represent mean ± SEM. In **h** *p = 0.031. Unpaired two-tailed *t*-test. **j** Spine morphology of a representative NestinTK^-^GCV^+^ and a NestinTK^+^GCV^+^ mouse. Colours are pseudo colours: dendrites (green), spine (blue) and the synaptic boutons (brown). Scale bar: 0.5 μm. **k** Quantification of MSNs labelled with DARPP32 (DARPP 32^+^ area) in NestinTK^−^GCV^+^ (*n* = 3 mice; n. total area = 34) and NestinTK^+^GCV^+^ (*n* = 5 mice; n. total area = 53) mice. Values represent mean ± SEM. Unpaired two-tailed *t*-test. Source data are provided as a Source Data file.

To investigate the effects of eNPC ablation in the SVZ on nearby striatal neurons, we used a transgenic mouse model expressing thymidine kinase (TK) under the second intron enhancer of Nestin^23^. In these mice (i.e., NestinTK^+^), the SVZ-eNPCs can be ablated upon subcutaneous ganciclovir (GCV) administration. After administration of GCV for 4 weeks in NestinTK^+^ mice (i.e., NestinTK^+^GCV^+^), we observed a substantial reduction of neuroblasts (DCX^+^ cells) and transit-amplifying cells (BrdU^+^ cells, see also labelling protocol) in the SVZ compared to NestinTK^−^GCV^+^ mice (Fig. 1a–c). Untreated transgenic control mice (NestinTK^+^GCV^−^) did not display any neurogenesis impairment compared to wild-type controls^23^. Neurogenesis in the dentate gyrus and the hippocampal function, as previously shown, were not overtly affected in this transgenic model (Fig. 1d)^23^. Ablation of NPCs in the NestinTK^+^GCV^+^ mouse did not reduce the number of neurons in the olfactory bulbs (Supplementary Fig. 1a). Further eNPC ablation was not associated with overt ependymal cells reduction (Supplementary Fig. 1b, c), neuronal apoptosis (Supplementary Fig. 1d), inflammation (Supplementary Fig. 1e), astrocytosis, (Supplementary Fig. 1f), or blood-brain barrier permeability (BBB) impairment (Supplementary Fig. 1g) in the adjacent striatal tissue, when compared to control mice.

Since local neurotransmitters from the striatum regulate eNPC proliferation as well as neuroblast proliferation and migration^24^, we questioned whether eNPCs might vice versa regulate striatal function. We thus first investigated morphological alterations in striatal neurons after eNPC ablation. A morphometric Sholl analysis performed on Golgi-stained MSNs in the striatum of NestinTK^+^GCV^+^ mice revealed a significant increase in the number of dendritic intersections that was paralleled by an increased total length of dendrites (Fig. 1e–g) compared to control mice^25,26^. Moreover, the average dendritic spine length was increased in NestinTK^+^GCV^+^ mice (Fig. 1h), while the spine density remained unchanged (Fig. 1i). In addition, using EM analysis, we observed that most neuronal spines of striatal neurons in  NestinTK^+^GCV^+^ mice displayed an elongated, filopodia-like morphology compared to spines from control mice (Fig. 1j). Moreover, we didn’t observe any difference in the number of medium spiny neurons between NestinTK^+^GCV^+^ and control mice (Fig. 1k).

Increased spine length and augmented short dendritic ramifications of MSNs from NestinTK^+^GCV^+^ mice strongly indicate that the ablation of eNPCs in the SVZ affects neuronal structure. As an additional confirmation of these results, neuronal modifications were reverted to normality when NestinTK^+^GCV^+^ recovered the SVZ-eNPCs by interrupting GCV administration for 4 weeks (i.e., recovery phase)^23^ (Supplementary Fig. 2).

### SVZ-eNPCs ablation impairs performance in a learning and decision-making task

**Fig. 2:**
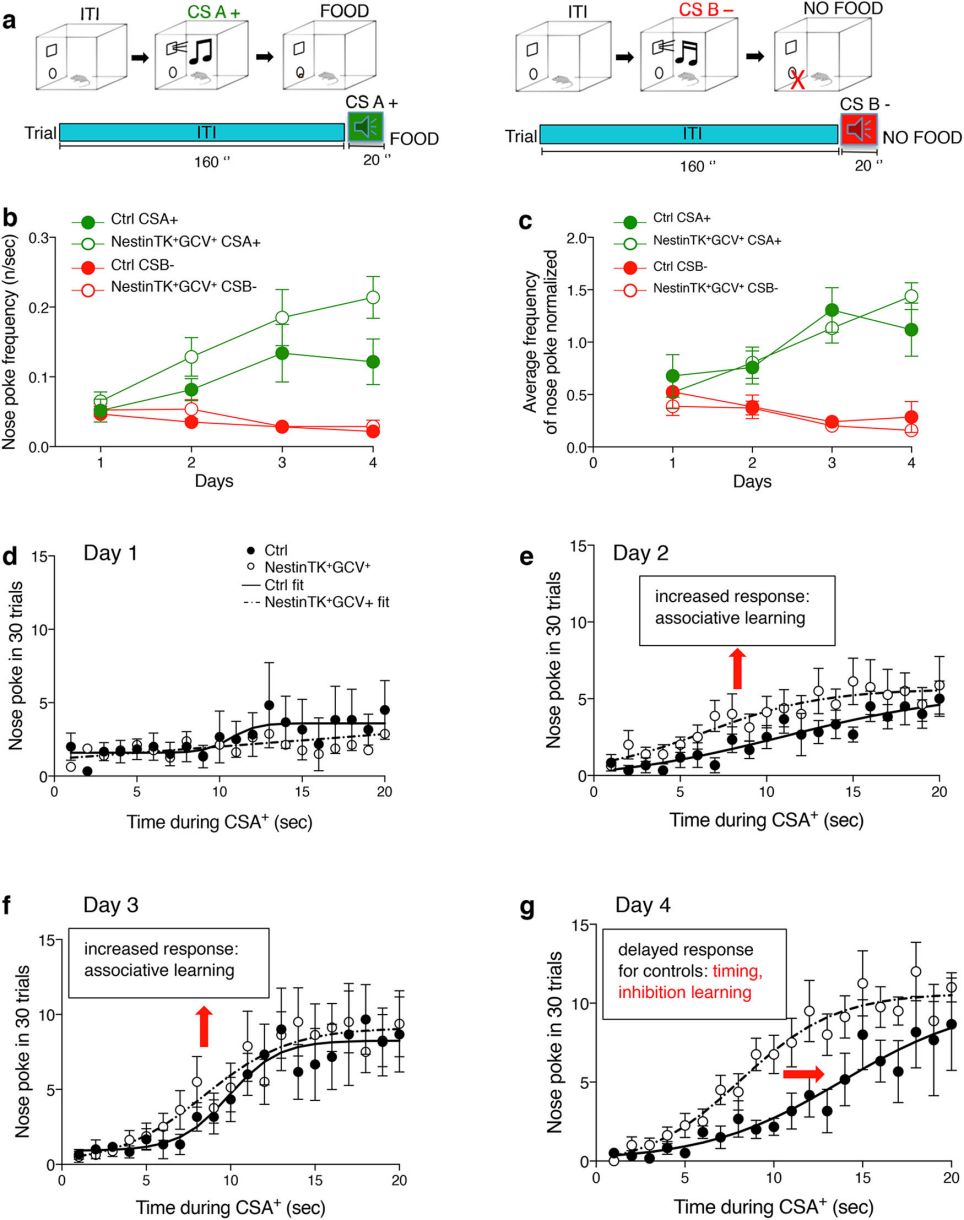
SVZ-eNPC ablation impairs performance in a learning and decision-making task in NestinTK^+^GCV^+^ mice. **a** Schematic representation of the learning protocol used. **b**, **c** The graphs show the average of nose poking frequency during the 20 s CS (A^+^ or B^-^) tone during the 4 days of test in NestinTK^−^GCV^+^ (Control mice, Ctrl) and NestinTK^+^GCV^+^ at the end of GCV administration (**b**) and the nose poking frequencies after data normalization (**c**). **d**–**g** Time course of nose pokes during the CSA+ in the subsequent days of the experiments. The total number of pokes in the 30 trials is displayed for every second during the CS. The fit lines are least squares fits to the data with sigmoid functions: R(t) = R_0_ + (R_∞_ − R_0_)/ (exp (−S* (t−t_1/2_)) +1), where R(t) is the response at time *t*, R_0_ is an initial baseline response, R_∞_ is the theoretical maximal increase in response and *S* is the maximal slope. For Days 1 and 2 the fits are meaningless, as the data essentially rise along straight lines; for the subsequent days, the parameters change as follows (control, ablated): R_0_ = (0.91, 0.10) on day 3, (0.01, −0.4) on day 4; R_∞ _= (8.26, 9.12) on day 3, (10.13, 10.64) on day 4; *S* = (0.68, 0.40) on day 3, (0.26, 0.38) on day 4; t_1/2_ = (9.89, 8.44) on day 3, (13.84, 8.18) on day 4. The slopes increase in the subsequent days indicating that the mice respond more as they associate the sound with the possibility of getting the food; by day 4 the curves for control and ablated mice clearly depart from each other: the response of control mice is delayed, and the difference is confirmed by statistical analysis of the times at which the two genotypes first trespassed half of the increase in poking frequency (11.0 ± 1.15 s in Ctrl vs. 8.5 ± 0.38 s in NestinTK^+^GCV^+^ mice). *n* = 8 NestinTK^+^GCV^+^ male mice and *n* = 6 Ctrl male mice. Values represent the mean ± SEM. Source data are provided as a Source Data file.

We next examined whether the structural alterations of MSN connections observed in NestinTK^+^GCV^+^ mice were paralleled by any impairment of striatum-dependent behaviour. A discriminative and behavioural conditioning test was employed, using conditioned auditory stimuli (CS). This test addressed the capability of discriminating between two stimuli, but also the ability to inhibit useless behaviour and defer action for an appropriate time interval^27^. During the 4 days of training the mice must learn to discern that a food pellet is delivered in association with the end of a tone A (food-reinforced conditioned stimulus: CSA+) but not a non-reinforced tone B (CSB−). In addition to this discrimination task, the animals had to learn that food was not delivered until the end of the CSA+ (20 s duration), and therefore poking the nose in the food delivery site before that time is useless (Fig. 2a).

Nose poking counts were acquired for control (NestinTK^-^GCV^+^) and NestinTK^+^GCV^+^ male mice, on 4 successive days, during every second of CSA+ or CSB− stimulus. An initial analysis through 4-way nonparametric ANOVA (see Supplementary Methods for details) indicated a significant difference between genotypes (*p* < 0.0002), between stimuli (*p* < 0.0001), from the interactions time × stimulus (*p* < 0.0001), day × genotype (*p* < 0.0004), day × stimulus (*p* < 0.0001), genotype × stimulus (*p* < 0.0082), day × genotype × stimulus (*p* < 0.0021). Poking frequencies during CSA+ and CSB− stimuli were significantly higher and lower, respectively (difference 2.86 pokes/min; *p* < 0.0001). Poking frequency in control was lower (–0.36 pokes/min) while in ablated mice were higher (+0.27 pokes/min), both significantly (*p* < 0.0001). These data indicate that both NestinTK^+^GCV^+^ and NestinTK^−^GCV^+^ control mice clearly acquired a discriminative response to the CSA+ over CSB− during the four day of training (Fig. 2b).

This initial analysis showed that the mean overall poking frequencies were significantly different between the two genotypes, as the ablated group tended to display consistently higher frequencies of nose poking (Supplementary Fig. 3a, b). This may be due to a slightly larger responsiveness or eagerness in the ablated animals, or possibly to some loss of inhibition. In fact, the poking frequencies of all animals seemed to follow the same time course during the 4 days, although with a different scale factor for each animal (Supplementary Fig. 3b). Since this is not an additive but a multiplicative factor, it would introduce spurious interactions between genotype and other factors (see Supplementary Materials). So, to avoid this and reduce variability among animals, all the analyses were repeated after normalizing the nose poking frequencies, i.e. after dividing the measurements obtained from each animal during a stimulus by the average frequency of nose poking of that same animal over all recordings (the effect of this procedure can be estimated by comparing Fig. 2b to Fig. 2c).

The results of the 4-way analysis of the number of pokes in 30 trials during the 4 days of training are reported systematically in Supplementary Table 1. They confirmed that significantly more pokes occurred with CSA+ than CSB− (*p* < 0.0001), for control as well as ablated animals, for crude values as well as ranked values (Supplementary Material). The mean value was significantly higher than the grand mean for CSA+ and lower for CSB− (difference = ±30%, both *p* < 0.0001). This confirmed that both groups of mice successfully learned to distinguish between the CSA+ and CSB− and to respond appropriately according to the sound. The number of pokes also increased from day 1 to day 4 (*p* < 0.0001), being significantly lower than the mean on day 1 (*p* < 0.0001) and significantly higher on day 4 (*p* < 0.0002); the interaction of days with the stimulus was also significant (*p* < 0.0001) as pokes increased during CSA+ and decreased during CSB- in successive days. Similarly, the change over the 20 s stimuli was significant (*p* < 0.0001), as was the interaction with the stimulus (*p* < 0.0001): poking frequency increased with time during CSA+ but not during CSB-. Further interaction was present among stimulus × day × time (*p* < 0.0008), confirming the different time courses in CSA+ and CSB−. However, no significant heterogeneity was observed between the two genotypes; also, no interactions were observed between genotype and stimulus, genotype and day, genotype and time or among genotype and various combinations of two other factors. This confirms that the interactions detected by the analysis before normalization were spurious and due to the fact that the effect of genotype was not additive but multiplicative. The absence of effects of genotype (alone or in combination with stimulus, day or time) indicates that both the ablated and control mice learned to discriminate between the two stimuli and their learning curve was superimposable, although the NestinTK^+^GCV^+^ mice displayed a generally higher poking frequency.

In order to better understand the origin of the higher poking frequency of the ablated animals, we examined the time course of nose poking during the CSA+ stimulus. In agreement with the observed higher poking frequency in ablated animals, the average time to the first nose poke during the CSA+ was significantly shorter in ablated mice when compared to control mice (7.7 ± 0.20 vs. 8.7 ± 0.26 on day 1; 8.7 ± 0.14 vs. 10.7 ± 0.42 on day 2; 9.2 ± 0.17 vs. 10.5 ± 0.20 on day 3; 9.7 ± 0.17 vs. 12.2 ± 0.22 on day 4; NestinTK^+^GCV^+^ vs. NestinTK^–^GCV^+^; Student’s *t* > 4.3, *p* ≤ 0.005 in all comparisons). On day 4, the poking frequency in response to CSA+ did not increase as much in controls as it did in ablated mice (genotype effect on day 4: *p* < 0.001, Supplementary Table 1). We therefore explored the time course of nose poking during the 20 s of CSA+ stimulus on day 4 in more detail. A two-way analysis (vs time, *p* < 0.0001, and vs genotype, *p* < 0.001) indicated a significant effect of both parameters (Supplementary Table 1); no significant interaction was apparent (*p* > 0.7179) because the effect of genotype (lower total frequency due to the delayed response) and of time (similar time dependence, just delayed in controls) accounted for most of the variance. Poking frequency increased in both groups during the first 3 days (Fig. 2d-g); however, by day 4, nose poking was significantly more delayed in the control mice than in the ablated mice (Fig. 2g): the average time by which the number of pokes in the 30 CSA+ trials first trespassed 50% of maximal (midway between the averages of the first and the last 4 s) was 11.0 ± 1.15 s in controls versus 8.5 ± 0.38 s in ablated mice (*t* = 3.09, *p* ≤ 0.005); since the frequency of poking during the CS constitutes essentially a frequency distribution, we compared the distributions of pokes during 20 s of CSA+ in the two groups using the Kolmogorov-Smirnov test on the cumulative distributions: this indicated that poking in control animals was significantly delayed, in addition to be generally reduced (*D* = 0.75, *p* = 2.6 × 10^−5^, Kolmogorov Smirnoff).

Once more, this may indicate an increased eagerness in ablated animals or a better inhibitory control during the initial phase of the stimulus, when poking would be useless.

The whole analysis was repeated in an independent cohort of animals after 1 month recovery from GCV administration (Supplementary Fig. 3c, d). The mean overall responses were no more significantly different among animals of the two genotypes (*p* < 0.533), i.e., the ablated recovery group did no more display consistently higher frequencies. After normalizing, to reduce variability among animals, the heterogeneities observed for the two stimuli, the successive days, and the different times during the CSs were all confirmed (Supplementary Table 1 and Supplementary Fig. 3d). Again, no significant differences were observed between the genotypes, except a significant effect of the interaction day × time (*p* < 0.0001: on day 2 the frequency plateaued at about 10 s instead of keeping increasing), and a significant interaction among stimulus, genotype, and day (*p* < 0.0008), due to the fact that mice performance increased more gradually, during the successive days, in controls than in the ablated recovery mice. These differences gave rise to significant differences after ranking (Supplementary Table 1), but do not suggest any obvious interpretation. In contrast with the results in the first experiment, no differences were observed between the genotypes in the time course of the frequency of poking on day 4: the time at which the number of pokes in the 30 CSA+ trials first trespassed 50% of maximal (midway between the averages of the first and the last 4 s) did not differ between controls and ablated mice: 10.8 ± 0.87 s in controls versus 10.0 ± 0.76 s in ablated mice (Supplementary Fig. 3e–h).

### eNPC ablation impairs GABAergic transmission in striatal MSNs

**Fig. 3:**
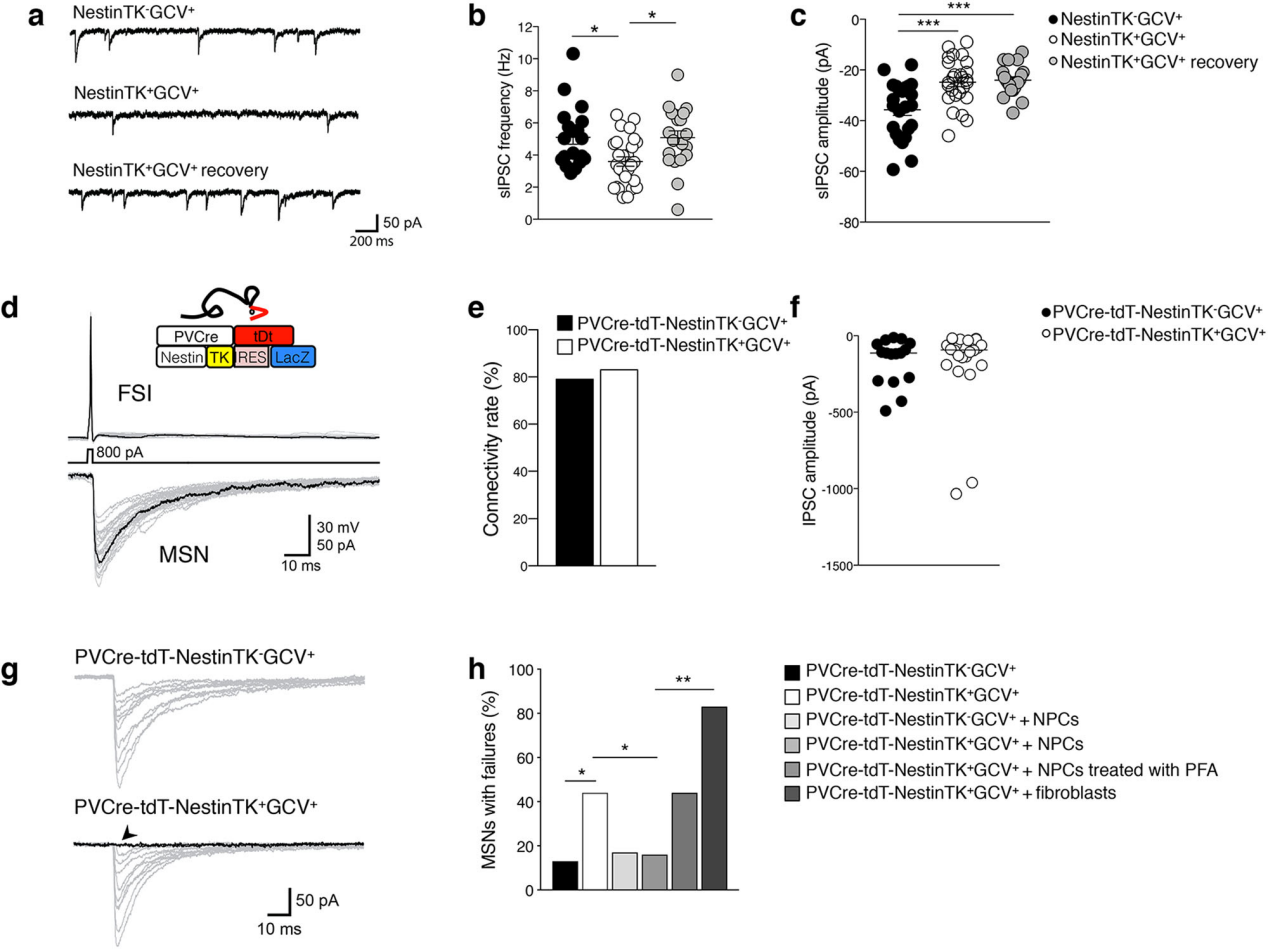
SVZ-eNPC ablation induces deficits in striatal GABAergic synaptic transmission. **a** Examples of sIPSC recordings in striatal MSNs in slices from NestinTK^−^GCV^+^, NestinTK^+^GCV^+^, and NestinTK^+^GCV^+^ mice in recovery phase. **b**, **c** Summary plots showing sIPSC frequencies (**b**) and sIPSC peak amplitudes (**c**). Values represent mean ± SEM. In **b** *p = 0.015, in **c** ***p = 0.0002. One-way ANOVA followed by Tukey post-test. *n* = 19 cells in 5 NestinTK^−^GCV^+^, *n* = 27 cells NestinTK^+^GCV^+^ in 5 mice and *n* = 20 cells in 2 NestinTK^+^GCV^+^ mice in recovery phase. **d** Schematic representation of transgenic mouse with structure of gene expression cassette. Examples of dual patch clamp recordings showing unitary IPSCs (bottom traces) evoked in a MSN in response to single action potentials (top traces) elicited by injection of a brief suprathreshold current step (800pA, 2 ms) in a synaptically connected FSI. **e** Summary histogram of connectivity rates (i.e., percentage of success in finding synaptically connected cells) for FSI-MSN pairs in PVcre-tdT-NestinTK^-^GCV^+^ (*n* = 8 mice) and PVcre-tdT-NestinTK^+^GCV^+^ mice (*n* = 12 mice) 15 out of 19 (79%), and 25 out of 30 (83%) pairs respectively. **f** Summary plot of peak amplitude for FSI-MSN pairs in PVcre-tdT-NestinTK^−^GCV^+^ and PVcre-tdT-NestinTK^+^GCV^+^ mice. Values represent medians. *n* = 16 and *n* = 24 pairs in PVcre-tdT-NestinTK^-^GCV^+^ and PVcre-tdT-NestinTK^+^GCV^+^ mice, respectively. **g** Examples of unitary IPSCs recorded in a MSN in response to individual APs evoked in a FSI. Note the presence of failures (arrowhead) in the MSN from an eNPC-ablated mouse. **h** Summary histogram with percentages of MSNs showing at least one failure in response to individual APs elicited in the connected FSI. Z-Test for 2 population proportions two-sided. *p = 0.045 PVcre-tdT-NestinTK^-^GCV^+^ vs. PVcre-tdT-NestinTK^+^GCV^+^, *p = 0.046 PVcre-tdT-NestinTK^+^GCV^+^ vs. PVcre-tdT-NestinTK^+^GCV^+^+ NPCs;  **p = 0.0021 PVcre-tdT-NestinTK^+^GCV^+^ + NPCs vs. PVcre-tdT-NestinTK^+^GCV^+^ + fibroblasts. In the histogram the number of MSNs showing at least one failure are: 2 out of 15 (13%, *n* = 8 mice), 11 out of 25 (44%, *n* = 12 mice), 1 out of 6 (17%, *n* = 2 mice), 3 out of 19 (16%, *n* = 9 mice), 4 out of 9 (44%, *n* = 3 mice) and 5 out of 6 (83%, *n* = 3 mice). Source data are provided as a Source Data file.

Changes in neuronal morphology may underlie the varied ways by which individual neurons receive and integrate synaptic inputs^28^. To investigate whether such alteration affected striatal electrical activity, we first recorded oscillations of local field potentials (LFPs) in both striata to measure striatum-striatum coherence. We observed an increased interhemispheric coherence within the 2–20 Hz frequency range in the striatum (but not in the somato-sensory cortices) of NestinTK^+^GCV^+^ (Supplementary Fig. 4). It is conceivable that some modulation of intra-striatal GABAergic transmission might have altered intra-striatal coherence. To test whether eNPC ablation affects GABAergic synaptic transmission in striatal slices, we performed whole-cell patch clamp recordings in MSNs of dorsolateral striatum to detect spontaneous inhibitory postsynaptic currents (sIPSCs) in the presence of the AMPA-receptor antagonist NBQX (5 μM) (Fig. 3a). The average frequency and amplitude of sIPSCs were significantly reduced in striatal MSNs of NestinTK^+^GCV^+^ mice compared to controls (Fig. 3b, c). In slices prepared from recovered NestinTK^+^GCV^+^ mice (2-month recovery after stopping GCV administration), the frequency of sIPSCs returned to control values, while amplitude remained reduced. Thus, eNPC ablation appeared to induce a partially reversible defect in striatal GABAergic transmission targeting MSNs.

Since sIPSCs may result from GABA release from several unidentified neighbour cell types (interneurons or other MSNs), we performed simultaneous dual whole-cell recordings in identified, synaptically connected pairs of striatal cells. Parvalbumin-expressing fast-spiking interneurons (FSIs) represent one of the principal sources of local GABAergic projections to MSNs^29^, thus we first investigated the properties of FSI-MSN pairs using dual recordings. To visually identify striatal FSIs, we crossed NestinTK mice with mice expressing Cre-dependent, floxed tdTomato (tdT) red fluorescent protein under the promoter for parvalbumin (PV; Fig. 3d). For each FSI-MSN pair, unitary IPSCs (uIPSCs) were recorded in the MSNs in response to individual action potentials (APs) elicited in the FSI. We found no changes in connectivity rates for FSI-MSN pairs from control vs. PVcre-tdT-NestinTK^+^GCV^+^ mice (Fig. 3e). Similarly, average peak amplitudes of uIPSCs were not significantly different after eNPC ablation (Fig. 3f). However, we found a significantly increased percentage of FSI-MSN pairs showing transmission failures in slices from PVcre-tdT-NestinTK^+^GCV^+^ mice as compared to controls (Fig. 3g, h). Only 13% (2 out of 15) of control FSI-MSN pairs displayed at least one failure in response to a series of 30 individual APs elicited in the presynaptic FSI. This rate was significantly larger (44%; 11 out of 25) in NPC-ablated slices. Interestingly, relative failure rates were restored to control values after incubating NPCs directly onto PVcre-tdT-NestinTK^+^GCV^+^ slices (Fig. 3h). Conversely, adding dead NPCs (pre-treated with paraformaldehyde fixation) or murine fibroblasts (3T3) did not restore the normal failure rates (Fig. 3h).

Altogether, these results suggest that eNPC ablation reduces the ability of striatal FSIs to release GABA onto MSNs. Such impairment was specific to FSI-mediated GABAergic transmission, since connectivity properties of MSN-MSN (Supplementary Fig. 5a–d) and somatostatin-expressing interneurons (SOM)-MSN pairs were unmodified after eNPC ablation (Supplementary Fig. 5e–h).

Analysis of IPSC cumulative amplitude in FSI-MSN pairs revealed a significantly smaller size of the readily releasable vesicle pool (RRP) in eNPC-ablated slices compared to controls (Supplementary Fig. 6b, c). In addition, confocal imaging analysis revealed a small, but significant reduction in PV-positive GABAergic synaptic terminals contacting putative MSN somata (Supplementary Fig. 6d–f), suggesting that reduced FSI-mediated GABA release is caused by a diminished availability of presynaptic release sites in eNPC-ablated mice.

### IGFBPL1 is reduced in eNPC-ablated mice

**Fig. 4:**
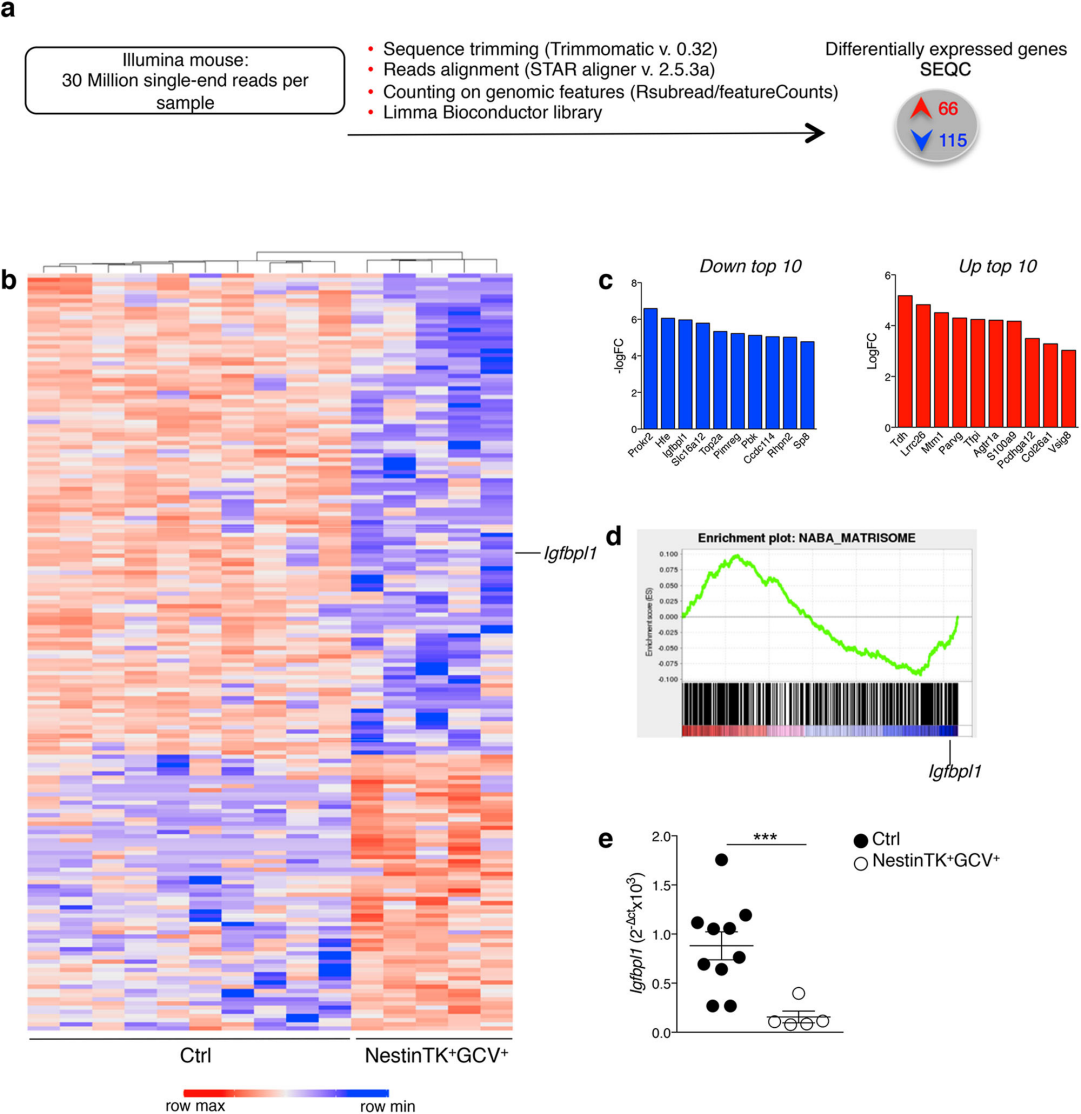
Gene expression analysis in the SVZ in NestinTK^+^GCV^+^ mice. **a** Workflow of RNA-seq gene expression analysis in control (NestinTK^−^PBS^+^, NestinTK^-^GCV^+^, NestinTK^+^PBS^+^) and NestinTK^+^GCV^+^ treated mice. **b** Heatmap with hierchical clustering of expression values of 115 downregulated genes and 66 upregulated genes with ≥2-fold change in control and NestinTK^+^GCV^+^ mice. (blue means downregulation, red upregulation) *n* = 5 mice per group. **c** The graphs represent the top 10 downregulated (in blue) and upregulated (in red) genes. **d** GSEA curve of genes expressed differentially in NestinTK^+^GCV^+^ mice compared to control mice. Enrichment plot for NABA-Matrisome. (FDR = 0.04; NES = −2.5). **e** Quantitative PCR validation of *Igfbpl1* expressed gene in control mice compared with NestinTK^+^GCV^+^ mice. *n* = 10 ctrl mice and *n* = 5 NestinTK^+^GCV^+^ mice. Values represent mean ± SEM. ***p = 0.0027. Mann–Whitney *U* test, Two-tailed. Source data are provided as a Source Data file.

To unravel possible mechanisms underlying the striatal impairment after eNPC ablation, we performed RNA-sequencing (RNA-seq) on SVZ. A differential gene expression (DGE) analysis was accomplished to compare NestinTK^+^GCV^+^ with control mice: we identified 2 sets of unique genes that were differentially downregulated (*n* = 115) or upregulated (*n* = 66) in NestinTK^+^GCV^+^ samples, setting a significance threshold of *P* ≤ 0.01 and a difference of more than two-fold (Fig. 4a–c). Gene set enrichment analysis (GSEA) identified a strong enrichment in genes involved in the matrisome, with *Igfbpl1*—a binding protein that stabilizes the Insulin-like Growth Factor-1 (IGF-1)—as one of the main downregulated genes (LogFC = −5.97) (Fig. 4d). Strong downregulation of *Igfbp1* was further validated by rt-PCR in NestinTK^+^GCV^+^ mice (Fig. 4e).

**Fig. 5:**
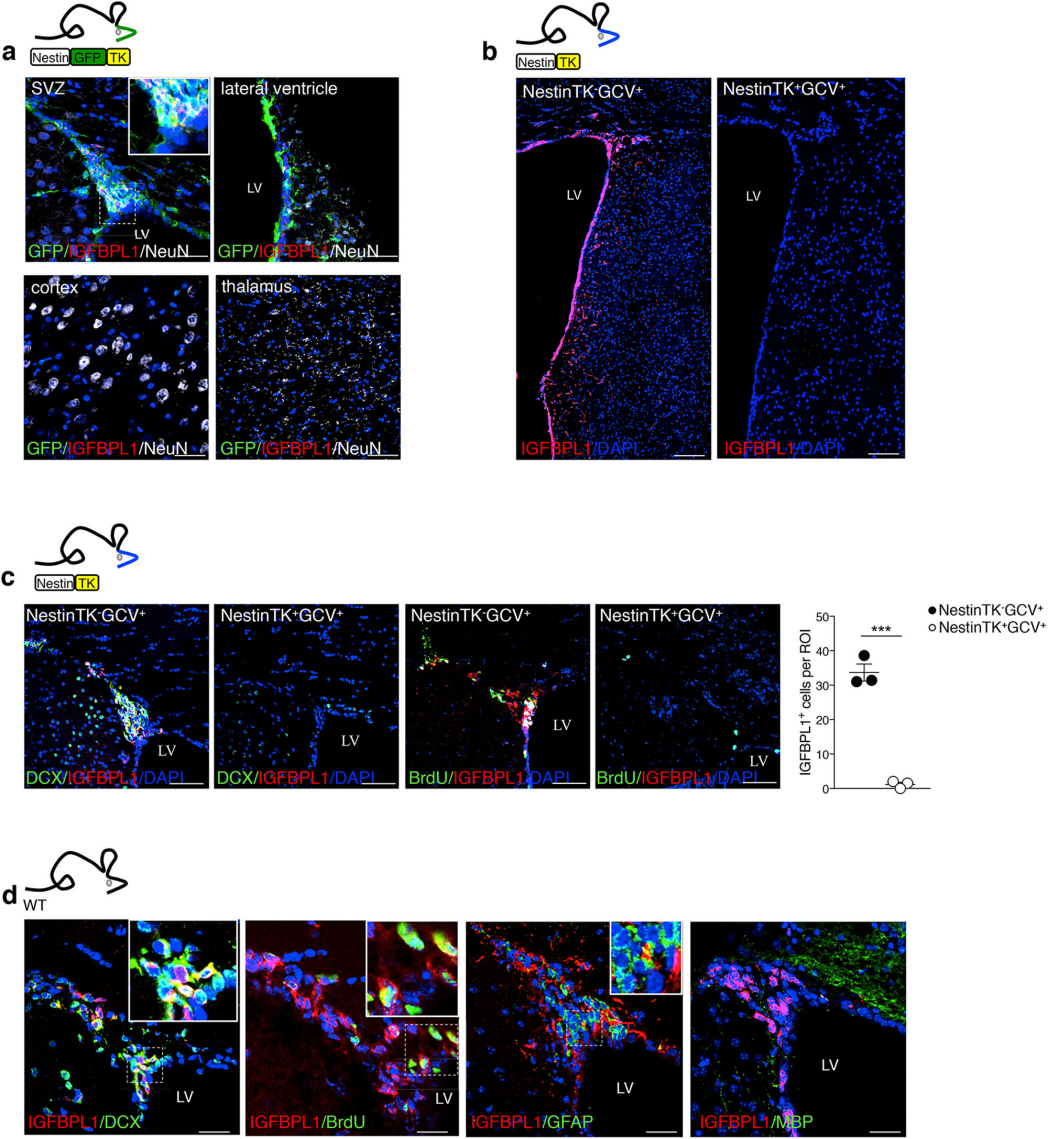
IGFBPL1 expression in the mouse SVZ. **a** Representative confocal images of NestinGFPTK coronal brain sections obtained at the level of the dorsal SVZ, of the ventral SVZ (lateral ventricle), of the thalamus and cortex. Slides were labelled for GFP in green, IGFBPL1 in red and NeuN in white. Nuclei in blue were stained by DAPI. Scale bar: 50 μm. The insert (white square) represents the double positive cells. The same result was obtained in three independent stainings. **b** Representative confocal images of the striatum and SVZ of NestinTK^-^ and NestinTK^+^ mice treated with GCV and stained for IGFBPL1 in red. Nuclei in blue were stained by DAPI. Scale bar: 100 μm. The same result was obtained in three independent stainings. **c** Representative confocal images of the SVZ of NestinTK^-^ and NestinTK^+^ mice treated with GCV and stained for DCX or BrdU in green, IGFBPL1 in red. Nuclei in blue were stained by DAPI. Scale bar: 50 μm. The graph shows the quantification of the *Igfbpl1* in the SVZ of NestinTK^-^ and NestinTK^+^ mice treated with GCV. *n* = 3 mice per group. Values represent mean ± SEM. ***p = 0.0002. Unpaired two-tailed *t*-test. **d** Representative confocal images of the SVZ of wt mice labelled for IGFBPL1 in red, DCX, BrdU, GFAP and MBP in green. Nuclei in blue were stained by DAPI. The inserts (white square) represent the double positive cells. Scale bar: 50 μm. The same result was obtained in three independent stainings. Source data are provided as a Source Data file.

We thus examined IGFBPL1 protein distribution in the mouse adult brain using immunofluorescence imaging. SVZ-eNPCs displayed a strong IGFBPL1 expression that was also detectable in the dorsal and ventral SVZ, in the striatal areas adjacent to the SVZ, and along the RMS (Fig. 5a, b and Supplementary Fig. 7a). No clear evidence of IGFBPL1 expression was found in the cortex or in the thalamus (Fig. 5a). In eNPC-ablated NestinTK^+^GCV^+^ mice, we did not find any IGFBPL1 expression in the SVZ or adjacent striatum (Fig. 5b, c). Using ELISA, we further quantified the presence of IGFBPL1 in the plasma, cerebrospinal fluid (CSF), SVZ, and striatum. While IGFBPL1 was almost absent in the plasma, it was expressed in the SVZ, in the CSF, and at lower levels in the striatum (Supplementary Fig. 7b). Conversely, IGF-1 did show a prevalence in the plasma (Supplementary Fig. 7c). To understand in detail which neural-derived cell type of the SVZ produces and releases IGFBPL1, we analysed subtypes of eNPCs such as neuroblasts (DCX^+^), type-C cells (labelled by BrdU), type-B cells (GFAP), and oligodendrocytes (MBP). We found that 45% ± 2.14% of DCX^+^, 55.4% ± 4.9 of BrdU^+^, and 5.8% ± 2.7% of GFAP^+^, but not oligodendrocytes, express IGFBPL1 (Fig. 5d). Moreover, we observed that mouse-derived neurospheres in vitro express IGFBPL1 protein (Supplementary Fig. 7d, e). The expression of *Igfbpl1* was then further examined using an independent published single-cell RNAseq dataset^22^ obtained from murine lateral and septal adult ventricular-SVZ. We focused our analysis on *Igfbpl1* expression specifically in the neural stem/precursor cell cluster (namely in adult neural stem cells—aNSC, in transit amplifying cells—TAC and in neuroblasts—NB) and in the astrocyte cluster^22^. Gene expression patterns delineated five transcriptionally distinct clusters specifically expressing genes related to astrocytes (Astro I, Astro II, Astro III, Astro IV, Astro V) and two clusters related to the neural stem/precursor cells (NB and aNSC/TC). Interestingly, 73.7% of cells within the NB cluster and 43.3% of cells within the aNSC/TAC cluster expressed *Igfbpl1*. Conversely, *Igfbpl1* expression was almost absent in each astrocyte cluster (Supplementary Fig. 8). Altogether, these data suggest that SVZ-NPCs express IGFBPL1 and that eNPC ablation abrogates this production.

### IGF-1 pathways are impaired in the striatum of eNPC-ablated mice

To assess the downstream effects of reduced expression of eNPC-secreted IGFBPL1, we analysed the expression profile of the striatum in NestinTK^+^GCV^+^ and control mice by RNAseq.

A list of 184 differentially expressed genes with a *P* ≤ 0.01 and a difference of more than twofold (Supplementary Fig. 9a, b) was generated and, according to pre-ranked GSEA, a significant enrichment was observed for the *Igf-1* Reactome pathway with genes downregulated in NestinTK^+^GCV^+^ compared to control mice (Supplementary Fig. 9c). In situ hybridization and rt-PCR showed that *Igf-1r* is expressed in the striatum of control mice and is further increased upon NPC ablation (Supplementary Fig. 9d, e). IGF-1 was instead reduced in the SVZ of NPC-ablated mice (Supplementary Fig. 9f). To identify which striatal neuronal subtypes express *Igf-1r*, we examined a published single-cell RNAseq dataset^30^ obtained from murine striatum focusing on neuronal cells. We interrogated the six main neuronal and interneuronal clusters (MSND1, MSND2, CHAT, Pvalb, Sst, and Th) for the expression of *Igfbpl1* and *Igf-1r*. While *Igfbpl1* expression was absent in neurons of the striatum, *Igf-1r* was expressed by all neuronal subtypes (Supplementary Fig. 9g–i).

### A key role of IGFBPL1 in maintaining FSI-mediated GABAergic transmission

**Fig. 6:**
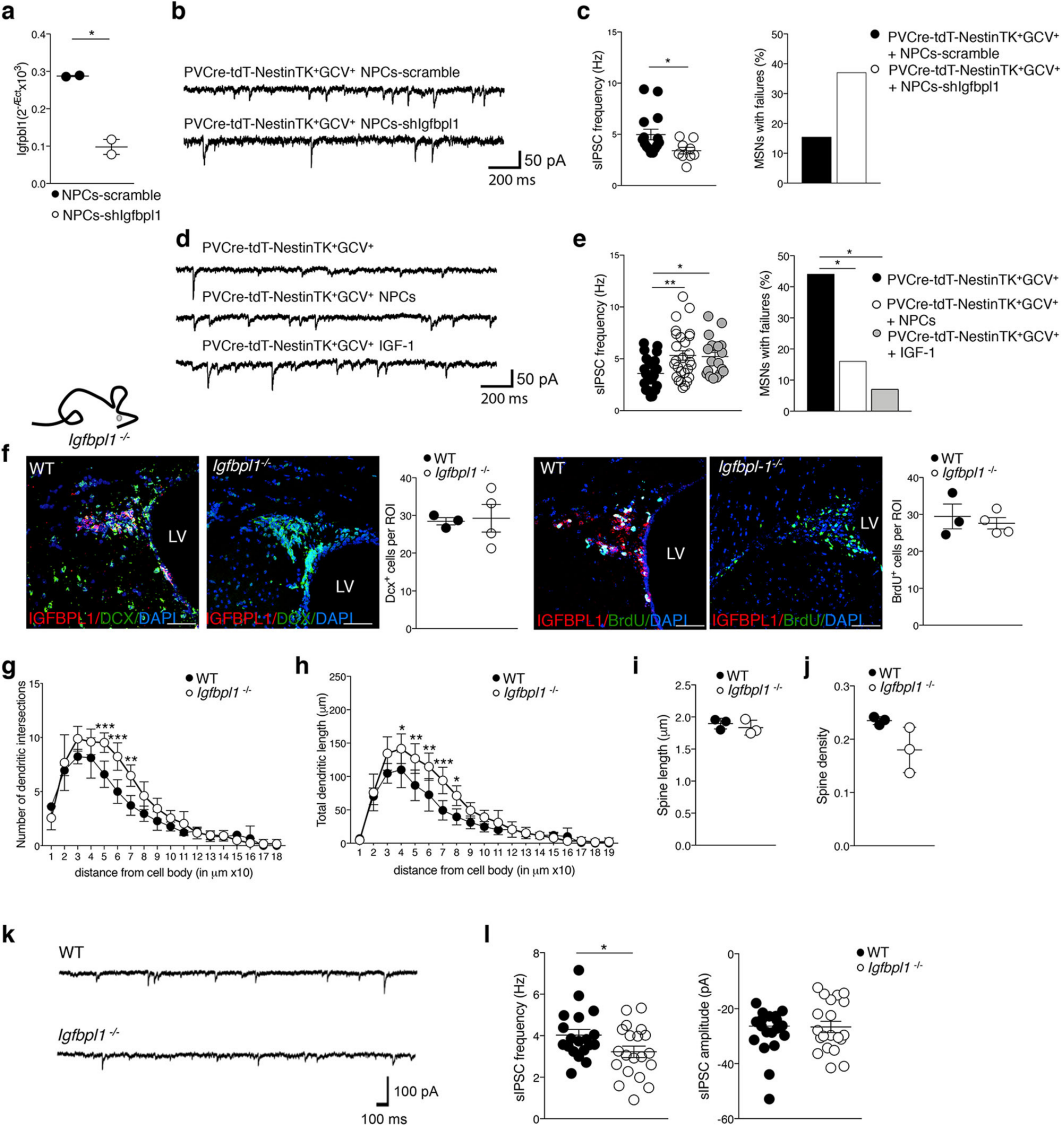
*Igfbpl1* loss of function in NPCs and *Igfbpl1*^*−/−*^ mice recapitulate morphological and neurophysiological deficits caused by SVZ-eNPC ablation. **a** *Igfbpl1* quantification in NPCs infected with a sh-*Igfbpl1* lentivirus. *N* = 2 independent infections. *p = 0.011. Unpaired two-tailed *t*-test. **b** Examples of sIPSC recordings in MSNs in slices from PVCre-tdT-NestinTK^+^GCV^+^ + NPCs-scramble and PVCre-tdT-NestinTK^+^GCV^+^ + NPC-shigfbpl1 mice. **c** Left, scatter dot plot for sIPSC frequencies. Mann–Whitney test, *p = 0.023. *n* = 5 (15 cells) PVcre-tdT-NestinTK^+^GCV^+^ + NPCs-scramble and *n* = 6 (9 cells) PVcre-tdT-NestinTK^+^GCV^+^ + NPCs-shIgfbpl1 mice. Right, histograms comparing reliability of FSI-MSN GABAergic synapses. *Z*-Test for two population proportions two-sided; *p* = 0.06 PVcre-tdT-NestinTK^+^GCV^+^ + NPCs-scramble vs PVcre-tdT-NestinTK^+^GCV^+^ + NPCs-shIgfbpl1. The MSNs showing at least one failure were 5 out of 32 (15.6%, *n* = 14 mice) and 10 out of 27 (37%, *n* = 6 mice), respectively. **d** Examples of sIPSC recordings in MSNs in slices from PVCre-tdT-NestinTK^+^GCV^+^, PVCre-tdT-NestinTK^+^GCV^+^ + NPC and PVCre-tdT-NestinTK^+^GCV^+^ + IGF-1 mice. **e** Left, scatter dot plot for sIPSC frequencies. One-way ANOVA followed by Tukey post-test; *p = 0.003 (PVcre-tdT-NestinTK^+^GCV^+^ vs PVcre-tdT-NestinTK^+^GCV^+^ + NPCs) and *p = 0.013 (PVcre-tdT-NestinTK^+^GCV^+^ vs PVcre-tdT-NestinTK^+^GCV^+^ + IGF-1). *n* = 12 (27 cells) PVcre-tdT-NestinTK^+^GCV^+^, *n* = 9 mice (27 cells) PVcre-tdT-NestinTK^+^GCV^+^ + NPCs and *n* = 3 (19 cells) PVcre-tdT-NestinTK^+^GCV^+^ + IGF-1 mice. Right, histograms comparing reliability of FSI-MSN GABAergic synapses. *Z*-Test for two population proportions two-sided; *p = 0.04 (PVcre-tdT-NestinTK^+^GCV^+^ vs PVcre-tdT-NestinTK^+^GCV^+^ + NPCs), *p = 0.032 (PVcre-tdT-NestinTK^+^GCV^+^ vs PVcre-tdT-NestinTK^+^GCV^+^ + IGF-1). From left to right, MSNs showing at least one failure were 11 out of 25 (44%, n = 12 mice), 3 out of 19 (16%, n = 9 mice), and 1 out of 15 (7%, *n* = 3 mice). **f** Representative images and quantification of neurogenesis in WT C57Bl/6 and *Igfbpl1−/−* mice: neuroblasts DCX^+^ and BrdU^+^ transient amplifying cells (in green), IGFBPL1 in red. Nuclei counterstained by DAPI. Lateral ventricle (LV). *n* = 4 mice per group. Scale bar: 50 μm. **g**, **h** Sholl analysis MSNs from C57Bl/6 and *Igfbpl1−/−* mice. Number of dendritic intersections (**g**), and total dendritic length plotted at increasing distance from the cell body (**h**). In **g** ***p = 0.0008, ***p = 0.0001, **p = 0.002; in **h** *p = 0.0473, **p = 0.0029, **p = 0.0014, ***p = 0.0005, *p = 0.0465 Two-way-ANOVA test followed by Sidak post-test. *n* = 3 WT mice and *n* = 6 *Igfbpl1−/−* mice (*n* = 6–8 neurons per mice). **i**, **j** Striatal MSNs spine length (**i**), and spine density (**j**) in C57Bl/6 and *Igfbpl1*^*−/−*^ mice. *n* = 3 mice per group. **k** Spontaneous IPSCs recorded in WT and *Igfbpl1*^*−/−*^ mice. **l** Average IPSC frequencies and amplitude measured in WT C57Bl/6 and *Igfbpl1*^*−/−*^ mice. *p = 0.0435, Unpaired two-tailed *t*-test. *n* = 3 mice for group; 19 cells for WT and 20 cells for *Igfbpl1*^*−/−*^ mice. All values represent mean ± SEM. Source data are provided as a Source Data file.

To verify the role of IGFBPL1, we generated an aNPCs line infected with a lentivirus expressing a short interfering RNA for *Igfbpl*1 (NPCs-shIgfbpl1) (Fig. 6a). We then applied NPCs-shIgfbpl1 on striatal slices obtained from NPC-ablated NestinTK^+^GCV^+^ mice. The average sIPSC frequency recorded in MSNs of NestinTK^+^GCV^+^ mice was significantly decreased after incubation with NPCs-shIgfbpl1 compared to the incubation with NPCs-scramble (Fig. 6c), while the rate of MSNs showing failures in FSI-MSN pairs increased after addition of NPCs-shIgfbpl1 (Fig. 6c).

Moreover, the average sIPSC frequency recorded in MSNs of NestinTK^+^GCV^+^ mice was significantly increased after incubation with NPCs or IGF-1 (10 nM) (Fig. 6e). Accordingly, the rate of MSNs showing failures in FSI-MSN pairs decreased after addition of NPCs or IGF-1 (Fig. 6e).

To confirm the crucial role of IGFBPL1 in the striatal functionality, we used a transgenic *Igfbpl1*^*−/−*^^21^ mouse line in which neurogenesis in the SVZ is maintained, but eNPCs do not express IGFBPL1 (Fig. 6f). *Igfbpl1*^*−/−*^ mice mimicked what we observed in NestinTK^+^GCV^+^ mice, as they showed a significant increase in both dendritic intersection number and total dendritic length compared to wild-type mice (Fig. 6g, h). Average spine length and density remained unchanged (Fig. 6i, j). The average sIPSC frequency recorded in MSNs was significantly lower in *Igfbpl1*^*−/−*^ mice than control mice, while the amplitude did not change (Fig. 6k, l). These data suggest that NPCs regulate the function of FSI- MSNs synapses through IGFBPL1.

### IGFBPL1 is expressed in human NPCs

**Fig. 7:**
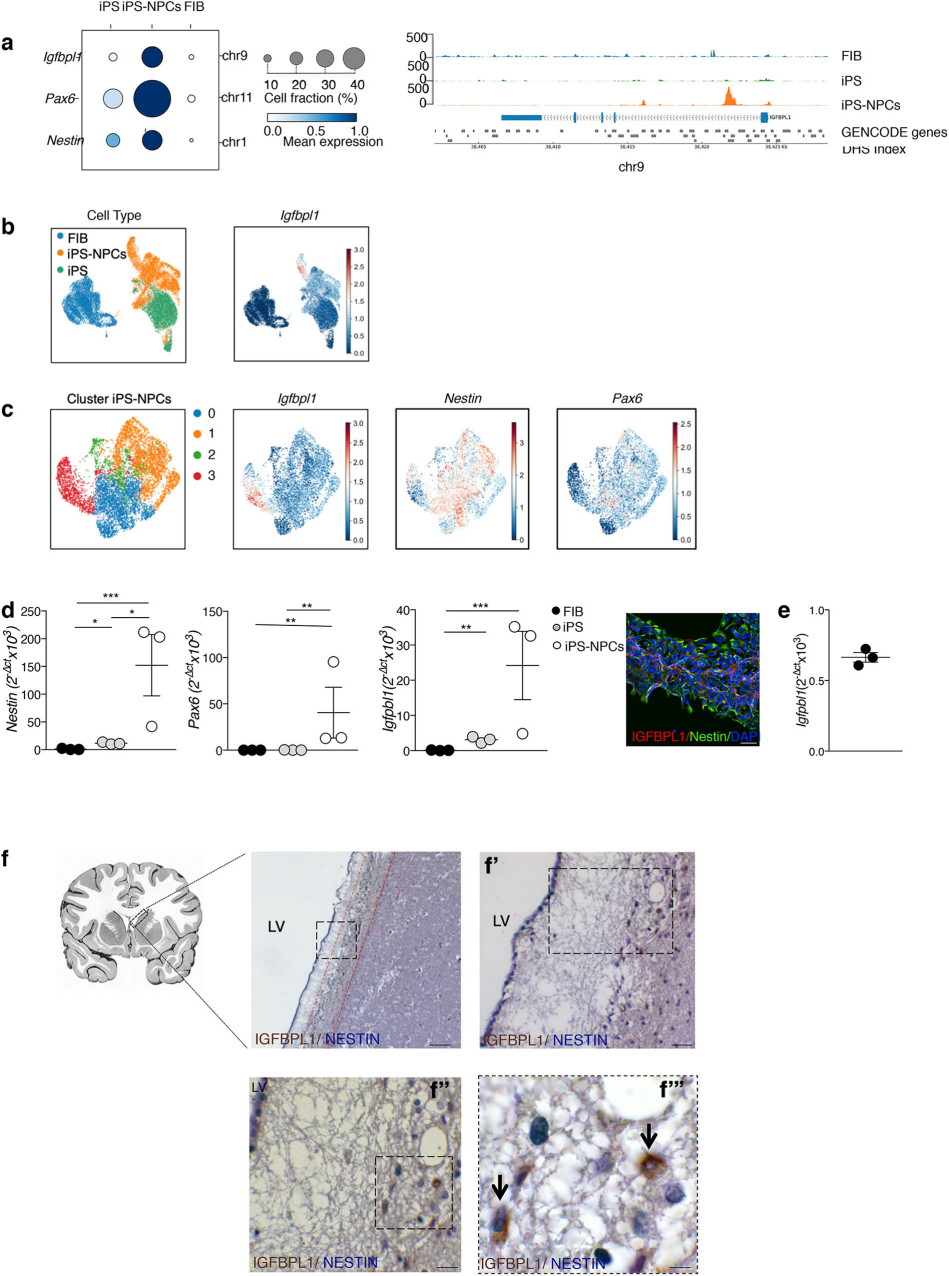
IGFBPL1 in the human SVZ. **a** In left panel, dot plot representing the gene activity of selected genes in three cell populations (FIB: fibroblasts, iPS: induced Pluripotent Stem cells, iPS-NPC: Neural Precursor Cells derived from iPS); each gene is marked by its gene symbol and the genomic coordinates used to derive the gene activity score and the dot size is proportional to the fraction of cells with non-zero signal in the population. Colour code and bubble size represent the mean intensity and % cell fraction of the gene activity score, respectively. In right panel, pseudobulk scATAC-seq profiles over *Igfbpl1* locus in three cell populations; each track represents the average ATAC-seq signal of two individuals in separate cell populations and data values are scaled to the number of cells used to derive the profile. In addition to the *Igfbpl1* gene structure, the set of DNAseI Hypersensitive Sites (DHS Index) is also represented. **b** UMAPs showing the three different populations: fibroblast, iPSs and iPS-derived NPCs and the expression of *Igfbpl1*. **c** UMAPs showing scRNAseq clusters of iPS-NPCs expressing different levels of *Igfbpl1, Nestin* and *Pax6*. **d** Quantitative PCR of *Nestin*, *Pax6* and *Igfbpl1* expressed gene in fibroblasts, iPS and iPS-derived NPCs obtained from three different healthy subjects (*n* = 3 biologically independent samples). Values represent mean ± SEM. *p = 0.0169, ***p = 0.0008, *p = 0.0369 for *Nestin*; **p = 0.0042, **p = 0.0086 for *Pax6*; **p = 0.0066, ***p = 0.0009 for *Igfbpl1*. One-way ANOVA followed by Tukey post-test. Immunofluorescent staining showing IGFBPL1 (in red) and NESTIN (in green) expression in iPS-derived NPCs from healthy subject. Scale bar: 20 μm. **e** Quantitative PCR of *Igfbpl1* expressed gene in human foetal NPCs. Values represent the mean ± SEM of three technical replicates. **f** Representative human periventricular brain section showing the SVZ cells lining the ventricle consistently expressing IGFBPL1. IGFBPL1 (brown) and NESTIN (blue) double positive cells are shown. Counterstaining with haematoxylin. Different magnifications are represented in **f’** (×20), **f”** (×40) and **f”’** (×100). The ribbon containing NPCs is highlighted in red. Scale bar: 100 μm in **f**, 500 μm in **f’**, 25 μm in **f”** and 10 μm in **f”’**. *n* = 2 samples. Source data are provided as a Source Data file.

Analysis of human NPCs is known to be difficult due to the scarce availability of brain tissue. We thus employed several different strategies to investigate *Igfbpl1* expression by human NPCs. First, we assessed the activation of *Igfbpl1* downloading scATAC-seq data for three cell populations (fibroblasts, iPSs, iPS-NPCs) derived from three individuals. We calculated gene activity scores, defined as the sum of the scATAC-seq signal over the gene body extended 2 kb upstream the TSS^31^, for *Igfbpl1*, *Nestin* and *Pax6*. We found that *Igfbpl1* activity is particularly high in iPS-NPCs (Fig. 7a, left panel). We also analysed the pseudobulk ATAC profiles over *Igfbpl1* in the three cell populations, revealing an increased accessibility in regulatory elements in the first intron of *Igfbpl1*. By looking at the annotation of the DNAseI Hypersensitive Sites catalog^32^ we found that the enrichment occurs over regulatory elements annotated for the “Primitive/embryonic” and the “Neural” components (Fig. 7a, right panel). Consistently, scRNAseq revealed that iPS-derived NPCs expressed *Igfbpl1* more abundantly compared to fibroblasts and iPSs (Fig. 7b). Cluster analysis confirmed that iPS-NPCs express *Igfbpl1*, *Nestin* and *Pax6* (Fig. 7c). Further analyses at mRNA and protein level confirmed that iPS-NPCs consistently express *Nestin*, *Pax6* and *Igfbpl1* m-RNA and produce IGFBPL1 (Fig. 7d). Accordingly, human foetal derived NPCs do express *Igfbpl1* (Fig. 7e). Finally, we analysed the presence of IGFBPL1 within the SVZ on human brain autoptic specimens obtained from two individuals. IGFBPL1 was expressed in the ribbon of SVZ lining the lateral ventricles and co-expressed with Nestin (Fig. 7f). Thus, human NPCs do express IGFBPL1.

### SVZ damage is associated to cognition impairment in patients with multiple sclerosis

To understand whether SVZ functional decline is also associated with cognitive impairment due to striatal dysfunctions in humans, we focused on multiple sclerosis (MS) patients displaying early lesions in the periventricular area with significant tissue damage. Indeed, the presence of focal lesions abutting lateral ventricles, hence possibly involving the SVZ, is a hallmark of MS. Moreover, cognitive alterations are reported in up to 50% of patients at disease onset^33^, with information processing speed—an integral and relevant component of the decision-making circuit^34,35^—undergoing the earliest impairment.

We first investigated whether, similarly to healthy subjects, iPS-derived NPCs from MS patients could express IGFBPL1. Indeed, as in healthy subjects, iPS-derived NPCs obtained from skin fibroblasts from three MS patients expressed IGFBPL1 at both mRNA and protein levels (Supplementary Fig. 10a). Thus, we performed an association study by assessing the focal and microstructural damage of the SVZ-eNPC and striatal areas with magnetic resonance imaging (MRI) and cognitive dysfunction using the Symbol Digit Modalities Test (SDMT) in a large cohort of MS patients. SDMT was selected as it is the neuropsychological item most sensitive to cognitive impairment in MS^36^ and the most administered test in MS clinical trials evaluating cognitive disability^37^. It is routinely used to acquire information about processing speed, attention, and executive functions, all crucial components of the decision-making process. Ninety-seven MS patients (mean age 36.8 ± 7.56 years, female/male [F/M] = 55/42, median EDSS 2.0, median disease duration 5.0 years) and 43 age- and sex-matched healthy controls (HC, 34.8 ± 6.52 years, F/M = 19/24) underwent a 3.0 T brain MRI and SDMT testing. HC values of SVZ microstructural integrity were used as reference to detect SVZ microstructural damage in MS. Then, age-, sex- and phenotype-adjusted partial correlations and stepwise multiple linear regression models were run to identify predictors of head-size normalized caudate volume and SDMT z-scores in the MS cohort.

Starting from anatomical ref. 38, a mask of the SVZ was obtained from high-resolution T1-weighted images in a standard space (Montreal Neurological Institute space) and then co-registered on native T1- and diffusion-weighted sequences (Supplementary Fig. 10b–e). The percentage of SVZ volume occupied by lesions was defined as SVZ percentage lesion volume (SVZ percentage LV) (Supplementary Fig. 10f).

In MS, the mean SVZ percentage LV was 4.2%. Compared to HC, in MS patients, the SVZ normal-appearing tissue was characterized by increased mean diffusivity (0.89 vs 0.86, *p* = 0.04) and preserved fractional anisotropy. Supplementary Table 2 reports the results of the age-, sex- and phenotype-adjusted partial correlations between caudate volume (normalized for head size) and measures of global and SVZ brain damage. Briefly, although most of the correlations were significant (r absolute values range: 0.33–0.54; *p* range: ≤0.0001–0.001), the stepwise multiple linear regression selected the normalized brain volume (standardized estimate 0.32, *t* value 2.92, *p* = 0.004), SVZ percentage LV (standardized estimate −0.16, *t* value −2.17, *p* = 0.03), mean cortical thickness (standardized estimate 0.31, *t* value 3.34, *p* = 0.001) and SVZ intralesional mean diffusivity (standardized estimate −0.24, *t* value −2.78, *p* = 0.007) as independent predictors of caudate volume (*R*^2^ = 0.58, root mean standard error [MSE] = 0.87). See supplementary Table 3 for further details.

In MS patients, the median SDMT performance, rated as education-corrected z-scores^39^ was −0.28 (interquartile range: −1.32; 0.48). Age-, sex- and phenotypes-adjusted partial correlations disclosed a significant association with measures of regional injury in the SVZ (percentage LV, intralesional fractional anisotropy and normal appearing tissue mean diffusivity, *r* absolute values range: 0.23–0.31; *p* range:0.03–0.003), but not with those of global brain damage, except for the T2-hyperintense lesion volume (*r* = −0.30, *p* = 0.005). Similarly, the age-, sex- and phenotypes-adjusted stepwise multiple linear regression analysis selected the SVZ percentage LV as the only independent predictor of SDMT performance (standardized estimate −0.30, *t* value −3.22, *p* = 0.002, *R*^2^ = 0.26, root MSE = 1.21), surpassing all other measures of cerebral damage, including brain volume, grey matter volume, white matter volume and cortical thickness (see supplement Tables 4, 5). Altogether our results indicate that SVZ damage is strongly associated with the impairment of information processing speed. Although there are limitations to this analysis and it is not possible to infer a causal relationship between SVZ damage and cognitive decline, these correlative data seem to support the idea that the SVZ might contribute to the regulation of cognitive processes also in humans.

## Discussion

In rodents, the SVZ has been described mainly as a source of newly formed neurons able to replace granule cells in OB upon migration along the rostral migratory stream^40^. However, considering the peculiar position of the SVZ very close to the basal ganglia and the evidence supporting non-neurogenic roles for SVZ-eNPCs in both rodents and humans occurring in response to brain tissue damage^41^, we hypothesized that SVZ-eNPCs could also exert non-neurogenic functions in physiological conditions^42^.

Here, we demonstrate that SVZ-eNPCs represent a crucial player in shaping the morphological structure and regulating the synaptic functionality of the main neuronal populations residing in the striatal area. Several data support this conclusion. In SVZ-eNPC ablated mice, MSNs showed a significant increase in the number of dendritic intersections, an increase of total length of dendrites and spine and an elongated, filopodia-like, morphology. Since neuronal morphology and dendrite outgrowth can be sculpted on the presynaptic inputs that the neuron receives, it is plausible that the altered GABAergic transmission might have modified the dendritic complexity of the medium spiny neurons as has been demonstrated in previous works^43,44^. Indeed, the morphological alterations were paralleled by a functional derangement of striatal neurons as we observed an increase in glutamatergic currents^23^ and an impairment in GABAergic transmission onto MSNs due to a decreased ability of FSIs to release GABA (this study). In addition, these electrophysiological dysfunctions may potentially contribute to the augmented intra-striatal oscillatory coherence that we observed using LFP recordings. Network oscillations encode many aspects of information processing in the brain^45–47^. Since FSI-mediated, feed-forward inhibition may create narrow time windows for synaptic integration by MSNs and help switch between different striatal network states^48^, a significant increase in GABA release failures in FSI-MSN pairs may interfere with correct striatum-dependent information processing encoded by network oscillations and consequently affect behaviour. Collectively, our data suggest that the absence of SVZ-eNPCs causes defects in striatal FSIs, a population of cells playing a crucial role in decision-making tasks^20^ and representing one of the principal sources of local GABAergic projections to MSNs. Our results are consistent with previous studies showing how striatal PV interneurons regulate striatal-based behavioural tasks^49–51^. The balance between inhibitory and excitatory synaptic transmission is essential in regulating mood and feeding behaviour and learning, memory, and appropriate behavioural responses to external stimuli^52^. Interestingly, it was recently demonstrated that PV interneurons are also crucial in decision-making tasks, as they influence the initial expression of reinforced responses and their contribution to performance declines with experience^20^. Remarkably, the results of the behavioural tests indicate that associative and discriminative learning are not impaired in NPC-ablated mice, but the balance between facilitation and inhibition in controlling a motivated behaviour seems to be slightly impaired. In our experiments, the CSA+ sound indicated that food would be available, but only at the end of the 20-s stimulus; the ablated animals seemed more impatient, poking their nose more frequently during the 20 s, as if they were more eager to find the food—or less inhibited; on day 4, control animals, but not ablated ones, visibly delayed their futile poking into the empty food tray, as if an appropriate inhibitory control were correctly operating in controls but had not ensued in ablated animals. Whether the difference consists in more eagerness or impaired inhibition of the conditioned behaviour as long as it is useless in the ablated animals, this is consistent with a defective function of the basal ganglia. Moreover, this is coherent with our neurophysiological findings in the dorsal striatum that is known to be involved in selecting among differentially reinforced behaviours (driven by dopaminergic input from the nigro-striatal path) and in inhibiting unnecessary ones. On the other hand, the ventral striatum primarily mediates motivational processes such as reward and hedonia and the reward circuit partially overlaps in both ventral and dorsal striatum^53^. Interestingly, a recent study has shown that an increase in NPC-mediated adult neurogenesis within the SVZ improves the discrimination of highly similar odorants^54^.

We then explored the molecular mechanism(s) sustaining SVZ-eNPC non-neurogenic functions, such as the abovementioned homeostatic control of striatal activity. Employing a gene expression profile study, we found in the striatum of SVZ-eNPC ablated mice a downregulation of the *Igf-1* pathway. IGF-1 is a growth factor involved in several processes in the brain (i.e., neuronal cell proliferation, survival, axonal growth, and synaptogenesis)^55^ and it is highly present in the plasma and can be produced by the choroid plexus^56^ in the CSF. IGF-1 paracrine and autocrine modes of action are modulated by IGF-binding proteins^57^. Using a RNAseq approach, we identified which component of the *Igf-1* pathway was dysregulated in the absence of SVZ-eNPCs. Indeed we found that the *Igfbpl1* gene was downregulated in SVZ-eNPCs of ablated mice. To further support this evidence at the protein level, we then demonstrated that, in wild-type mice, IGFBPL1 is expressed by eNPCs in the SVZ as well as along the migration of neuroblasts from the SVZ to the olfactory bulbs. IGFBPL1 was not expressed at the protein level by mature neuronal cells and ablation of SVZ-eNPCs reduced the levels of IGFBPL1. Unlike IGF-1, overall IGFBPL1 is present at very low levels in the plasma but highly present in the SVZ and CSF. Our results are corroborated by recent evidence indicating that, at a single-cell level, *Igfbpl1* is expressed in the adult murine SVZ by actively dividing NPCs as well as in transit-amplifying cells and in neuroblasts^22^. In the data set we analysed^30^, we did not find cells expressing *Igfbpl1* in the striatum. However, low level of *Igfbpl1* expression can not be excluded since cells expressing low level of genes are often filtered out in the single cell analysis. This might explain the difference compared to other studies relying on different techniques (e.g., in situ hybridization).

Several pieces of evidence support an active role of IGFBPL1 in brain functioning. Secreted IGFBPL1 has been shown to be involved in axonal growth via the stabilisation of IGF-1 binding to its receptor^21^. Transgenic mice overexpressing IGFBPL1 in the brain have impaired brain growth and reduced glial cell proliferation in response to injury^58^. We thus hypothesized that the lack of such protein might be the culprit of the alterations observed before, also considering previous data showing that SVZ-eNPCs express proteins that, in turn, do exert homeostatic neuroprotective functions within the brain^59^. To further analyse the effective role in vivo of IGFBPL1, we first examined *Igfbpl1*^*−/−*^ mice. The same morphological and functional alterations observed in the striatum of SVZ-eNPC ablated mice were recorded in *Igfbpl1*^*−/−*^. Slice patch-clamp studies supported this evidence since, on one hand, the absence of *Igfbpl1* in NPCs was unable to rescue the IPSCs frequency and the number of FSI-mediated GABA transmission failures. On the other hand, the administration of recombinant IGF-1 rescued the electrophysiological deficits. Altogether, these data suggest that IGF-1—produced within the brain or reaching the brain from the liver through the bloodstream and choroidal plexus^60^—does reach the SVZ where it binds to IGFBPL1 released by eNPCs. In ablated mice lacking IGFBPL1, IGF-1 availability is reduced or absent, causing the functional deficit we observed in striatal GABAergic circuits.

We further tested whether human SVZ-eNPCs can express IGFBPL1. Indeed, we found that autoptic brain material, as well as human foetal NPCs and iPS-derived NPCs from healthy controls, express IGFBPL1.

We then measured the correlation between SVZ damage and cognitive impairment in patients with MS. This disease was purposely selected for several reasons. Strong demyelination and tissue loss in periventricular regions^61–63^, involving the SVZ, are hallmarks of MS. Early cognitive alterations involving the decision-making domain—i.e. impairment of information processing speed^64^—are reported in up to 50% of MS patients at disease onset^33^. Unbalanced expression of IGF-1 and its receptor, which is downregulated in MS lesions^65^, fosters tissue damage in MS patients since they contribute to myelin production by oligodendrocytes^66^. SDMT was selected as a decision-making proxy because it challenges information processing speed, attention, and executive functions. SDMT is, in fact, impaired in patients with cognitive impairment due to striatal disorders, such as patients with Huntington’s disease (HD)^67–69^ as well as asymptomatic carriers^68^ (the SDMT is included in the Unified HD rating scale as a standardized assessment of HD patients) and patients with Parkinson’s disease (PD)^70,71^. We then applied advanced MRI sequences to assess microstructural tissue integrity in 97 patients with MS. Results obtained showed that SVZ damage (i.e., SVZ percentage LV, and SVZ intralesional mean diffusivity) is an independent predictor of caudate volume (*R*^2^ = 0.58) and that information processing speed, correlated only with the regional injury of the SVZ. While our data support the concept that human SVZ might exert a relevant role in cognitive processes and that such a homeostatic role is impaired in patients showing pathological SVZ damage, we cannot establish a causative role of SVZ in supporting cognitive function. Despite our careful correlation and model analysis we cannot completely exclude that other MS-related factors might have influenced the observed results.

In conclusion, we show a non-neurogenic physiological role of SVZ-eNPCs in supporting GABAergic connectivity within striatal circuits, through the production and the release of IGFBPL1. These findings suggest that SVZ-eNPCs might contribute, at least in part, to regulating striatal functioning. It is, therefore, tempting to speculate that SVZ-eNPCs might represent an integral part of the cellular component of cognitive circuits.

## Methods

All data needed to evaluate the conclusions in the paper are present in the paper and/or Supplementary Materials.

### Study approval and animals

Adult female and male C57Bl/6 (6–8 weeks old) and transgenic mice were purchased from Charles River or generated in our animal facility in SPF conditions. Experimental procedures, performed blindly, were approved by the Institutional Animal Care and Use Committee (no. 750, 798 and 1071) of the San Raffaele Scientific Institute, Milan (Italy). To selectively ablate SVZ eNPCs, we used the NestinTK transgenic mouse line, in which the thymidine kinase gene is under the control of the second intron of Nestin rat promoter^23^. Treatment of NestinTK, PVcre-tdT-NestinTK and SomChR-NestinTK mice with ganciclovir or PBS for 4 weeks (2 weeks for electrophysiological experiments), by means of a subcutaneously implanted osmotic minipump, induced ablation of Nestin-positive eNPCs. In all experiments transgenic mice are indicated as NestinTK^+^, while the control mice, that are the wild-type littermates, are indicated as NestinTK^-^. To define the areas of the brain expressing the IGFPBL1 we used NestinGFPTK transgenic mouse line, in which the NPCs are marked by green fluorescence. For the dual patch clamp recordings, we used PVcre-tdT-NestinTK obtained in our animal facility by crossing NestinTK with PVcre (JAX stock no. 008069) and Rosa26 Tomato. For the detection of somatostatin interneurons, we used SomChR-NestinTK obtained in our animal facility by crossing NestinTK with SomChR. The *Igfbpl1*^*−/−*^ line was kindly provided by Dong Feng Chen’s laboratory^21^. To label fast proliferating NPCs, mice were intraperitoneally injected with bromo-2’-deoxyuridine the first injection was done at the concentration of 100 mg/kg in saline, while the following injections were done at 70 mg/kg every two hours for a total of 12 h^23^.

### Tissue pathology

Male and female mice were deeply anesthetized by intraperitoneal injection of Avertin [2,5 g of 2,2,2 tribromoethanol (# T48402 Sigma-Aldrich), dissolved in 5 ml of 2-metil-2-butanol (Aldrich) and 200 ml of ddH_2_O]. The toe pinch-response method was used to determine the depth of anaesthesia. Mice were first trans-cardially perfused with a saline buffer (0.9% NaCl) plus EDTA [500 μl of EDTA (Sigma) 0.5 M were added to 250 ml of saline buffer] at room temperature (RT) and then with 4% paraformaldehyde (PFA; # 158127 Sigma-Aldrich) in PBS (phosphate buffered saline) 1× to preserve tissue in a life-like state. Brains were removed and post-fixed in 4% PFA overnight at +4 °C. On the following day, brains were washed in PBS 1× three times for 5 min and cryopreserved in 30% sucrose (Sigma) in PBS 1X. After 48 h brains were embedded in Tissue-Tek OCT compound (BioOptica) and frozen in isopentane in a liquid nitrogen bath. Brains were stored at −80 °C until sectioned. Coronal sections of 14 μm of thickness were obtained using a Leica cryostat (Leica CM1850), collected onto Superfrost slide and air dried overnight before storing them at −80 °C until staining.

For immunofluorescence, brain sections were air dried for at least 40 min and then rinsed 3 times for 5 min with PBS 1X. Non-specific binding sites were blocked by incubation in the blocking solution (PBS 1X containing 0.1% Triton X-100, 10% FBS, 1 mg/ml BSA) for 1 hour at RT. Sections were then incubated overnight at +4 °C with the appropriate primary antibody diluted in blocking solution in a humidity chamber. The following primary antibodies were used: goat anti DCX (Santa Cruz Biotechnology, product no: sc-8066, clone: C-18, lot: A0614, RRID:AB_2088494 (1:100)), rat anti BrdU (Abcam, product no: ab6326, clone: BU1/75 (ICR1), RRID:AB_2088494 (1:100)), rabbit anti DCX (Abcam, product no: ab77450, clone: EPR19997, RRID:AB_2088478 (1:100)), goat anti IGFBPL1 (R&D Systems, product no: AF4130, RRID:AB_2279980 (1:50)), rabbit anti GFP (ThermoFisher, product no: a11122, lot: 1789911, RRID:AB_221569 (1:500)), mouse anti NeuN (Millipore, product no: MAB377, clone: A60, lot: 3045564, RRID:AB_2298772 (1:300)), mouse anti MBP (Millipore, product no: MAB3861, clone: 12, (1:100)), rabbit anti GFAP (Dako, product no: Z0334, lot: 00019620, RRID:AB_10013382 (1:400)), rabbit anti VGAT (Synaptic System, product no: 131002, (1:500)), mouse anti PV (Sigma, product no: P3088, clone: PARV-19, RRID:AB_477329 (1:1000)), rabbit anti caspase-3 (Cell Signalling, product no: 9661, clone: Asp 175, RRID:AB_2341188 (1:100)), rabbit anti S100β (DAKO, product number: z0311, RRID:AB_10013383, (1:300)), mouse anti human IGFBPL1 (Santa Cruz, product no: sc-398875, clone: C-5 (1:100)). For double labelling, some primary antibodies were incubated simultaneously. The next day slides were washed 3 times for 5 min in PBS 1X and then incubated for 1 h at RT in the dark with appropriate fluorophore-conjugated secondary antibodies (Alexa Fluor 488, 546 and 633; 1:1000; Thermo Fisher Scientific. e.g. product no: A-11006, RRID:AB_2534074; product no: A-11055, RRID:AB_2534102, product no: A-11001, RRID:AB_2534069, product no: A-11008, RRID:AB_143165 and product no: A-11003, RRID:AB_2534071). In all immunofluorescence staining, nuclei were stained with 4,6 diamine-2-phenylindole (1:25000, DAPI, Roche) in PBS 1X for 1 min. Sections were mounted with Dako fluorescent mounting medium and subjected to fluorescence and confocal microscope analysis.

Human brain tissue samples were obtained from the Carlo Besta Neurological Institute (Milan, Italy). Research use of human tissue was in accordance with the Declaration of Helsinki (1964–2008) and the Additional Protocol on the Convention of Human Rights and Biomedicine concerning Biomedical Research (2005). We studied two autoptic samples: a 16-aged male died for myelitis with no overt brain alterations, and a 44-aged female died of myasthenia. The brain was fixed in formalin for several days and tissue sections were included in paraffin. For human tissue, we performed immunohistochemistry for IGFBPL1. Briefly, brain sections were treated with Xylene for 4 h, and then rehydrated with decreasing concentrations of ethanol (100, 70, 50%). Slides were washed in 1× PBS for 5 min twice and then incubated in 0.3% H_2_O_2_ in 1× PBS for 10 min. To prevent unspecific binding, sections were incubated with blocking solution for 1 h at RT. The sections were then incubated overnight at +4 °C with the primary antibody mouse anti IGFBPL1 (Santa Cruz, product no: sc-398875, (1:50)) diluted in blocking solution in a humidity chamber. The next day the slides were rinsed 3 times for 5 min in 1× PBS and then incubated for 1 h at RT with anti-rabbit biotinylated secondary antibody (Vector Laboratories, product no: BA-1000 (1:500)) diluted in blocking solution. Sections were then rinsed and further incubated with an avidin-biotin complex (ABC reagent, Vector Laboratories product no: PK-6100) for 1 h at RT. The solution was prepared 1 hour before use, 10 μl of solution A + 10 μl of solution B in 1 ml 1× PBS. Slides were again washed for 5 min 3 times in PBS 1X, incubated in diaminobenzidine (DAB) solution (DAB kit, Vector Laboratories product no: SK-4100) for 1 minute, washed in ddH_2_O and then dehydrated with increasing concentrations of ethanol (50, 70, 100, 100%). Finally, slices were put in xylene for 4 min and then mounted with non-aqueous DPX. Staining omitting the primary antibody was always used as negative control.

### Confocal microscopy and image analysis

Confocal (Leica SP5 equipped with 40× objective, Germany; software: Leica application suite advanced fluorescence software, version 2.7.3.9723 and Leica SP8 equipped with 40× objective) microscopy images were obtained to analyse immunofluorescence staining. Four to five coronal sections were examined for each animal in the SVZ and the other areas of the brain. For neurogenesis quantification, one systematic random series of sections per mice was stained (i.e., for all abovementioned antibodies), from the 14 μm thick cryostat coronal sections of the ablated and non-ablated mice, so that sections were spaced at 28 section intervals (280 μm) from each other. The section series represented a systematic random sample of sections that covered the entire extent of the forebrain.

Cell numbers in the right and left dorsal subventricular zone were quantified manually using Adobe Photoshop CC software on at least four sections per mouse containing the SVZ.

For immunohistochemistry staining, images were obtained using Zeiss AxioImager M2m with Nuance FX Multispectral Tissue Imaging System; acquired at 10× and 20× resolution.

Adobe Photoshop CC software version 14.0 (Adobe Systems Incorporated) or ImageJ 1.52q (NIH software).

### Stereological analysis of Golgi-stained striatal neurons

Male and female mice were anesthetized with ketamine/xylazine (*n* = 3–4 per group), the brains removed and stained using a Rapid GolgiStain™ Kit (FD NeuroTechnologies), and 60 μm coronal sections cut. Medium spiny neurons in the striatum close to the SVZ were analysed blinded in the region between Bregma 0 and −1.2 mm. Seven neurons per animal were analysed by using a 40×.

Dendritic branches and spines of the cells were traced using Neurolucida 8.0 (MBF Biosciences, Williston, VT) following the structures through the Z axis. Cell tracings were analysed in Neurolucida Explorer and using the morphometric analysis provided in the Neurolucida® Explorer software package. The morphometric analysis provided in this software package is a 3D Sholl analysis using concentric spheres with 10 μm between each sphere. The parameters that were analysed included: total dendritic length and number of dendritic intersections. Spine length and spine density was determined using the measuring tool on the StereoInvestigator software (MicroBrightField).

### Endogenous IgG extravasation assessment

To assess endogenous IgG (160 kDa) extravasation male mice were sacrificed as described in the section Tissue Pathology to obtain fixed tissue. 20 μm-thick cryostat coronal sections were cut and stained with standard immunohistochemistry protocol with goat anti-mouse IgG biotinylated antibody (1:500, Vector Laboratories). Scans of the whole slide were taken and analysed with ImageJ software (NIH, USA). Also, 20× magnification (Olympus, BX51, Japan) acquisitions were taken and the OD of a region of interest (ROI) comprising the dorso-lateral wall of the lateral ventricle and the SVZ were taken and quantified in the same way^72^.

### Electron microscopy

For EM studies ketamine/xylazine anesthetised female mice (*n* = 3/group) were perfused transcardially with 0.9% saline, followed by Karnovsky’s fixative (2% paraformaldehyde and 2.5% glutaraldehyde). The brains were removed and post-fixed in the same fixative overnight. Then, the brains were washed in 0.1 M phosphate buffer. Transverse 200 μm-tick brain sections were cut on a vibratome, post-fixed in 2% osmium for 1 h, rinsed, dehydrated, and embedded in TAAB resin (TAAB Laboratories, England, UK). The region between Bregma 0 and −1.2 mm was analyses to study striatal neurons close to the SVZ. Ultrathin sections were mounted onto slot grids for viewing using a LEO 912 transmission electron microscope, as previously described^73^.

### Discriminative and behavioural conditioning

Male mice were housed in pairs and had water freely available in the home cage. The holding room was on a 12-h light–dark cycle (lights on from 8.00 pm to 8.00 am). All experiments were carried out during the dark phase of the cycle. Food-deprived mice (maintained at 85% of their free-feeding weight) were weighed twice a day, before and at the end of the experimental session. Food restriction started one week before the beginning of the experiment and mice were fed with a restricted daily ration of food at the end of each experimental session. Before the start of an experiment mice were exposed to the food pellets (in their home cage) and were habituated to the test boxes (1 h/day for 3 days). Experiments were conducted using four identical fully automated Classic Modular Test Conditioning Chambers for Mouse (Med-Associates Product # ENV-307A). A food tray was mounted in the centre of the right wall, with an opening located 1 cm above the grid floor and connected to a pellet dispenser through which 14-mg sucrose pellets (Formula P) could be delivered (US). Head entries to the food tray were detected and recorded by infrared light-beam across the opening. A loudspeaker produced auditory stimuli (conditioned stimulus CS): two pure sounds at 4-kHz (CS A) and 9-kHz (CS B). Med-PC controlled all the experimental events and recorded the time at which events occurred with 10-ms resolution.

The procedure was repeated for four consecutive days. Two kinds of trials were administered (A^+^ and B^−^): both trials started with an Inter Trial Interval (ITI; 160 s), followed by a CS (20 seconds duration). The end of the CSA^+^ was paired with the presentation of a food pellet while CSB^-^ was not. The Med-PC software delivered 30 trials for each trial type (A^+^ or B^−^) in a random sequence for every daily session. Head entries into the food tray were measured during the whole experimental session.

The procedure employed here is a form of discriminative time-delayed behavioural conditioning. It has an appetitive Pavlovian component: the mice must associate a sound with the presentation (after a fixed time delay) of a food pellet that could be accessed by poking their nose in the hole accessing the food tray. The task is discriminative, in that the mice must be able to associate the appropriate sound to the food being presented, discriminating between the sounds that are (CSA^+^) or are not (CSB−) associated to such event. In addition, the task involves an aspect of behavioural control: the animals should learn that poking their nose during the A+ sound, before a fixed time delay (20 s) has lapsed, is useless, and should therefore learn to inhibit such behaviour.

The rationale of the test is to distinguish possible impairments in distinct brain structures: while the discriminative Pavlovian conditioning presumably involves the cerebellum, for the associative aspects, and possibly the hippocampus, for the spatial aspect, the behavioural control aspect relates to the possible eagerness or the capability of inhibiting the useless nose poking for a relatively long period (20 s) and therefore most likely involves facilitatory/inhibitory behavioural control by the basal nuclei.

To clarify the different aspects, the following parameters were measured: (i) the total number of nose pokes during 30 CS A + trials and 30 CS B^-^ trials were counted on each day in control and ablated mice, and analysed through a 4-way nonparametric equivalent of ANOVA (described in detail in Supplementary Material): nose poke number during the two stimuli (CSA + , CSB−), for the two genotypes (control, ablated) in different days (1–4) and at different times during the stimulus (1–20 s) to check the associative learning capability (performance in subsequent days) and the discriminative capability (CS A + vs. B^−^); (ii) on the last day (day 4) the time course of nose poking during the 20 s CSA+ sound was measured as the total number of pokes in 30 trials at each time during the stimulus; the time where such value trespassed the 50% threshold (midway between the average of the first and the last 4 s), as well as the average time of the first nose poke in the 30 CSA+ trials, were compared between control and ablated mice (Student’s *t* test) to test the capability of the animals to inhibit the useless behaviour of nose poking during the stimulus.

### Slice preparation and electrophysiology

All procedures were approved by the Italian Ministry of Health and were conducted in accordance with FELASA guidelines and European directives (2010/63/EU). Patch-clamp recordings were performed in sagittal striatal slices. Mice of both sexes (45–60 days of age for young mice, 18 months for aged mice) were anesthetized with an intraperitoneal injection of a mixture of ketamine/xylazine (100 mg/kg and 10 mg/kg, respectively) and perfused transcardially with ice-cold artificial cerebrospinal fluid (ACSF) containing (in mM): 125 NaCl, 3.5 KCl, 1.25 NaH_2_PO_4_, 2 CaCl_2_, 25 NaHCO_3_, 1 MgCl_2_, and 11 D-glucose, saturated with 95% O_2_ 5% CO_2_ (pH 7.3). After decapitation, brains were removed from the skull and 300 μm-thick sagittal slices containing the striatum were cut in ACSF at 4 °C using a VT1000S vibratome (Leica Microsystems, Wetzlar, Germany). Slices were then kept in a chamber containing ACSF at 31.5 °C for 15 min and slowly cooled down and maintained at 26.5 °C. Subsequently, individual slices were submerged in a recording chamber mounted on the stage of an upright BX51WI microscope (Olympus, Japan) equipped with differential interference contrast optics (DIC) and an optical filter set for the detection of tdTomato red fluorescent light (Semrock, Rochester, NY, USA). Slices were perfused with ACSF continuously flowing at a rate of 2-3 ml/min at 32 °C. Whole-cell patch-clamp recordings were performed in dorsolateral striatum (roughly 2–3 mm from pial surface, 1.5 mm interaural, and 0–1 mm from bregma) using pipettes filled with a solution containing the following (in mM): 30 KH_2_PO_4_, 100 KCl, 2 MgCl_2_, 10 NaCl, 10 HEPES, 0.5 EGTA, 2 Na_2_-ATP, 0.02 Na-GTP, (pH 7.2 adjusted with KOH, tip resistance 6–8 MΩ). Inter-somatic distances between cells selected for dual recordings were consistently <100 μm. MSNs were easily identified by eye, as these cells represent >90% of the total striatal population. The typical firing pattern of MSNs was also used to confirm their identity after patching. Conversely, FSIs (representing ~1% of the total striatal cell population) were identified after fluorescent labelling as described in the Result paragraph “eNPC ablation impairs GABAergic transmission in striatal MSNs”. All recordings were performed using a MultiClamp 700B amplifier interfaced with a PC through a Digidata 1440 A (Molecular Devices, Sunnyvale, CA, USA).

#### NPCs application

To study the effect of NPCs application on striatal circuitry we used (1) normal aNPCs, (2) aNPCs treated with PFA, (3) NPCs infected with a lentivirus of third generation expressing sh-scramble as a control for *shIgfbpl1*, and (4) NPCs silenced for *Igfbpl1* using a lentivirus expressing *shIgfbpl1*. Cells were collected and resuspended to a concentration of 10^6^ cells/50 μl and gently placed onto the surface of the brain slice at least 30 min before the electrophysiological recordings as already described^23^.

#### Data acquisition and analysis

Data were acquired using pClamp10 software (Molecular Devices) and analysed with Origin 9.1 (Origin Lab, Northampton, MA, USA). Voltage- and current-clamp traces were sampled at a frequency of 30 kHz and low-pass filtered at 2 kHz. To isolate GABA-receptor-dependent IPSCs, the ACSF was added with the AMPA antagonist NBQX (5 μM). Recordings were performed at a holding potential of −80 mV. Spontaneous IPSCs were analysed using pClamp automatic event detector. In dual whole-cell recordings, unitary IPSCs were considered spike-induced when the IPSC onset occurred at a latency of 1–2 ms form the peak of the presynaptic spike. Responses were classified as failures when presynaptic spikes induced no IPSCs.

To assess synaptic vesicle (SV) readily releasable pools (RRPs) in FSI-MSN pairs, high-frequency stimulus trains (20 Hz, 1.5 s) were applied to the presynaptic FSIs. Cumulative IPSC peak amplitudes were plotted against stimulation time and the linear phase of the dataset was fit with a straight line. Back-extrapolation of the fit line to the *Y*-axis intercept provided an estimate of the RRP^74^.

#### Photostimulation of CHR-expressing interneurons

Experiments were performed in slices prepared from a recombinant Cre-Lox mouse line obtained by crossing B6.Cg-Gt (ROSA) 26Sortm27.1 (CAG-COP4*H134R/tdTomato) Hze/J mice (JAX stock no. 012567; Jackson Laboratory, Bar Harbor, ME) with B6N.Cg-Ssttm2.1 (cre) Zjh/J (JAX stock no. 018973) mice. The offspring selectively expressed a channelrhodopsin-2 (CHR-2)/tdTomato fusion protein in somatostatin (SOM)-expressing interneurons throughout the brain. An optogenetic approach was used to selectively stimulate striatal SOM-interneurons, due to their relative sparseness and long-range connectivity^18^ which greatly reduces the success rate of dual patch recordings. Optical stimuli were generated by a diode-pumped solid-state laser (wavelength: 473 nm; light power at the source: 100 mW; Shanghai Dream Lasers Technology, Shanghai, China) connected to the epi-illumination port of the microscope through a multi-mode optical fibre. The beam was deflected by a dichroic mirror and conveyed to the slice through a 40× water-immersion objective (spot size: 0.06 mm^2^). The light power measured with an optical power meter at the level of the slice surface was ~2 mW, yielding a light density value of ~33 mW/mm^2^. Photostimuli were TTL-triggered using Clampex digital output signals.

#### Drugs

The following drugs were obtained from HelloBio (Bristol, UK): NBQX disodium salt (HB0443) and SR95531 hydrobromide (Gabazine, HB0901). IGF-1 (PeproTech; 100-11) was added to the chamber at 26.5 °C to a final concentration of 10 nM at least 30 min before the electrophysiological recordings.

### Synapses segmentation and analysis

14μm thick cryostat coronal sections were immunolabeled with vGAT and NeuN antibodies, and confocal (40× or 63× oil objectives) images were acquired. Equal-sized perisomatic regions of interest (ROIs) were randomly picked from acquired fields. Puncta segmentation was run by custom routines, as previously described^75^, on vGAT fluorescence channel. A fluorescence threshold for puncta inclusion/exclusion was set as follows: mean background fluorescence was calculated, for each field analysed, from ROIs devoid of fluorescent puncta. All the segmented objects with mean fluorescence (major than) mean background +2 SD were considered as synapses, the remaining were excluded. Synapses were considered positive for tdTomato when average fluorescence was higher than the background tdTomato fluorescence calculated as described above. Double positive synapses were than normalized over total perisomatic boutons (vGAT positive only) and represented as percentages.

### RNA extraction

Male and female mice were sacrificed under deep anaesthesia with tribromoethanol (Avertin) and transcardially perfused with cold saline solution with EDTA. Brains were rapidly removed and SVZ were dissected on ice and stored at −80 °C until RNA extraction.

Total RNA was isolated using the RNeasy MiniKit (#74104; Qiagen) according to the manufacturer’s instructions, including DNAse digestion. At the end, RNA samples were eluted from columns using 30 μl of RNase-free water and their concentrations were determined or spectrophotometrically by A260 (Nanodrop–ND1000) or using a 2100 Bioanalyzer (Agilent) resulting in RNA integrity number ≥8.

### Gene expression analysis

Semi-quantitative RT-PCR was performed using pre-developed TaqmanTM Assay Reagents on a Quantstudio 3 Real-Time PCR System (Thermofisher) according to the manufacturers’ protocol.

The cDNA was synthesized from 500 ng of total RNA using the ThermoScript IV RT-PCR System (Thermofisher) according to the manufacturer“s instructions. Two-hundred ng of cDNA were used for RT-PCR using pre-designed Taqman® Gene Expression Assays (Thermofisher). RT-PCRs were performed using the following specific assays (all Thermofisher): *Igfbpl1* (Mm01342060_m1), *Igf-1r* (Mm00802831_m1), *Dcx* (Mm00438400_m1), *Dlx2* (Mm00438427_m1)*, Igfbpl1* (Hs01390103_m1), *Pax6* (Hs00240871_m1), *Nestin* (Hs04187831_g1).

Data were collected with instrument spectral compensations by the Thermofisher Connect^TM^ software and analysed using the threshold cycle (CT) relative quantification method. In this study, GAPDH was used exclusively as housekeeping gene.

### RNA-sequencing

For SVZ tissue libraries were prepared using SMART-Seq® v4 Ultra® Low Input RNA Kit for Sequencing (Takara Bio USA) performing 10 cycles of amplification, according to the manufacturer’s instructions, while for the striatum tissue libraries were prepared using QuantSeq 3′ mRNA-Seq Library Prep Kit FWD for Illumina (Laxogene).

Sequencing was performed on a NextSeq 500 machine (Illumina, San Diego, CA) obtaining 30 million single end reads per sample on average.

Reads were trimmed using Trimmomatic, version 0.32, to remove adapters and to exclude low-quality reads from the analysis. The remaining reads were then aligned to the reference genome mm10, Gencode version M16, using STAR aligner, version 2.5.3a. The feature Counts function from Rsubread package (v 1.16) was used to assign reads to the corresponding genes. Only genes with a CPM (Counts per million) value higher than 1 in at least three samples were retained. Gene expression read counts were exported and analysed in R environment (v. 3.1.1) to identify differentially expressed genes (DEGs), using the limma Bioconductor library^76^. DEGs were identified as those genes that changed at least twofold and had a nominal *p* value lower than 0.01.

Pre-ranked Gene Set Enrichment Analysis (GSEA)^77^ was performed considering all the expressed genes. The gene-sets included in the GSEA analyses were obtained from Canonical Pathways, Hallmark and the Gene Ontology (GO) collections as they are reported in the MSigDB database (https://www.gsea-msigdb.org/gsea/msigdb/index.jsp). The accession number for these data is GSE165815.

### Assay for transposase-accessible chromatin using sequencing (ATAC-seq)

Single-cell ATAC-seq was performed on Chromium platform (10X Genomics) using “Chromium Single Cell ATAC Reagent Kit” V1 Chemistry (manual version CG000168 Rev C), and “Nuclei Isolation for Single Cell ATAC Sequencing” (manual version CG000169 Rev B) protocols. In-house produced Tn5 protein with modified Tn5ME-A sequence (manuscript under revision) was used instead of the “ATAC Enzyme” (10X Genomics). Nuclei suspension was prepared in order to recover and analyse 5000 nuclei. Libraries were sequenced on Novaseq6000 platform (Illumina) with 2 × 50 bp read length; a custom Read 1 primer was added to the standard Illumina mixture (5′-TCGTCGGCAGCGTCTCCGATCT-3′).

Reads were demultiplexed using cell ranger-atac (v1.0.1). Identification of cell barcodes was performed using umitools (v1.0.1)^78^ using R2 as input. Read tags were aligned to hg38 reference genome using bwa mem v0.7.12 [arXiv:1303.3997 (q-bio.GN)]. Gene activities were calculated as per cell coverage over the gene body interval extended to 2 kb upstream the TSS, using gencode v30 as gene model^79^. Data were processed using scanpy v1.4.6^80^. Pseudobulk tracks were generated using pyGenomeTracks^81^.

### Single-cell RNA sequencing

#### Mouse

Information about gene expression at single-cell level for murine SVZ was retrieved from Mizrak et al.^22^ (ref., GEO accession number GSE109447). The full dataset was processed with the Seurat standard pipeline in R environment (R v.4.0.3, Seurat v.3.2.2), after the removal of cells expressing less than 200 or more than 4000 genes, showing a percentage of mitochondrial genes exceeding 5%, or being annotated as “Doublets” by the authors. A bi-dimensional representation of the full dataset was obtained by applying the UMAP algorithm on the first 30 PCA components.

Cells annotated by the authors as “Astrocytes” or neural precursors (“aNSC + TAC + NB”) were extracted and further classified, performing a clustering analysis with the Louvain methods implemented in Seurat (parameters: nPCs = 30, resolution = 0.5).

The expression profiles of cell subpopulations in mice striatum tissue were further inspected taking advantage of the single-cell RNA-sequencing (scRNAseq) dataset from Munoz-Manchado et al.^30^ (ref., GEO accession number GSE97478). Only cells classified by the authors as belonging to the “CHAD”, “MSND1”, “MSND2”, “Pvalb”, “Sst” and “Th” clusters were extracted and processed with the Seurat standard pipeline in R environment (R v.4.0.3, Seurat v.3.2.2).

The Uniform Manifold Approximation and Projection for Dimension Reduction (UMAP) algorithm was applied to the first 20 principal components from PCA, while no additional clustering was performed other than the one provided by the authors.

#### Human

Single-cell RNA-seq was performed on Chromium platform (10X Genomics) using “Chromium Single Cell 3ʹ Reagent Kits v3” kit manual version CG000183 Rev C (10X Genomics). Final libraries were loaded on Novaseq6000 platform (Illumina) to obtain 50,000 reads/cells. Raw data were downloaded from ArrayExpress (E-MTAB-10220) and processed with scanpy^80^. Droplet doublets were identified with scrublet^82^. Cells with more than 20% of mitochondrial genes expressed were removed. Highly variable genes were identified (minimum mean 0.1). Total counts and percentage of MT genes were regressed out before scaling. Batch removal was performed using Harmony^83^.

### In situ hybridization

In situ hybridization was performed as previously described^84,85^. Briefly, 14 μm-thick brain sections of female mice were post-fixed 15 min in 4% paraformaldehyde, then washed three times in PBS. Slides were incubated in 0.5 mg/ml of Proteinase K (Roche) in 100 mM Tris-HCl (pH 8), 50 mM EDTA for 10 min at 30 °C. This was followed by 15 min in 4% Paraformaldehyde. Slices were then washed three times in PBS then washed in H_2_O. Sections were incubated in triethanolamine (Merk, Germany) 0.1 M (pH 8) for 5 min, then 400 ml of acetic anhydride (Sigma) was added two times for 5 min each. Finally, sections were rinsed in H_2_O for 2 min and air-dried. Hybridization was performed overnight at 60 °C with a-UTP riboprobes at a concentration of 100 ng/ul. The following day, sections were rinsed in SSC 5× for 5 min then washed in formamide 50% (Sigma)-SSC 2× for 30 min at 60 °C. Then slides were incubated in ribonuclease-A (Roche) 20 mg/ml in 0.5 M NaCl, 10 mM Tris-HCl (pH 8), 5 mM EDTA 20 min at 37 °C. Sections were washed in formamide 50% SSC 2× for 30 min at 60 °C then slides were rinsed two times in SSC 2×. After that, the sections were blocked in B1 buffer [150 mM NaCl, 100 mM Tris-HCl (pH 8)] and 10% of foetal bovine serum for 1 h. Finally, the sections were incubated overnight at +4 °C with the antibody anti-digoxigenin-AP Fab fragment (#11093274910, Roche, 1:1000) diluted in blocking solution in a humidity chamber. The next day, sections were washed for 10 min 2 times with buffer B3 [100 mM NaCl, 100 mM Tris-HCl (pH 9.5), MgCl_2_ 50 mM] and incubated in 5-bromo-4-chloro-3-indolyl-phosphate (BCIP) (Roche) and 4-nitro blue tetrazolium chloride (NBT) (Roche) overnight. The following probes were used: mouse *Igf-1r* riboprobe (gift from Giacomo Consalez, San Raffaele Hospital, Milan, Italy). Microphotographs of sections were digitalized in dark field light microscopy (Olympus BX51, and 20× objective) by using a CCD camera (Leica). To confirm the specificity of the different RNA probes, sense strand RNA probes (showing no signal) were used as negative controls. As positive control for the technic we use *plp* riboprobe (present in the laboratory).

### Mouse and human neural stem cell cultures: generation and maintenance

#### Mouse NPCs

Adult C57Bl/6 mice (6–8 weeks old, 18–20 g Charles River) were anaesthetized by an intraperitoneal injection of tribromoethanol and killed by decapitation. The parietal bones were cut in a cranial-to-caudal way using microsurgery scissors and the brain was removed and positioned in a Petri dish (Corning Costar) containing sterile PBS. Brain coronal sections were taken 2 mm from the anterior pole of the brain, excluding the optic tracts and 3 mm posterior to the previous cut. The SVZ of the lateral ventricles was isolated from the coronal section using iridectomy scissors. Tissues derived from at least two mice were pooled to generate each culture. Dissected tissues were transferred to a 15 ml tube with digestion medium [EBSS 1X (Gibco, cod#E2888), Cysteine (Sigma, cod#C7352) 200 mg/L, EDTA (Sigma, cod#E6511) 200 mg/L, Papain (Worthington, cod#P4762) 2U/ml], and incubated for 45 min at 37 °C on a rocking platform, as previously described [*59*]. At the end of the incubation, the tube was centrifuged at 200 × *g* for 12 minutes, the supernatant was removed, and the pellet was mechanically disaggregated with 2 ml of EBSS. The pellet was centrifuged again at 200 g for 12 minutes and then dissociated with a 200 μl pipette and plated in Neurocult proliferation medium (Stem cell Technology, BC, CA) supplemented with EGF (20 ng/ml) and FGF2 (10 ng/ml). Cells were plated in a 25 cm^2^ flask (Corning Costar) and incubated at 37 °C in an atmosphere of 5% CO_2_. After approximately one week, a small percentage of the isolated cells begun to proliferate, giving rise to small cellular aggregates, which are similar to rounded spheres (called neurospheres) and which grow in suspension. When the neurospheres reached the necessary dimension (about ∅ ≥ 150–200 μm diameter), the cells were harvested in a 15 ml tube (Falcon) using a sterile pipette and centrifuged at 200 × *g* for 12 min. The supernatant was then removed, 200 μl of Accumax (Sigma) was added and the tube was incubated at 37 °C for 10 min. The cells were dissociated, counted, and plated at the density of 8000 cells/cm^2^. This procedure was repeated at each passage of dissociation.

#### Mouse NPCs infected with a lentivirus expressing a short interfering RNA for Igfbpl1

NPCs were dissociated 8 h before being infected and plated at high density (1.5 × 10^6^ cells in a 75 cm^2^ flask) in 10 ml of Neurocult proliferation medium. After 8 h, NPCs were infected with 3 × 10^6 ^T.U./ml of a lentivirus expressing *shIgfbpl1*. After 48 hours the cells were harvested, centrifuged at 200 × *g* for 12 min and plated without dissociation. After 3 passages in vitro, we performed gene expression analysis to verify the downregulation of the *Igfbpl1* mRNA. We called these cells: NPCs-shIgfbpl1.

#### Human foetal NPCs

Permission to use human foetal CNS tissue was granted by the ethical committee of the San Raffaele Hospital (approval on 09/06/2016). Tissue procurement was in accordance with the declaration of Helsinki and in agreement with the ethical guidelines of the European Network for Transplantation (NECTAR). The BI-0194-008 is a non-immortalized human NPC line obtained from the diencephalic and telencephalic regions of a single human white male foetus at 10–12 weeks gestational age after pregnancy termination. The foetus was provided by Banca Italiana del Cordone Ombelicale Fondazione IRCCS Ca’ Granda Ospedale Maggiore Policlinico, Milan, Italy. None of the study participants received compensation for participation in the study.

Briefly, fresh human foetal brain tissue was chopped mechanically and dissociated with Trypsin (LONZA; BE17-161E) 1:5 in growth medium for 5–10 min at 37 °C, 5% O_2_ e 5% CO_2_. Then, it was washed with 10% Australian FBS in fresh medium and centrifuged 15 min at 200 × *g*. Finally, the cells were plated in T25 flask in NeuroCult-XF Proliferation Medium (STEMCELL Technologies; cat.# 05760) with rh-EGF (Provitro; cat.# 1325 9510 00) and rh-bFGF (Provitro; cat.# 1370 9505 00) at the final concentration of 20 ng/ml, at 37 °C, 5% O_2_ e 5% CO_2_.

After 15–25 days, enzymatic (Accumax®; Sigma) dissociation of neurospheres was performed at 37 °C for 3–5 min, of neurospheres was performed and the cells were re-plated at clonal density (20–25,000 cells/cm^2^) in NeuroCult-XF Proliferation Medium completed as mentioned above.

#### Human induced-pluripotent stem cells (iPS) derived NPCs

Permission to generate induced-pluripotent stem cells (iPS) from MS patients’ fibroblasts was granted by the ethical committee of the San Raffaele Hospital (BIOBANCA-INSPE, approved on 9 March 2017). To avoid genetic biases, fibroblasts were isolated from skin biopsies from three pairs of twins discordant for MS (the first pair of twins are females, age 34 at time of tissue donation; the second pair of twins are females, age 35 at time of tissue donation; the third pair of twins are 1 female and 1 male, age 35 at the time of tissue donation.). Fibroblasts were reprogrammed into iPSCs by using the replication-incompetent Sendai virus kit (Invitrogen) according to manufacturer’s instructions. In brief, colonies of iPSCs were detached from mouse embryonic fibroblasts by treatment with 1 mg/mL collagenase IV. After sedimentation, cells were resuspended in human embryonic stem cell (hESC) medium without bFGF2 supplemented with 1 µM dorsomorphin (Tocris), 3 µM CHIR99021 (Axon Medchem), 10 µM SB-431542 (Ascent Scientific) and 0.5 µM purmorphamine (Alexis). Embryoid bodies (EBs) were formed by culturing cells in non-tissue-culture petri dishes (Corning). On day 2 medium was changed to N2B27 medium containing equal parts of neurobasal (Invitrogen) and DMEM-F12 medium (Invitrogen) with 1:100 B27 supplement lacking vitamin A (Invitrogen), 1:200 N2 supplement (Invitrogen), 1% penicillin/streptomycin/glutamine (PSG) and the same small molecules mentioned before. After two additional days, dorsomorphin and SB-431542 were withdrawn, while ascorbic acid (AA; 150 µM) was added to the medium. On day 6, EBs were cut into smaller pieces and plated onto matrigel (Matrigel Growth-factor-reduced, Corning) coated 12-well plates. For passaging, NPCs were treated with accutase. After 3 passages purmorphamine was replaced by 0.5 µM SAG (Cayman Chemical) (NPC medium).

### Immunofluorescence on mouse and human cells

For in vitro analysis, 250,000 of mouse NPCs and iPS-derived human NPCs were plated on MATRIGEL-coated (Becton Dickinson Labware) round 12-mm coverslips in a 24-well plate in 1 ml of Neurocult medium plus proliferation supplement.

NPCs were fixed with 4% PFA after 3 days, at room temperature (RT) for 10 min, then rinsed three times with PBS 1×, and then incubated for 60 min at RT with a blocking solution [PBS 1× + 10% normal goat serum (NGS)]. For intracellular staining, the same blocking solution as above, plus 0.1% Triton X-100, was used. Then fixed cells were incubated for 2 further hours at RT with an appropriate primary antibody diluted in PBS 1X + 1% NGS. Primary antibodies used: rabbit anti mouse Olig2 (Millipore, product no: MABN50, clone: 211F1.1, RRID:AB_10807410 (1:200)), mouse anti mouse Nestin (Millipore, product no: MAB353, clone: rat-40, RRID:AB_94911 (1:100)), rabbit anti mouse Sox2 (Abcam, product no: ab69893, clone: 9-9-3, RRID:AB_1270861 (1:200)), mouse anti human Nestin (Millipore, product no: MAB5326, clone: 10C2, RRID:AB_2251134 (1:200)), mouse anti human IGFBPL1 (Santa Cruz, product no: sc-398875, (1:100)). Cells were then washed three times in 1× PBS and then incubated for 45 min with the appropriate fluorescent secondary antibodies (1:1000, AlexaFluo 488, 546). The nuclei were stained with 4,6 diamine-2-fenilindole (1 μg/ml, DAPI, Roche). Cells were then washed and mounted with Fluorescent mounting medium (Dako).

Light (Olympus, BX51, Japan) and confocal (Leica, SP5 equipped with 40× and 63× objectives, Germany) microscopy images were obtained to analyse cell staining. Image analyses was performed using Adobe Photoshop CC software (Adobe Systems Incorporated) or ImageJ (NIH software).

### Elisa assay

First, we collected plasma and cerebrospinal fluid (CSF), after that  female and male mice were sacrificed under deep anaesthesia with tribromoethanol (Avertin) and transcardially perfused with cold saline solution with EDTA. The brains were rapidly removed after decapitation and the SVZ and striatum were dissected. The samples were homogenized in 200 μl of homogenization buffer (10 mM Tris HCl pH7.4, 260 mM sucrose and cocktail of protease inhibitors) and the homogenate was centrifugated at 1000 × *g* at 4 °C for 10 min to remove nuclei and cell debris.

Total proteins were quantified by using BCA kit (Pierce, Thermo Scientific IL 61101 USA). Samples were normalized for mg of tissue.

ELISA for IGF-1 (DuoSet ELISA kit, R&D Systems, product no: DY791) was performed according to the manufacturer’s instructions.

ELISA for IGFBPL1 was developed in our laboratory. First, we added 100 μl of sample or standards in Reagent Diluent (2% BSA in PBS1×) and incubated overnight at room temperature. As standards we used mIGFBPL1 recombinant protein (R&D Systems, product no: 4130-BL-050); a seven point standard curve using 2-fold serial dilutions in Reagent Diluent, and a high standard 20 ng/ml was performed. Next day, we aspirated each well and washed with Wash Buffer (0.05% Tween in PBS1×) for 3 times (300 μl/well), removing ant remaining Wash Buffer by inverting the plate. Added 100 μl of Primary antibody (goat anti IGFBPL1; R&D Systems, product no: AF4130, RRID:AB_2279980 (1:1000)), diluted in Reagent Diluent and incubated 2 h at room temperature. Repeated the 3 washes with Wash Buffer. Added 100 μl of anti-goat biotinylated secondary antibody (Vector Laboratories, product no: BA-9500 (1:500)) and incubated for 2 h at room temperature. Repeated the 3 washes with Wash Buffer. Added 100 μl of Streptavidin-HRP (R&D Systems) diluted 1:200 in PBS1×. Repeated the 3 washes with Wash Buffer. Added 100 μl of Substrate Solution (R&D Systems, product no: DY999) and incubated for 20 min at room temperature in the dark. Finally, we added 50 μl of Stop Solution (2 N H_2_SO_4_) and determined the optical density, using a microplate reader set (Thermofisher) to 450 nm with a correction of 540 nm.

### Western blot

Mouse NPCs (2 × 10^6^ in T25 flask- line 111107) were plated 24 h before collection. Cells were centrifuged and washed in PBS1×. Then, the pellet was collected in 100 μl of RIPA Buffer (Tris-HCl pH 7.4 50 mM, NaCl 150 mM, Triton 1%, Na Deoxicolate 0.5%, EDTA 1 mM, NaF 10 mM, DTT 1 mM, EGTA 1 mM) and phosphatase and protease inhibitors (Sigma).

Protein content of cell was determined using the BCA protein estimation kit (Pierce, Thermo Scientific IL 61101 USA) and bovine serum albumin as standard.

40 μg of samples proteins were diluted in ddH_2_O and loading buffer 4× and denatured at 100 °C for 5 min. Proteins were separated by SDS (10%)–polyacrylamide gel electrophoresis and transferred to nitrocellulose by electroblotting. Nonspecific binding sites were blocked with TBS- tween 0,1%–5% milk for 1 h and then the blots were incubated overnight at 4 °C in TBS-tween 0.1%-milk 5% with Goat anti-IGFBPL1 (1:250; R&D) and Mouse anti-H3 (1:3000; Immunological Sciences). The immunoreaction was detected by incubating blots for 1 h at room temperature with appropriate peroxidase-labelled secondary antibodies (1:3000) and was visualized with ECL Western Blotting detection reagents (Biorad).

### In vivo local field potentials (LFPs) recordings and analysis

Teflon-insulated stainless-steel wires (∅ 150 μm) were implanted in the right and left striatum and primary sensory cortices (S1). Electrodes were built with a single wire connected to a pin. Under deep sevofluorane anaesthesia, electrodes were stereotaxically implanted in the striatum and S1, according to the following coordinates, in mm: striatum, AP = 0.5, *L* = 1.5, *V* = −2.9; S1, AP = −1.0, *L* = 3.0, *V* = −1.8. A silver wire over the cerebellum was used as reference and ground. All implants were secured using Ketacem cement. After surgery, female mice were allowed to recover for 6–7 days before testing. All recordings were performed inside a customized Faraday chamber and lasted 10 min each, within the home cage. LFPs were recorded and initially digitized at 1 kHz then stored on a hard drive for offline analysis. LFPs epochs were visually examined and power spectra of artefact-free segments were computed using fast Fourier transforms by using the commercial software NeuroExplorer (Plexon). Mean power spectra were divided into frequency bands: 2–7 Hz, 7.01–12 Hz, 12.01–20 Hz, 20.01–30 Hz, 30.01–70 Hz and 70.01–120 Hz^86^. Three epochs of 10 s CS were analysed for each animal and averaged.

Interhemispheric coherence between LFP channels was measured by magnitude squared coherence (MSC), using the function ms cohere in Matlab signal toolbox, which is a coherence estimate of the input signals x and y by using Welch’s averaged, modified periodogram method. The MSC estimate is a function of frequency with values between 0 and 1 and indicates how well x corresponds to y at each frequency. The MSC estimate is calculated over the frequency range of 0.5– 120 Hz for each animal. Statistics were performed on the average difference in coherence within the frequency bands of interest^87^.

### MRI human experiment

Ninety-seven MS patients (mean ± standard deviation [SD] age 36.8 ± 7.56 years, female/male [F/M] = 55/42, median EDSS 2.0 interquartile range [IQR] = 1.0–4.5, median disease duration 5.0 years, IQR = 1–12) and 43 healthy controls (HC, mean ± SD age 34.8 ± 6.52 years, F/M = 19/24) underwent a 3.0 T brain MRI and SDMT testing. MS patients and HC were age- and sex-matched (*p* = 0.11 and *p* = 0.28, respectively). For all participants, exclusion criteria were age more than 50 years, history of drug or alcohol abuse, neurologic disorders (excluding MS in patients), psychiatric comorbidities, history of head trauma and contraindications to MRI. The age restriction was chosen to minimize possible confounding effects of unknown chronic small vessel disorders of the brain. Patients had a diagnosis of clinically isolated syndrome (*n* = 4) or MS (*n* = 93), according to 2017 revision of McDonald criteria^88^. Ethical approval was received from the local ethical standards committee of the San Raffaele Scientific Institute, Milan (Italy), and written informed consent was obtained from all participants at the time of data acquisition. None of the study participants received compensation for participation in the study. The MRI protocol included fluid attenuation inversion recovery (FLAIR) for T2-hyperintense lesion identification, high-resolution 3D T1-weighted sequence for global and regional brain volumes measurement and diffusion weighted images for the assessment of microstructural integrity (fractional anisotropy and mean diffusivity, correlating with axonal and myelin loss respectively). On the same day of MRI, the Symbol Digit Modalities Test (SDMT) was administered to patients. During the test, a nine-symbol code, corresponding to nine digits (from 1 to 9), is presented to the subject. Symbols and corresponding digits are reported on the top of a sheet as a reminder, while the patient is required to decode, based on the key provided, a pseudorandomized sequence of the symbols. The raw score of the test is the number of correct answers provided within 90 s^39^. Hence, an optimal performance requires both a fast information processing speed and a prompt decision making.

The SDMT was normalized according to Italian normative data, as reported^39^. In details, after scores adjustment by subtracting the effects of relevant variables (i.e., education), z-scores were obtained based on the mean and standard deviation of the SDMT performance in the Italian normative population^39^.

#### MRI protocol

Using a 3.0 Tesla Philips Ingenia CX scanner with a dS Head 32-channel receiver coil and standardized procedures for subjects positioning, the following brain MRI protocol was acquired: (1) variable flip angle 3D T2-weighted fluid-attenuated inversion recovery (FLAIR) turbo spin echo (repetition time [TR] = 4800 ms; echo time [TE] = 270 ms; inversion time [TI] = 1650 ms; matrix size = 256 × 256; field of view [FOV] = 256 × 256 mm^2^; echo train length [ETL] = 167; 192 contiguous 1 mm-thick sagittal slices); (2) 3D T1-weighted turbo field echo (TR = 7 ms; TE = 3.2 ms; TI = 1000 ms; flip angle = 8°; matrix size = 256 × 256; FOV = 256 × 256 mm^2^; 204 contiguous 1 mm-thick sagittal slices) and (3) diffusion-weighted pulsed-gradient spin-echo single-shot echo-planar (TR = 5900 ms, TE = 78 ms; matrix size = 112 × 85; FOV = 240 × 233 mm^2^; 56 contiguous 2.3 mm-thick slices; number of excitations [NEX] = 1) with diffusion-weighting (b-factor = 700/1000/2850 s/mm^2^) applied along 6/30/60 noncollinear directions and ten b = 0 volumes distributed along the acquisition, plus three additional b = 0 volumes with reversed polarity of gradients for distortion correction were acquired.

T2-hyperintense brain lesions masks were automatically segmented using the FLAIR and 3D T1-weighted scans as input images^89^ and corresponding volumes were calculated.

After 3D T1-weighted images lesion refilling, head-size-normalized volumes of the entire brain, grey matter, white matter and deep grey matter nuclei (caudate, pallidum, putamen, thalamus and hippocampus) were segmented and measured using FSL SIENAx software and FMRIB’s Integrated Registration and Segmentation Tool (FIRST) pipeline (FMRIB, Oxford, UK) . On the same refilled 3D T1-weighted images, average and lobar (frontal, temporal, parietal, occipital and cingulate) cortical thickness was obtained running the FreeSurfer 6.0 software suite (http://surfer.nmr.mgh.harvard.edu/)^90^.

Pre-processing of diffusion-weighted images included correction for off-resonance and eddy current induced distortions, as well as for movements using the Eddy tool within the FSL library^91^.

Then, the diffusion tensor was estimated from the two lower shells by linear regression^92^ using FSL software (FMRIB, Oxford, UK).

Fractional anisotropy and mean diffusivity maps were derived and measured within lesions and in the normal appearing tissue, defined as those voxels not involved by T2-hyperintense lesions.

A SVZ mask was segmented on 3D T1-weighted images in the standard space (Montreal Neurological Institute space) according to anatomical references based on lateral ventricles and caudate segmentation^38^.

SVZ and T2-hyperintense lesion masks were registered on native 3D T1-weighted and diffusion weighted images to obtain the percentage of lesioned SVZ (SVZ percentage lesion volume) and intralesional/normal appearing tissue measures of microstructural integrity.

Age-, sex- and phenotypes-adjusted partial correlations and stepwise multiple linear regression models (selection method=stepwise) were run to identify independent predictors of caudate volume and SDMT z-scores in MS patients. Schwarz information criterion (SBC) was used for both inclusion/exclusion criterion on independent variables previously included for partial correlations estimation.

The proportion of variance explained by each model was expressed by the *R*^2^ index and standardized estimates and t values were reported to compare the relative strengths of association of each predictor with the dependent variable.

For partial correlations and stepwise multiple linear regressions, the following measures of SVZ and brain damage were included: SVZ percentage lesion volume, fractional anisotropy in the normal-appearing tissue of SVZ, intralesional fractional anisotropy in the SVZ, mean diffusivity in the normal-appearing tissue of SVZ, intralesional mean diffusivity in the SVZ, brain volume, grey matter volume, white matter volume, logarithm of T2-hyperintense lesion volume, deep grey matter volume (excluding the caudate), caudate volume, white matter fractional anisotropy, white matter mean diffusivity, and mean cortical thickness. The variables included in each analysis are specified in corresponding supplementary Tables.

### Statistical analysis

For data collection and analysis we used Microsoft Excel 2011, version 14.7.2.

For statistical analyses, we used a standard software package (GraphPad Prism version 7.00). Data were evaluated by unpaired two-tailed *t*-test (for comparisons between two groups) or by one-way ANOVA followed by post hoc analysis (for comparison among three groups) as indicated in the figure legends. The significance level was established at *p* = 0.05. A post hoc analysis was performed using Bonferroni correction. For local field potential experiments Kruskal–Wallis was used followed by Dunn’s correction. For patch clamp experiments we used unpaired two-tailed *t*-tests or mann–Whitney *U* tests, one-way ANOVA followed by Tukey post-test and *Z*-test for two population proportions. For behavioural experiments, four-way nonparametric ANOVA (see Supplementary Material) was used to compare the frequency of nose pokes during the presentation of the two different CSs, between the two genotypes, in successive days. On the day four, the time courses of the poking frequencies were compared by transforming them into cumulative probability distributions (pokes vs. time) to contrast the data from the two genotypes using the Kolmogorov-Smirnov test.

### Reporting summary

Further information on research design is available in the Nature Portfolio Reporting Summary linked to this article.

## Supplementary information

Supplementary Information

Reporting Summary

## Data Availability

A reporting summary for this article is available as Supplementary Information file. The main data supporting the findings of this study are available within the article and its Supplementary files. Additional details on datasets and protocols that support the findings of this study will be made available by the corresponding author upon reasonable request. Source data are provided with this paper. RNAseq data are deposited and the accession number is GSE165815. hg38 reference genome is: arXiv:1303.3997 (q-bio.GN). For SVZ single cell RNAseq from Mizrak et al.^22^ ref. GEO accession number GSE109447; for striatum single cell RNAseq from Munoz-Manchado et al.^30^ ref. GEO accession number GSE97478. Human single cell and data ATAC-seq: previously published single cell data for iPSC and NPC^93^ are available from ArrayExpress (https://www.ebi.ac.uk/biostudies/arrayexpress) with the following accession numbers: E-MTAB-10220 for scRNA-seq and E-MTAB-10218 for chromatin accessibility. Source data are provided with this paper.

## Source data

Source Data

## References

[1] Doetsch F, Caille I, Lim DA, Garcia-Verdugo JM, Alvarez-Buylla A (1999). Subventricular zone astrocytes are neural stem cells in the adult mammalian brain. Cell.

[2] Nott A (2019). Brain cell type-specific enhancer-promoter interactome maps and disease-risk association. Science.

[3] Sanai N (2004). Unique astrocyte ribbon in adult human brain contains neural stem cells but lacks chain migration. Nature.

[4] Ernst A, Frisen J (2015). Adult neurogenesis in humans- common and unique traits in mammals. PLoS Biol..

[5] Bacigaluppi M, Sferruzza G, Butti E, Ottoboni L, Martino G (2020). Endogenous neural precursor cells in health and disease. Brain Res..

[6] Spalding KL (2013). Dynamics of hippocampal neurogenesis in adult humans. Cell.

[7] Inta D (2008). Neurogenesis and widespread forebrain migration of distinct GABAergic neurons from the postnatal subventricular zone. Proc. Natl Acad. Sci. USA.

[8] Martino G, Bacigaluppi M, Peruzzotti-Jametti L (2011). Therapeutic stem cell plasticity orchestrates tissue plasticity. Brain.

[9] Lu Z (2011). Phagocytic activity of neuronal progenitors regulates adult neurogenesis. Nat. Cell Biol..

[10] Mosher KI (2012). Neural progenitor cells regulate microglia functions and activity. Nat. Neurosci..

[11] Mohammad MG (2014). Immune cell trafficking from the brain maintains CNS immune tolerance. J. Clin. Investig..

[12] Pluchino S (2008). Persistent inflammation alters the function of the endogenous brain stem cell compartment. Brain.

[13] Butti E (2019). Neural stem cells of the subventricular zone contribute to neuroprotection of the Corpus Callosum after cuprizone-induced demyelination. J. Neurosci..

[14] Tavazoie M (2008). A specialized vascular niche for adult neural stem cells. Cell Stem Cell.

[15] Lee K (2017). Parvalbumin interneurons modulate striatal output and enhance performance during associative learning. Neuron.

[16] Tepper JM (2018). Heterogeneity and diversity of striatal GABAergic interneurons: update 2018. Front. Neuroanat..

[17] Taverna S, Ilijic E, Surmeier DJ (2008). Recurrent collateral connections of striatal medium spiny neurons are disrupted in models of Parkinson’s disease. J. Neurosci..

[18] Straub C (2016). Principles of synaptic organization of GABAergic interneurons in the striatum. Neuron.

[19] Cox J, Witten IB (2019). Striatal circuits for reward learning and decision-making. Nat. Rev. Neurosci..

[20] Friedman A (2017). Chronic STress Alters Striosome-circuit Dynamics, Leading to Aberrant Decision-making. Cell.

[21] Guo C (2018). IGFBPL1 regulates axon growth through IGF-1-mediated signaling cascades. Sci. Rep..

[22] Mizrak D (2019). Single-cell analysis of regional differences in adult V-SVZ neural stem cell lineages. Cell Rep..

[23] Butti E (2012). Subventricular zone neural progenitors protect striatal neurons from glutamatergic excitotoxicity. Brain.

[24] Liu X, Wang Q, Haydar TF, Bordey A (2005). Nonsynaptic GABA signaling in postnatal subventricular zone controls proliferation of GFAP-expressing progenitors. Nat. Neurosci..

[25] Jan YN, Jan LY (2010). Branching out: mechanisms of dendritic arborization. Nat. Rev. Neurosci..

[26] Sholl DA (1953). Dendritic organization in the neurons of the visual and motor cortices of the cat. J. Anat..

[27] More L (2017). Altered fronto-striatal functions in the Gdi1-null mouse model of X-linked intellectual disability. Neuroscience.

[28] Tremblay R, Lee S, Rudy B (2016). GABAergic interneurons in the neocortex: from cellular properties to circuits. Neuron.

[29] Koos T, Tepper JM (1999). Inhibitory control of neostriatal projection neurons by GABAergic interneurons. Nat. Neurosci..

[30] Munoz-Manchado AB (2018). Diversity of interneurons in the dorsal striatum revealed by single-cell RNA sequencing and PatchSeq. Cell Rep..

[31] Stuart, T. S. A., Lareau, C. & Satija, R. Multimodal single-cell chromatin analysis with Signac. *bioRxiv*, (2020).

[32] Meuleman W (2020). Index and biological spectrum of human DNase I hypersensitive sites. Nature.

[33] Amato MP, Zipoli V, Portaccio E (2006). Multiple sclerosis-related cognitive changes: a review of cross-sectional and longitudinal studies. J. Neurol. Sci..

[34] Darby RR, Dickerson BC (2017). Dementia, decision making, and capacity. Harv. Rev. Psychiatry.

[35] Salinas E, Scerra VE, Hauser CK, Costello MG, Stanford TR (2014). Decoupling speed and accuracy in an urgent decision-making task reveals multiple contributions to their trade-off. Front. Neurosci..

[36] Benedict RH (2017). Validity of the Symbol Digit Modalities Test as a cognition performance outcome measure for multiple sclerosis. Mult. Scler..

[37] Uitdehaag BMJ (2018). Disability outcome measures in phase III clinical trials in multiple sclerosis. CNS Drugs.

[38] Cherubini A (2010). A multimodal MRI investigation of the subventricular zone in mild cognitive impairment and Alzheimer’s disease patients. Neurosci. Lett..

[39] Amato MP (2006). The Rao’s Brief Repeatable Battery and Stroop Test: normative values with age, education and gender corrections in an Italian population. Mult. Scler..

[40] Imayoshi I (2008). Roles of continuous neurogenesis in the structural and functional integrity of the adult forebrain. Nat. Neurosci..

[41] Kokaia Z, Martino G, Schwartz M, Lindvall O (2012). Cross-talk between neural stem cells and immune cells: the key to better brain repair?. Nat. Neurosci..

[42] Martino G, Butti E, Bacigaluppi M (2014). Neurogenesis or non-neurogenesis: that is the question. J. Clin. Investig..

[43] Shen W, Da Silva JS, He H, Cline HT (2009). Type A GABA-receptor-dependent synaptic transmission sculpts dendritic arbor structure in Xenopus tadpoles in vivo. J. Neurosci..

[44] Leitch E, Coaker J, Young C, Mehta V, Sernagor E (2005). GABA type-A activity controls its own developmental polarity switch in the maturing retina. J. Neurosci..

[45] Engel AK, Fries P, Singer W (2001). Dynamic predictions: oscillations and synchrony in top-down processing. Nat. Rev. Neurosci..

[46] Varela F, Lachaux JP, Rodriguez E, Martinerie J (2001). The brainweb: phase synchronization and large-scale integration. Nat. Rev. Neurosci..

[47] Buzsaki G, Draguhn A (2004). Neuronal oscillations in cortical networks. Science.

[48] Plenz D (2003). When inhibition goes incognito: feedback interaction between spiny projection neurons in striatal function. Trends Neurosci..

[49] Gage GJ, Stoetzner CR, Wiltschko AB, Berke JD (2010). Selective activation of striatal fast-spiking interneurons during choice execution. Neuron.

[50] Tecuapetla F, Jin X, Lima SQ, Costa RM (2016). Complementary contributions of striatal projection pathways to action initiation and execution. Cell.

[51] Pisansky MT (2019). Nucleus accumbens fast-spiking interneurons constrain impulsive action. Biol. Psychiatry.

[52] Car H, Wisniewski K (1998). Similarities and interactions between GABAergic and glutaminergic systems. Roczniki Akademii Medycznej w Bialymstoku.

[53] Lenz JD, Lobo MK (2013). Optogenetic insights into striatal function and behavior. Behav. Brain Res..

[54] Bragado Alonso, S. et al. An increase in neural stem cells and olfactory bulb adult neurogenesis improves discrimination of highly similar odorants. *EMBO J* **38**, (2019). 10.15252/embj.201798791 PMC6418468 30643018

[55] Nieto-Estevez V, Defterali C, Vicario-Abejon C (2016). IGF-I: a key growth factor that regulates neurogenesis and synaptogenesis from embryonic to adult stages of the brain. Front. Neurosci..

[56] Baruch K (2014). Aging. Aging-induced type I interferon response at the choroid plexus negatively affects brain function. Science.

[57] Lewitt MS, Boyd GW (2019). The role of insulin-like growth factors and insulin-like growth factor-binding proteins in the nervous system. Biochem. Insights.

[58] Ni W, Rajkumar K, Nagy JI, Murphy LJ (1997). Impaired brain development and reduced astrocyte response to injury in transgenic mice expressing IGF binding protein-1. Brain Res..

[59] Martino G, Pluchino S (2006). The therapeutic potential of neural stem cells. Nat. Rev. Neurosci..

[60] Carro E, Spuch C, Trejo JL, Antequera D, Torres-Aleman I (2005). Choroid plexus megalin is involved in neuroprotection by serum insulin-like growth factor I. J. Neurosci..

[61] Liu Z (2015). Magnetization transfer ratio measures in normal-appearing white matter show periventricular gradient abnormalities in multiple sclerosis. Brain.

[62] Pardini M (2019). CSF oligoclonal bands and normal appearing white matter periventricular damage in patients with clinically isolated syndrome suggestive of MS. Mult. Scler. Relat. Disord..

[63] Fadda G (2019). A surface-in gradient of thalamic damage evolves in pediatric multiple sclerosis. Ann. Neurol..

[64] Amato MP (2010). Cognitive impairment in early stages of multiple sclerosis. Neurological Sci..

[65] Gveric D, Cuzner ML, Newcombe J (1999). Insulin-like growth factors and binding proteins in multiple sclerosis plaques. Neuropathol. Appl. Neurobiol..

[66] Cui QL (2012). Human fetal oligodendrocyte progenitor cells from different gestational stages exhibit substantially different potential to myelinate. Stem Cells Dev..

[67] Harrington DL (2014). Neuroanatomical correlates of cognitive functioning in prodromal Huntington disease. Brain Behav..

[68] Unmack Larsen I, Vinther-Jensen T, Gade A, Nielsen JE, Vogel A (2015). Assessing impairment of executive function and psychomotor speed in premanifest and manifest Huntington’s disease gene-expansion carriers. J. Int Neuropsychol. Soc..

[69] Unified Huntington’s Disease Rating Scale: reliability and consistency. Huntington Study Group. (1996). Mov. Disord..

[70] Hansch EC (1982). Cognition in Parkinson disease: an event-related potential perspective. Ann. Neurol..

[71] Bayram E, Kaplan N, Shan G, Caldwell JZK (2020). The longitudinal associations between cognition, mood and striatal dopaminergic binding in Parkinson’s Disease. Neuropsychol. Dev. Cogn. B Aging Neuropsychol. Cogn..

[72] Corada M (2019). Fine-tuning of Sox17 and canonical Wnt coordinates the permeability properties of the blood-brain barrier. Circ. Res..

[73] Pluchino S (2003). Injection of adult neurospheres induces recovery in a chronic model of multiple sclerosis. Nature.

[74] Schneggenburger R, Meyer AC, Neher E (1999). Released fraction and total size of a pool of immediately available transmitter quanta at a calyx synapse. Neuron.

[75] Ferro M (2017). Functional mapping of brain synapses by the enriching activity-marker SynaptoZip. Nat. Commun..

[76] Ritchie ME (2015). limma powers differential expression analyses for RNA-sequencing and microarray studies. Nucleic Acids Res..

[77] Subramanian A (2005). Gene set enrichment analysis: a knowledge-based approach for interpreting genome-wide expression profiles. Proc. Natl Acad. Sci. USA.

[78] Smith T, Heger A, Sudbery I (2017). UMI-tools: modeling sequencing errors in unique molecular identifiers to improve quantification accuracy. Genome Res..

[79] Harrow J (2012). GENCODE: the reference human genome annotation for The ENCODE Project. Genome Res..

[80] Wolf FA, Angerer P, Theis FJ (2018). SCANPY: large-scale single-cell gene expression data analysis. Genome Biol..

[81] Lopez-Delisle, L. et al. pyGenomeTracks: reproducible plots for multivariate genomic data sets. *Bioinformatics*, (2020). 10.1093/bioinformatics/btaa692 PMC8058774 32745185

[82] Wolock SL, Lopez R, Klein AM (2019). Scrublet: computational identification of cell doublets in single-cell transcriptomic data. Cell Syst..

[83] Korsunsky I (2019). Fast, sensitive and accurate integration of single-cell data with Harmony. Nat. Methods.

[84] Centonze D (2007). The endocannabinoid system is dysregulated in multiple sclerosis and in experimental autoimmune encephalomyelitis. Brain.

[85] Muzio, L. et al. Cxcl10 enhances blood cells migration in the sub-ventricular zone of mice affected by experimental autoimmune encephalomyelitis. *Mol Cell Neurosci*, (2009). 10.1016/j.mcn.2009.11.008 19969087

[86] Cambiaghi M, Magri L, Cursi M (2015). Importance of EEG in validating the chronic effects of drugs: suggestions from animal models of epilepsy treated with rapamycin. Seizure.

[87] Cambiaghi M (2016). Higher-order sensory cortex drives basolateral amygdala activity during the recall of remote, but not recently learned fearful memories. J. Neurosci..

[88] Thompson AJ (2018). Diagnosis of multiple sclerosis: 2017 revisions of the McDonald criteria. Lancet Neurol..

[89] Valverde S (2017). Improving automated multiple sclerosis lesion segmentation with a cascaded 3D convolutional neural network approach. NeuroImage.

[90] Fischl B, Dale AM (2000). Measuring the thickness of the human cerebral cortex from magnetic resonance images. Proc. Natl Acad. Sci. USA.

[91] Andersson JLR (2017). Towards a comprehensive framework for movement and distortion correction of diffusion MR images: within volume movement. NeuroImage.

[92] Basser PJMJ, LeBihan D (1994). MR diffusion tensor spectroscopy and imaging. Biophys. J..

[93] Tedesco M (2022). Chromatin velocity reveals epigenetic dynamics by single-cell profiling of heterochromatin and euchromatin. Nat. Biotechnol..

