# Supplementary material for: Neural precursor cells tune striatal connectivity through the release of IGFBPL1
Source: Nat Commun. 2022 Dec 8;13:7579. doi: 10.1038/s41467-022-35341-y (PMC9731988; doi:10.1038/s41467-022-35341-y)

Supplementary figures:

Supplementary Fig.1

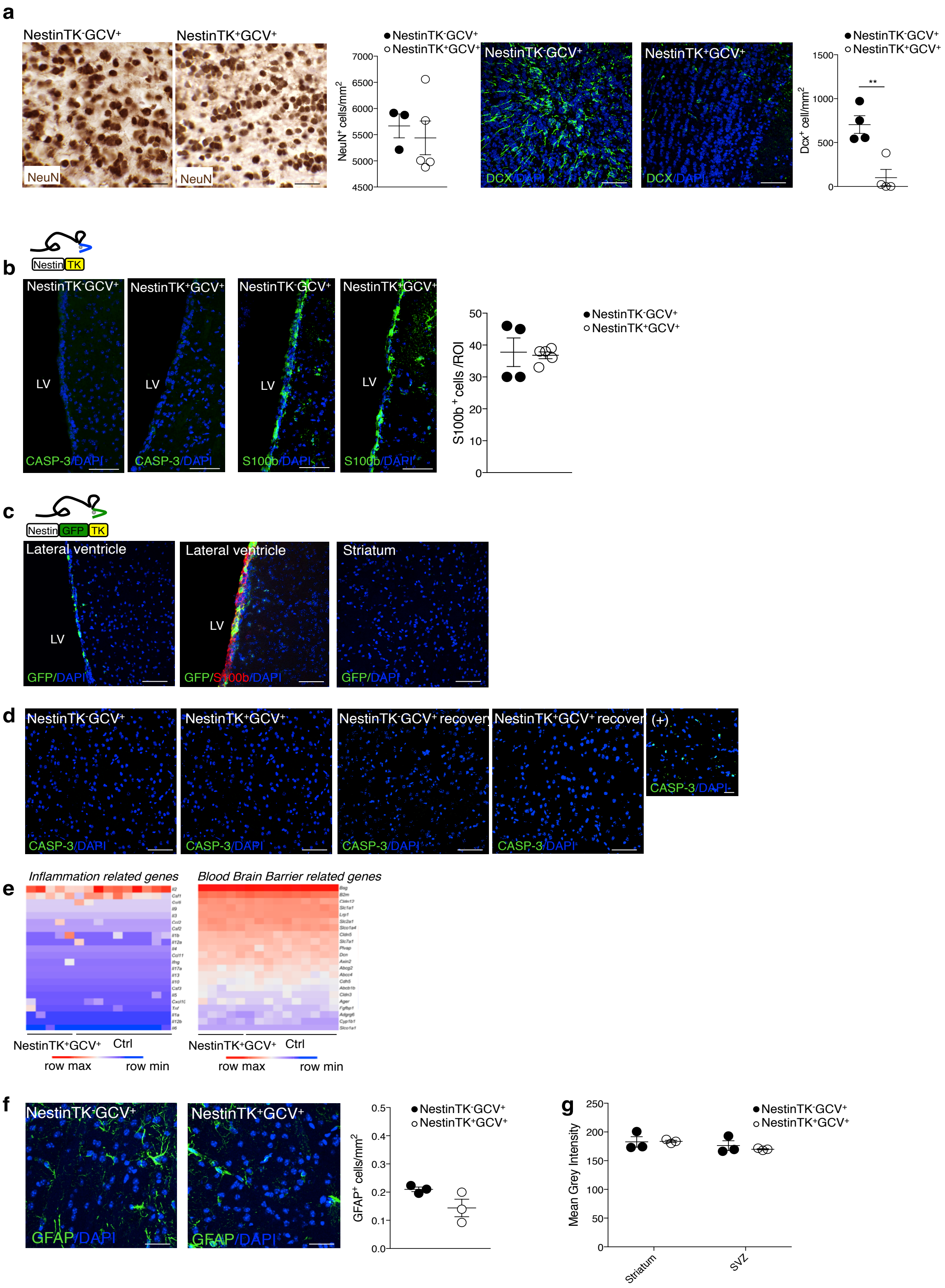

Supplementary Fig.1 Study of olfactory bulbs, ependymal cells, neurons, apoptosis, astrocytes, inflammation and blood-brain barrier integrity in eNPCs ablated mice.

a Representative images and quantification of neurons ($NeuN^+$ in brown) in the olfactory bulbs (OB) of $NestinTK^-GCV^+$ and $NestinTK^+GCV^+$ mice. n=3 $NestinTK^-GCV^+$ and n=5 $NestinTK^+GCV^+$. On the right representative confocal images and quantification of DCX^+ cells (in green) in the OB of $NestinTK^-GCV^+$ and $NestinTK^+GCV^+$ mice. Nuclei stained by DAPI in blue. Scale bar: 50 µm. Values represent mean ± SEM. In a **p= 0.0046. Unpaired two tailed t-test. n= 4 mice per group. **b** Representative confocal images of the lateral ventricle of $NestinTK^-GCV^+$ and $NestinTK^+GCV^+$ mice stained for ependymal cells (S100β) or apoptotic cells (activated caspase-3) in green. Nuclei stained by DAPI in blue. Scale bar: 50 µm. Values represent mean ± SEM. n= 3 mice per group. **c** Representative confocal images of NestinGFPTK. Slides labelled for GFP (in green) and S100β (in red). Nuclei stained by DAPI in blue. Scale bar: 50 µm. **d** Representative images of the immunofluorescence staining for activated Caspase-3 (in green) in the striatum of $NestinTK^-GCV^+$ and $NestinTK^+GCV^+$ mice at the end of GCV administration and during the recovery phase. On the right a positive control (+) for activated caspase-3 staining is shown (ischemic striatal tissue section). Nuclei in blue counterstained by DAPI. Scale bar: 50 µm. **e** Heatmaps showing the gene expression values for inflammatory genes and genes involved in the blood- brain-barrier integrity in control and $NestinTK^+GCV^+$ mice. (blue means downregulation, red upregulation) n=5 mice per group. **f** Representative images and quantification of astrocytes in the striatum of $NestinTK^-GCV^+$ and $NestinTK^+GCV^+$ mice: astrocytes labelled for GFAP (in green). Nuclei in blue counterstained by DAPI. Scale bar: 50 µm. Values represent mean ± SEM. n= 3 mice per group. **g** Quantification of the IgG leakage in $NestinTK^-GCV^+$ and $NestinTK^+GCV^+$ mice at the level of the SVZ and of the striatum. A value of 0 corresponds to black, a value of 255 to white (a higher number denotes low IgG extravasation). Values represent mean ± SEM. n= 3 mice per group. Source data are provided as a Source Data file.

Supplementary Fig.2

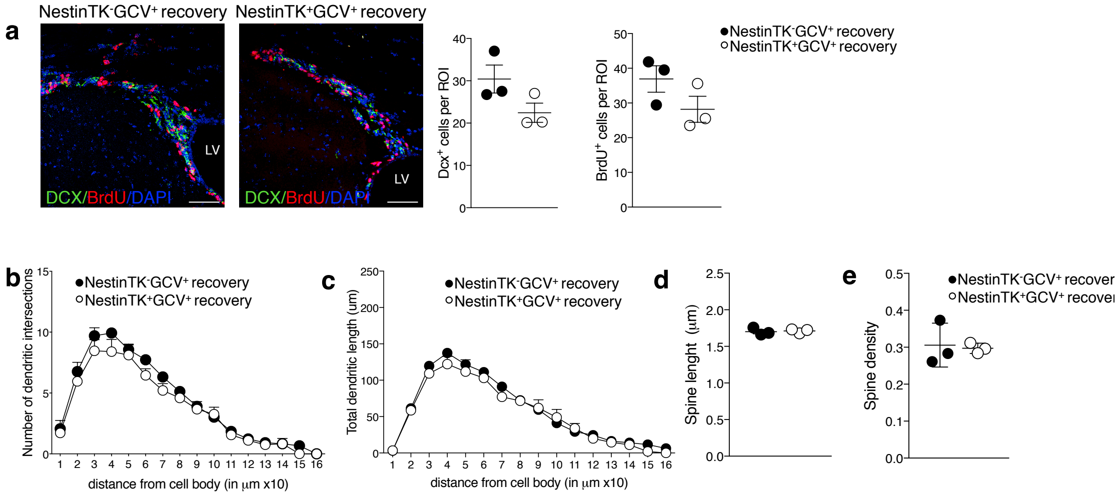

Supplementary Fig.2 Neurogenesis, striatal MSN morphology are rescued in SVZ-eNPC ablated mice

a Representative images and quantification of eNPC in the SVZ one month after the end of GCV treatment (recovery phase) in NestinTK^{-}GCV^{+} and NestinTK^{+}GCV^{+} mice: neuroblasts were labelled for doublecortin (DCX, in green) and transient amplifying cells for BrdU (in red). Nuclei in blue were counterstained by DAPI. The lateral ventricle is denoted as LV. Scale bar: 50 µm. n= 3 mice per group. **b and c** Sholl analysis comparing tracings of medium spiny neurons from NestinTK^{-}GCV^{+} and NestinTK^{+}GCV^{+} in the recovery phase; n=6-8 neurons per mice for a total of 3 mice per group. Data are presented as number of dendritic intersections **(b)**, and total dendritic length plotted at increasing distance from the cell body **(c).** Values represent the mean ± SEM. **d and e** Striatal MSNs spine length **(d)**, and spine density **(e)** in NestinTK^{-}GCV^{+} compared to NestinTK^{+}GCV^{+} mice in the recovery phase. Values represent the mean ± SEM. n= 3 mice per group. Source data are provided as a Source Data file.

Supplementary Fig.3

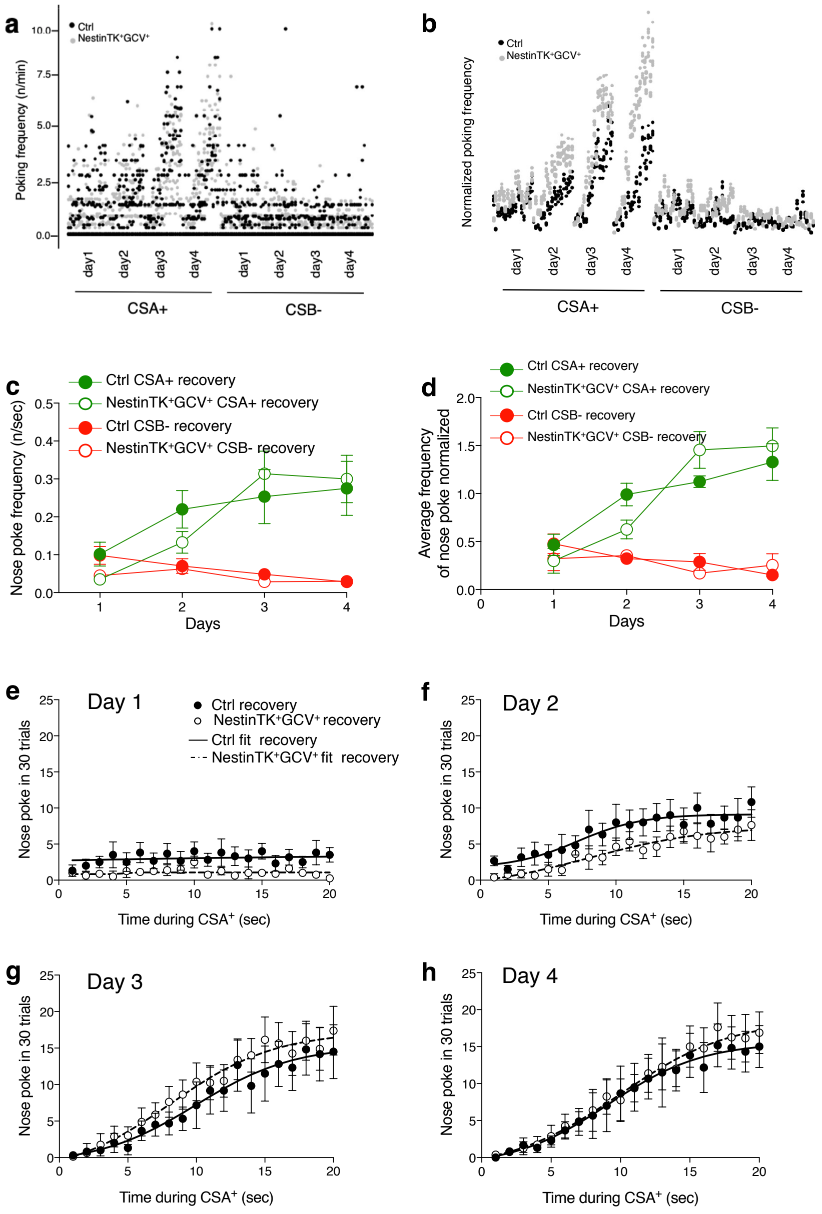

Supplementary Fig.3 Behavioural analysis in control and ablated mice

a Raw data of nose poking frequency in the subsequent 4 days during the 20 s duration of CSA+ or CSB- stimuli for each NestinTK$^-$GCV$^+$ (Control mice, Ctrl) and NestinTK$^+$GCV$^+$ mice at the end of GCV administration. **b** Same data as in (**a**), scaled for each mouse based on the ratio between its own average poking frequency and the average frequency for all mice of the same genotype (more precisely, on the regression slope). Notice that the learning curve appears to be quite similar among

animals, and similar between the two genotypes as well: the main difference between the genotypes seems to consist in a scaling factor, ablated mice displaying a general tendency to poke more under all conditions. **c-d** The graphs show the average of nose poking frequency during the 20 s CS (A^+ or B^-) tone during the 4 days of test in $NestinTK^-GCV^+$ (Control mice, Ctrl) and $NestinTK^+GCV^+$ one month after the end of GCV administration (recovery phase, independent cohort of mice) (**c**) and the nose poking frequencies after data normalization (**d**). **e-h** Time course of nose poking during the CSA+ in the subsequent days of the experiments in Ctrl and $NestinTK^+GCV^+$ one month after the end of GCV administration. The total number of pokes in the 30 trials is displayed for every second during the CS. No differences between control and ablated mice. n = 8 $NestinTK^+GCV^+$ male mice and n = 6 Ctrl male mice. Values represent the mean ± SEM. Source data are provided as a Source Data file.

Supplementary Fig.4

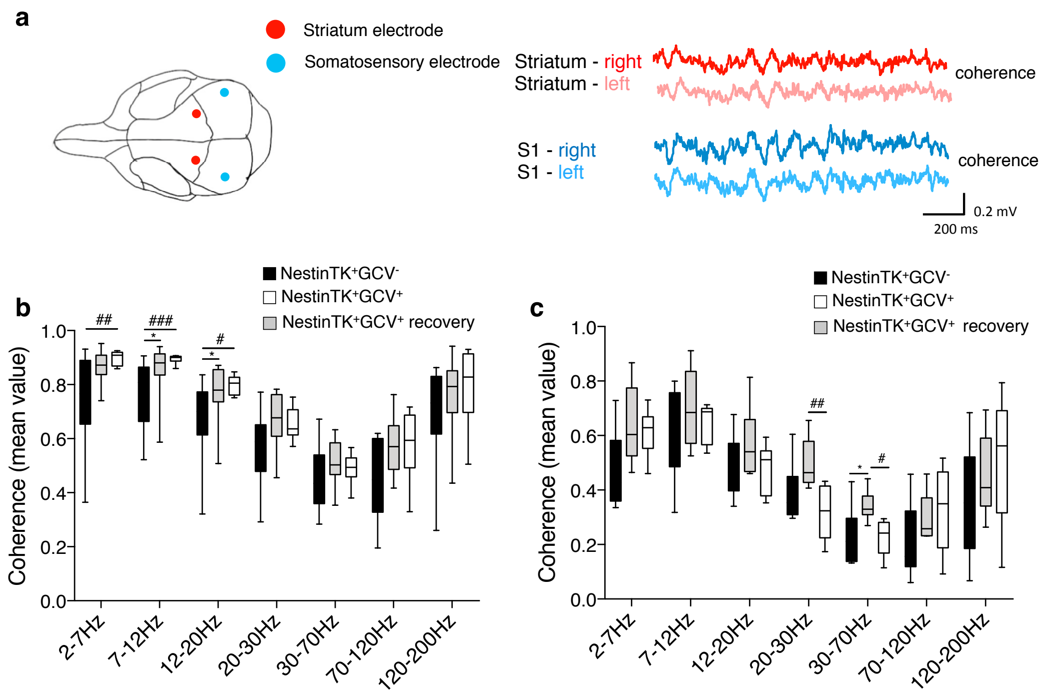

Supplementary Fig.4 The SVZ ablation causes persistent increase in low-frequencies coherence between the right and left striatum

a Schematic representation of the implanted electrodes (striata in red and somatosensory cortices in blue) and interhemispheric coherence analysis of LFP signal, showing higher striatum-striatum synchrony but not S1-S1. **b and c** The interhemispheric coherence in the NestinTK^{+} mice before the treatment with GCV (NestinTK^{+}GCV^{-}), after 4 weeks of GCV treatment (NestinTK^{+}GCV^{+}) and one month after the end of GCV administration (NestinTK^{+}GCV^{+} recovery) from 2 to 200 Hz between right and left striatum (n = 14 for NestinTK^{+}GCV^{-}, n=13 for NestinTK^{+}GCV^{+} and n=9 NestinTK^{+}GCV^{+} in the recovery phase) in **b** and between the right and left somato-sensorial cortex (n = 6 for NestinTK^{+}GCV^{-}, n=6 for NestinTK^{+}GCV^{+} and n=9 NestinTK^{+}GCV^{+} in the recovery phase) in **c**. Values represent means ± SEM. The box extends from the 25th to 75th percentiles, the midline denotes the median while whiskers plot min and max. In **b**, for 2-7 Hz ## p= 0.0086, for 7-12 Hz *p=0.0281, ###p=0.0039, for 12-20 Hz *p=0.0358, #p=0.0144; in **c**, for 20-30 Hz

##p=0.0084, for 30-70 Hz *p=0.0315, #p=0.0341. Unpaired Kruskal-Wallis test followed by Dunn's correction was used. Source data are provided as a Source Data file.

Supplementary Fig.5

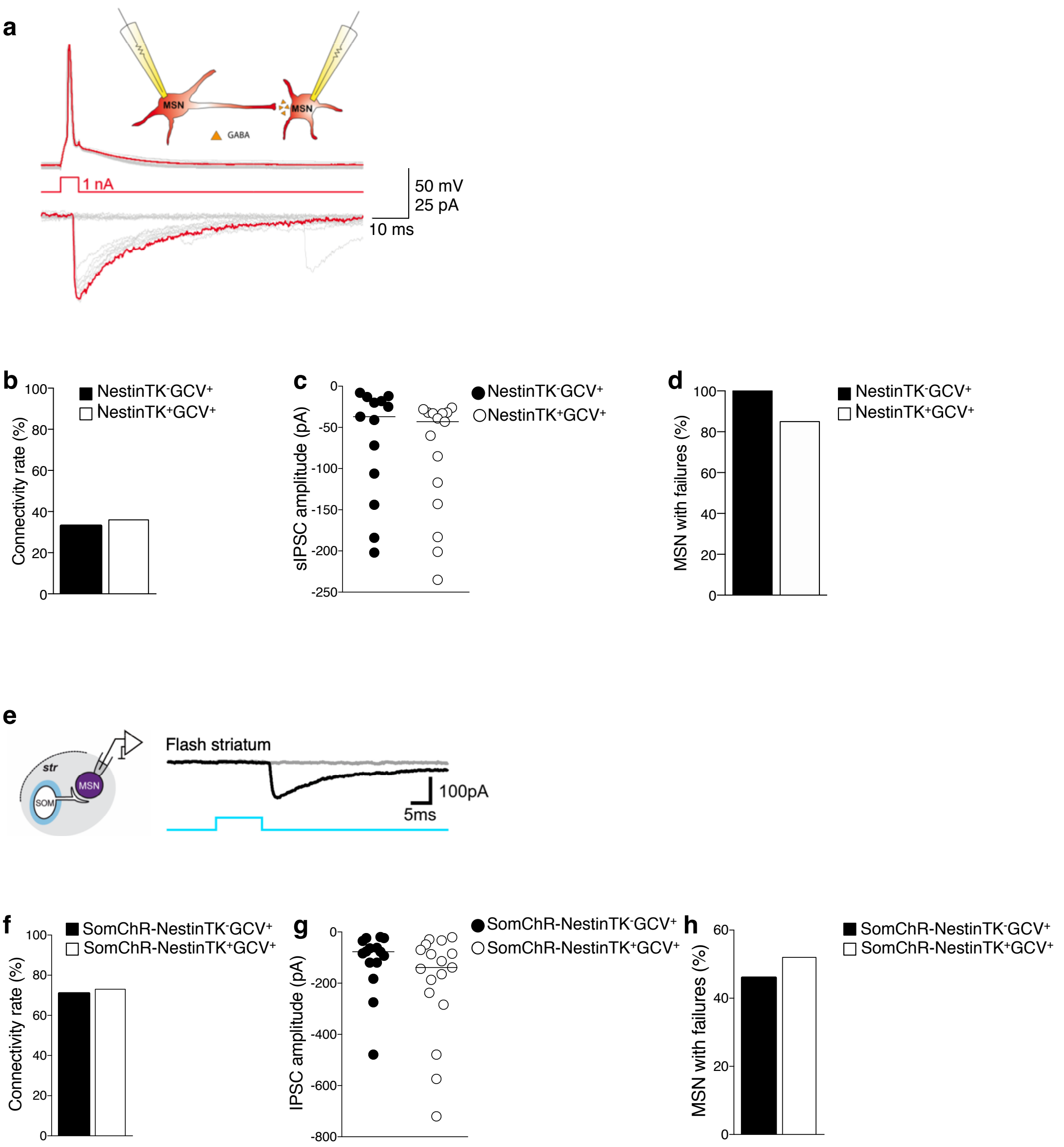

Supplementary Fig.5 Inhibitory GABAergic transmission in striatal MSN pairs and GABAergic transmission mediated by somatostatin (SOM)-expressing interneurons were not impaired after NPC ablation.

a Examples of unitary IPSCs evoked in an MSN in response to individual APs elicited in a synaptically connected MSN. Both successful IPSCs and failures are shown. The drawing represents the recording configuration. **b** Connectivity rates for MSN-MSN pairs in NestinTK^{-}

GCV^+ and $NestinTK^+GCV^+$ slices. n=5 mice per group. **c** Summary of average amplitudes of unitary IPSCs in MSN-MSN pairs from $NestinTK^-GCV^+$ (n =11 cells in 5 mice) and $NestinTK^+GCV^+$ (n =11 cells in 5 mice) slices. Values represent medians. **d** Summary histogram comparing reliability of MSN-MSN GABAergic synapses (measured as % of MSNs displaying at least one failure). n=5 mice per group. **e** Left, schematic cartoon showing the experimental approach used to optogenetically stimulate striatal SOM interneurons. Right, example of a unitary IPSC and a failure evoked in a MSN in response to optical activation (represented by the blue trace on bottom) of striatal SOM interneurons. **f** Summaries of connectivity rates of MSNs. n=3 mice for $SomChR\text{-}NestinTK^-GCV^+$ and n=5 mice for $SomChR\text{-}NestinTK^+GCV^+$. **g and h** Summary plots with unitary parameters (peak amplitude and failure rates) for SOM interneuron-mediated IPSCs in $SomChR\text{-}NestinTK^-GCV^+$ (n=3) and $SomChR\text{-}NestinTK^+GCV^+$ mice (n=5). Summaries of percentages of MSNs showing failures in response to optogenetic stimulation of SOM interneurons 15 out of 22 (68%) and 19 out of 27 (70%) MSNs were found connected with at least one SOM interneuron in $SomChR\text{-}NestinTK^-GCV^+$ (n=3 mice) and $SomChR\text{-}NestinTK^+GCV^+$ (n=5 mice) slices respectively. Of these, 46% (7/15) and 53% (10/19) displayed at least one failure. Source data are provided as a Source Data file.

Supplementary Fig.6

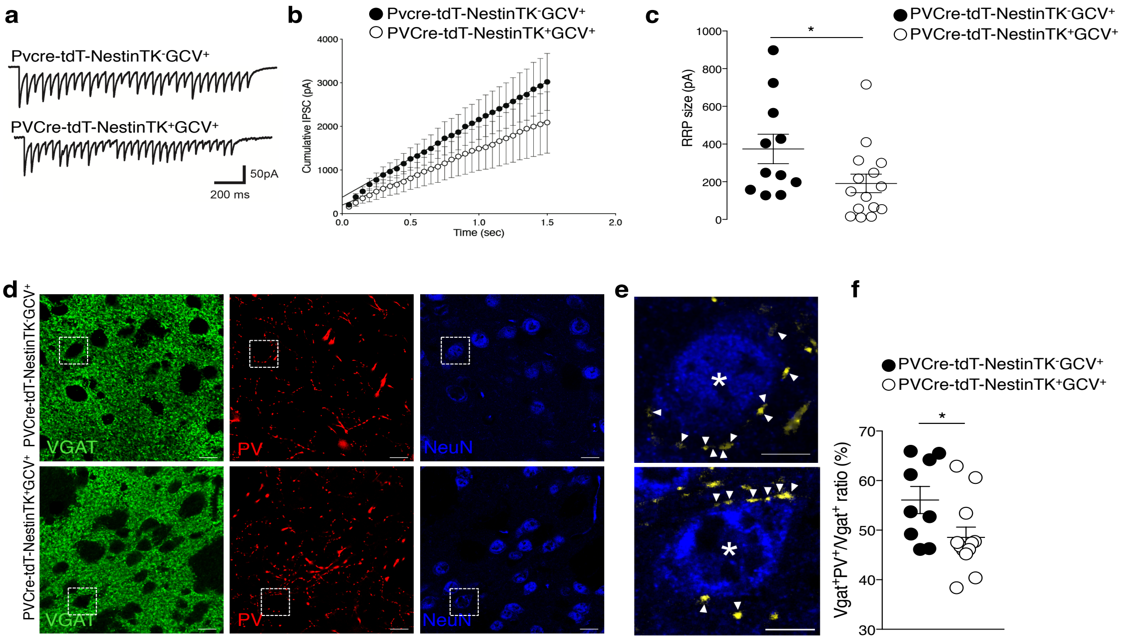

Supplementary Fig.6 SVZ-eNPC ablation causes presynaptic defects in striatal GABAergic terminals

a Examples of IPSC trains evoked in MSNs in response to extracellular, high-frequency local stimulation (50 Hz, 1s). **b** Cumulative plots of IPSC peak amplitudes recorded in MSNs from PVcre-tdT-NestinTK$^-$GCV$^+$ and PVcre-tdT-NestinTK$^+$GCV$^+$ mice. The linear portion of the cumulative IPSC amplitude distribution was fit with a straight line that was prolonged to intercept the Y-axis, in order to obtain an estimated value of the vesicular readily releasable pool (RRP). **c** Summary plot of RRP sizes in MSNs from PVcre-tdT-NestinTK$^-$GCV$^+$ (n=11 pairs in 8 mice) and PVcre-tdT-NestinTK$^+$GCV$^+$ (n=15 pairs in 12 mice). Values represent mean ± SEM. Unpaired two-tailed t-test *p=0.048. **d** Representative confocal microscopy images showing striatal coronal brain sections in PVcre-tdT-NestinTK$^-$GCV$^+$ and PVcre-tdT-NestinTK$^+$GCV$^+$ mice. Scale bar: 10 µm. **e** Magnification of the areas indicated by the squares in **d**, representing ImageJ "math plug-in" reconstructions highlighting double positive (vGAT+/PV+) perisomatic puncta (marked by

arrowheads). Asterisks indicate $NeuN^+$ somata. Scale bar: 5 µm. **f** Quantification of presynaptic terminals expressing both PV and vGAT, normalized over the total vGAT positive perisomatic synapses in PVcre-tdT-$NestinTK^-GCV^+$ and PVcre-tdT-$NestinTK^+GCV^+$ mice. Values represent mean ± SEM. *p=0.0379; Mann-Whitney U test two-tailed (56.1% ± 2.7, n=9 for PVcre-tdT-$NestinTK^-GCV^+$ 48.5% ± 2.1, n=12 for PVcre-tdT-$NestinTK^+GCV^+$ mice. Source data are provided as a Source Data file.

Supplementary Fig.7

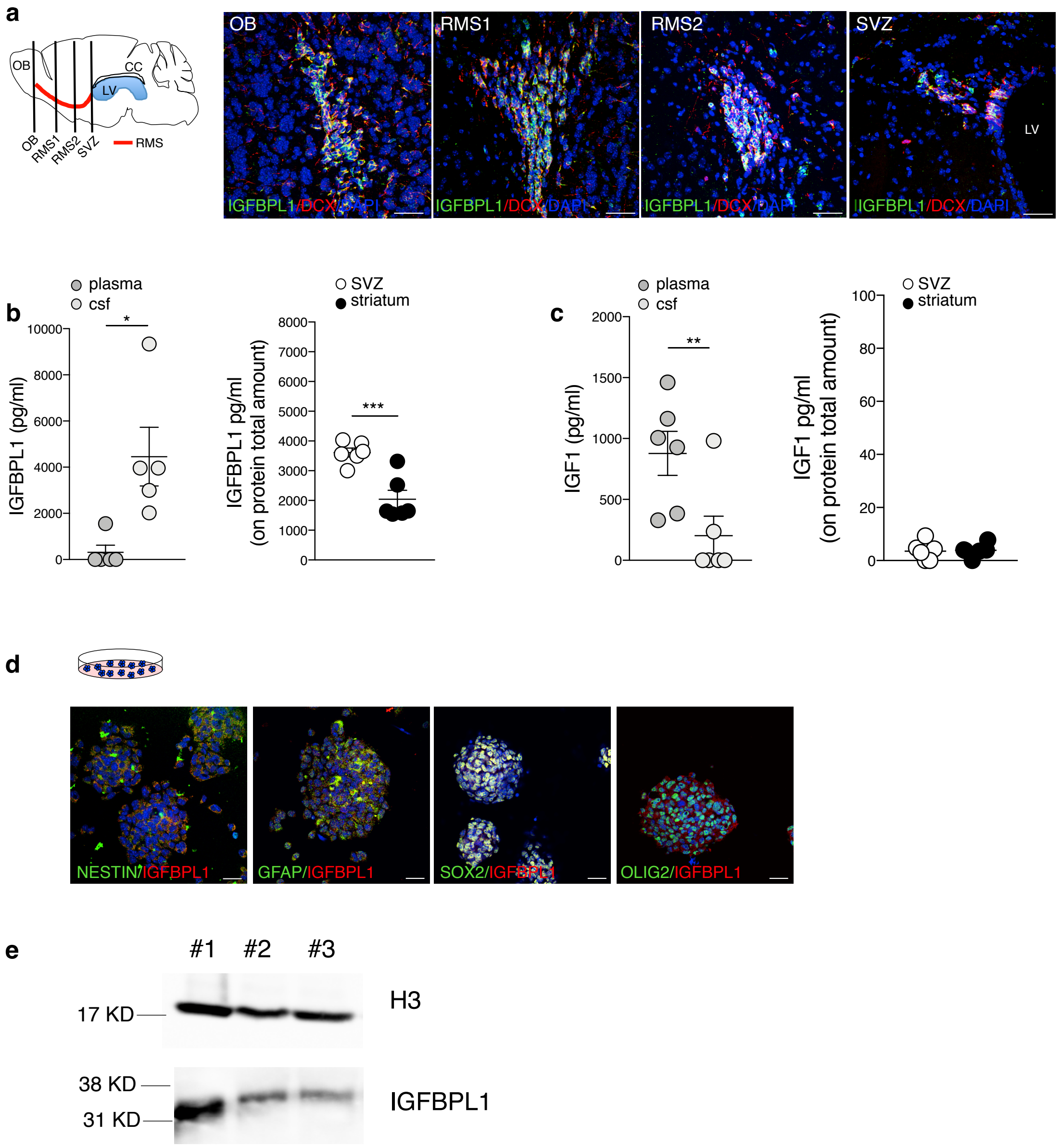

Supplementary Fig.7 Analysis of the expression of IGFBPL1 in the RMS, plasma, CSF and brain.

a Schematic representation of the sections along the rostral migratory stained. Representative confocal images at the level of the the olfactory bulb (OB), at the rostral migratory stream (RMS1 and RMS2) and at the SVZ of C57Bl/6 mice labelled for IGFBPL1 in green and DCX in red. Nuclei

in blue were stained by DAPI. Scale bar: 25 µm. **b and c** Quantification of IGFBPL1 (b) and IGF-1 (c) protein by ELISA in the plasma, CSF (n=5-6 mice), SVZ and striatum of C57Bl/6 mice (n=6 mice). Values represent mean ± SEM. In b *p=0.013 and ***p=0.0008, in c **p=0.0094, unpaired two-tailed t-test. **d** Representative confocal images of mouse neurospheres labelled for IGFBPL1 in red, and either NESTIN, GFAP, SOX2 or OLIG2 in green. Nuclei in blue were stained by DAPI. Scale bar: 25 µm. **S**taining was performed 3 times. **e** Western Blot analysis showing H3 and IGFBPL1 protein levels in mouse neurospheres (line #111107; n= 3). Source data are provided as a Source Data file.

Supplementary Fig.8

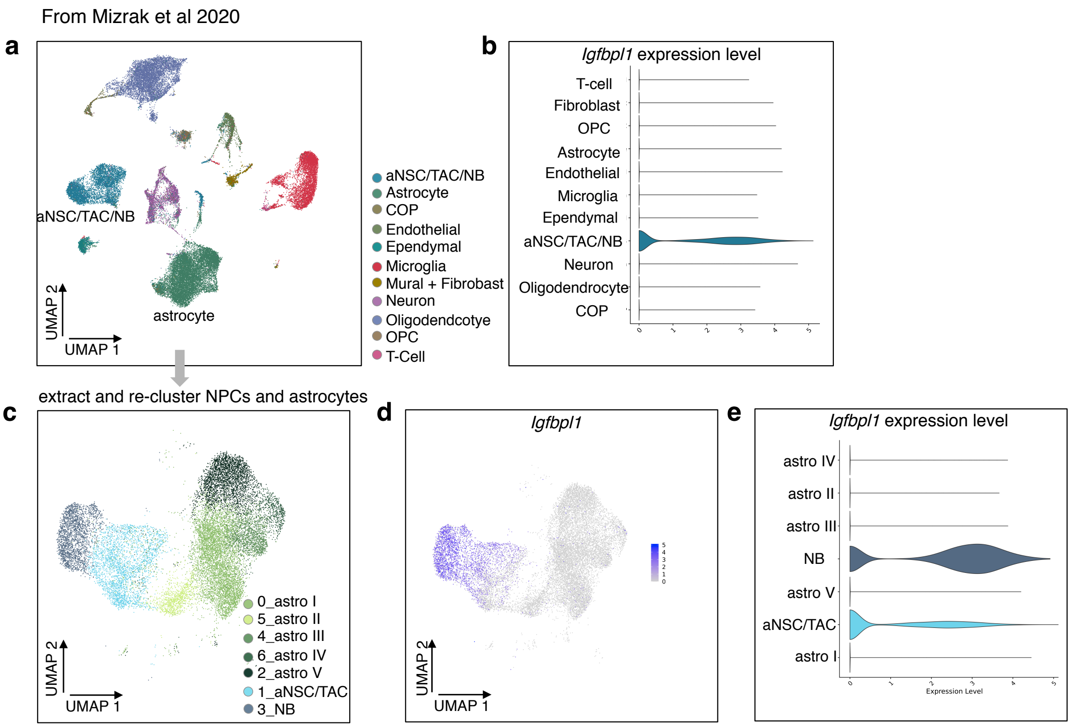

Supplementary Fig.8 ScRNA seq analysis in the mouse SVZ

a UMAP projection of major cell types of the lateral and septal adult ventricular-SVZ tissue of the scRNAseq dataset of (*22*). **b** Violin plot showing expression levels of *Igfbpl1* in the cell clusters identified in **a**. **c** UMAP projection of the astrocyte and neural precursor cells (aNSC/TAC/NB) extracted from **a** and re-clustered. Major cell population of the astrocyte or NPC lineage are shown. **d** Feature plot showing the expression pattern of *Igfbpl1* of the in **c** visualized cell clusters. **e** Violin plot showing expression levels for *Igfbpl1* in the cell clusters identified in **c**. Abbreviations: Astro, astrocytes; COP, committed oligodendrocyte precursors; OPC, oligodendrocyte precursor cell; NB, neuroblasts; aNSC, adult neural stem cells; TAC, transit amplifying cells. Source data are provided as a Source Data file

Supplementary Fig.9

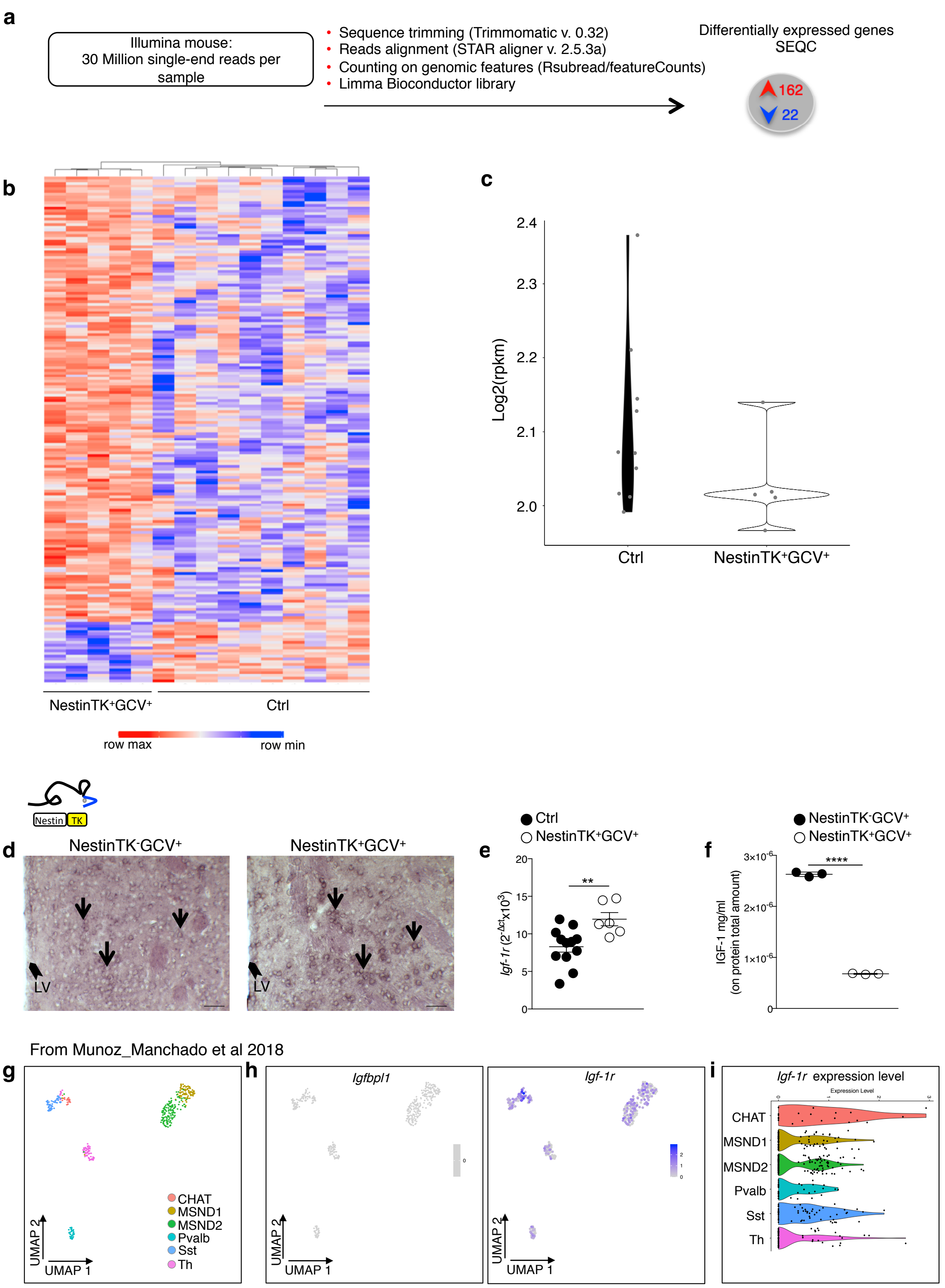

Supplementary Fig.9 Gene expression analysis in the striatum of SVZ-eNPC ablated mice

a Workflow of RNA-seq gene expression analysis in control and NestinTK$^+$GCV$^+$ treated mice. **b** Heatmap of hierchical clustering of expression values of 22 down regulated genes and 162 up regulated genes with ≥2-fold change in control and NestinTK$^+$GCV$^+$ mice. (blue means downregulation, red upregulation) n=5 mice per group. **c** Violin plot of *Igf-1* pathway average expression in control mice compared to NestinTK$^+$GCV$^+$ mice. *Igf-1* pathway was retrieved from Reactom Database. (Pre-ranked GSEA: FDR= 0.03, NES= -1.82). **d** Representative images of the *in situ* hybridization for *Igf-1r* at the level of the striatum in control and NestinTK$^+$GCV$^+$ mice. Scale bar: 50 µm. Protocol performed 2 times. **e** The graph shows the quantification obtained by rt-PCR for *Igf-1r* in the striatum of the control and NestinTK$^+$GCV$^+$ mice. Values represent the mean ± SEM. **p= 0.0075, unpaired two-tailed t-test. n=12 ctrl mice and n=6 NestinTK$^+$GCV$^+$ mice. **f** Quantification of IGF-1 protein by Elisa in the SVZ of NestinTK$^+$GCV$^+$ and NestinTK$^+$GCV$^+$ mice (n=3 different pools of the SVZ derived from 3 mice per group). Values represent means ± SEM ****p=1.9*10^{-7} unpaired two-tailed t-test. **g** UMAP pojection of major cells clusters identified as neurons or interneurons of the scRNAseq dataset. **h** Feature plots showing the expression patter of *Igfbpl1* and *Igf-r1* in the visualized cell cluster. **i** Violin plot for *Igf-1r* expression levels in the cell clusters identified in **g**. Source data are provided as a Source Data file.

Supplementary Fig.10

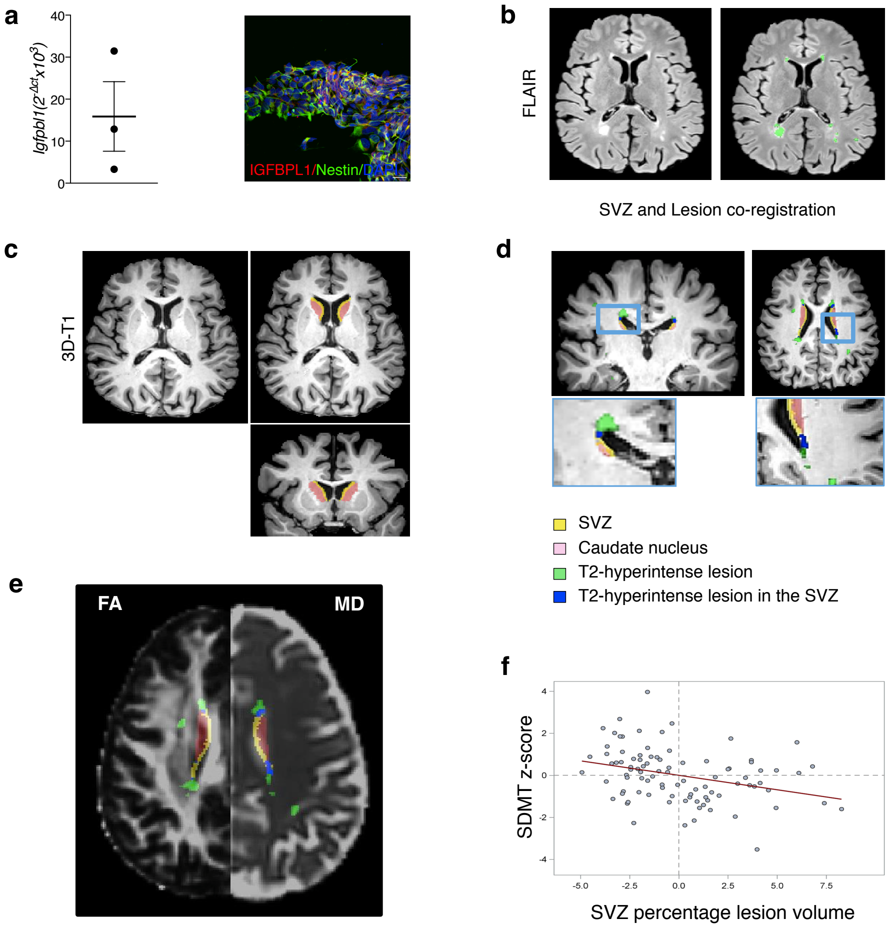

Supplementary Fig.10 SVZ damage correlates with impaired cognitive performances in patients affected by neurodegeneration

a Quantitative PCR of *Igfbpl1* expressed gene in iPS-derived NPCs obtained from fibroblasts of three MS patients. Values represent mean ± SEM. Immunofluorescent staining (n=3 independent staining) showing IGFBPL1 (in red) and NESTIN (in green) expression in iPS-derived NPCs from MS patient. Scale bar: 20 μm. **b to f** T2-hyperintense lesion masks were segmented on FLAIR images **(b)** and co-registered on high-resolution T1-weighted sequences **(d)**, fractional anisotropy (FA) and mean diffusivity (MD) maps **(e)**. Caudate nuclei were automatically segmented on high-resolution T1-weighted sequences. According to anatomical references, a SVZ mask was segmented on T1-weighted images in the Montreal Neurological Institute space and then registered

on native T1-weighted image **(d)**, FA and MD maps **(e)**. Age-, sex- and phenotype-adjusted partial correlation between Symbol Digit Modalities Test (SDMT) and SVZ percentage lesion volume are plotted in **f**. Data from 97 MS patients (mean age 36.8 ± 7.56 years, female/male [F/M] =55/42, median EDSS 2.0, median disease duration 5.0 years) and 43 age- and sex-matched healthy controls (HC, 34.8 ± 6.52 years, F/M=19/24) are reported (see details in Material and Methods). Source data are provided as a Source Data file.

Supplementary Table 1

	At the end of ablation- raw data	raw p	ranked	1 month recovery- raw data	raw p	ranked
Total SS (SSE)	SS=1502.0 (V=0.67, 2239 d.f.)			SS=1627.1 (V=0.73, 2239 d.f.)		
SS within (SSW)	SS=679.1 (V=0.35, 1920 d.f.)			SS=578.6 (V=0.30, 1920 d.f.)		
SS_among_Times (SS_T)	SS=174.4 (V=9.18, 19 d.f.)	p < 0.0001	p < 0.0001	SS=163.6 (V=8.61, 19 d.f.)	p < 0.0001	p < 0.0001
SS_among_Days (SS_D)	SS=21.2 (V=7.05, 3 d.f.)	p < 0.0001	p > 0.3324	SS=67.8 (V=22.58, 3 d.f.)	p < 0.0001	p < 0.0001
SS_between_Genotypes (SS_G)	SS=0.6 (V=0.58, 1 d.f.)	p > 0.354	p > 0.3357	SS=0.3 (V=0.29, 1 d.f.)	p > 0.533	p < 0.0001
SS_between_Stimuli (SS_S)	SS=241.8 (V=241.76, 1 d.f.)	p < 0.0001	p < 0.0001	SS=261.7 (V=261.70, 1 d.f.)	p < 0.0001	p < 0.0001
SS_Time_x_Day (SS_{TxD})	SS=55.2 (V=0.97, 57 d.f.)	p > 0.0126	p > 0.7375	SS=84.7 (V=1.48, 57 d.f.)	p < 0.0001	p > 0.1431
SS_Time_x_Genotype (SS_{TxG})	SS=6.8 (V=0.36, 19 d.f.)	p > 0.9447	p > 0.5593	SS=4.5 (V=0.24, 19 d.f.)	p > 0.9977	p > 0.6967
SS_Time_x_Stimulus (SS_{TxS})	SS=121.2 (V=6.38, 19 d.f.)	p < 0.0001	p < 0.0001	SS=181.3 (V=9.54, 19 d.f.)	p < 0.0001	p < 0.0001
SS_Day_x_Genotype (SS_{DxG})	SS=5.3 (V=1.77, 3 d.f.)	p > 0.0466	p > 0.0764	SS=11.1 (V=3.71, 3 d.f.)	p < 0.0015	p < 0.0002
SS_Day_x_Stimulus (SS_{DxS})	SS=88.2 (V=29.4, 3 d.f.)	p < 0.0001	p < 0.0001	SS=139.6 (V=46.52, 3 d.f.)	p < 0.0001	p < 0.0001
SS_Genotype x_Stimulus (SS_{GxS})	SS=1.1 (V=1.6, 1 d.f.)	p > 0.2071	p > 0.1358	SS=0.1 (V=0.08, 1 d.f.)	p > 0.7505	p > 0.4285
SS_Time_x_Day_x_Genotype (SS_{TxDxG})	SS=13.7 (V=0.24, 57 d.f.)	p > 0.9999	p > 0.9998	SS=8.5 (V=0.15, 57 d.f.)	p > 0.9999	p > 0.9999
SS_Time_x_Day x_Stimulus (SS_{TxDxG})	SS=64.1 (V=1.12, 57 d.f.)	p < 0.0008	p > 0.149	SS=98.8 (V=1.73, 57 d.f.)	p < 0.0001	p < 0.0041
SS_Time_x_Genotype _x_Stimulus (SS_{TxGxS})	SS=10.6 (V=0.56, 19 d.f.)	p > 0.6808	p > 0.4945	SS=3.5 (V=0.18, 19 d.f.)	p > 0.9992	p > 0.9706
SS_Day_x_Genotype _x_Stimulus (SS_{DxGxS})	SS=6.3 (V=2.11, 3 d.f.)	p > 0.0247	p > 0.3411	SS=12.2 (V=4.08, 3 d.f.)	p < 0.0008	p < 0.0035
CSA day4: SS_among_Times		p < 0.0001	p < 0.0001	CSA day4: time	p < 0.0001	p < 0.0001
CSA day4: SS_btw_Genotypes		p < 0.001	p < 0.0064	CSA day4: genotype	p > 0.1252	p > 0.6028
CSA day4:SS Time x Genotype		p > 0.7179	p > 0.9128	CSA day4: time x genotype	p > 0.9993	p > 0.9999

Supplementary Table 1 (continued)

mean Time(0.5) = -0.44		$p < 0.0001$	$p < 0.0001$	mean Time(0.5) = -0.47	$p < 0.0001$	$p < 0.0001$
mean Time(1.5) = -0.39		$p < 0.0001$	$p < 0.0001$	mean Time(1.5) = -0.45	$p < 0.0001$	$p < 0.0001$
mean Time(2.5) = -0.40		$p < 0.0001$	$p < 0.0001$	mean Time(2.5) = -0.30	$p < 0.0001$	$p < 0.0002$
mean Time(3.5) = -0.33		$p < 0.0001$	$p < 0.0002$	mean Time(3.5) = -0.33	$p < 0.0001$	$p < 0.0002$
mean Time(4.5) = -0.29		$p < 0.0001$	$p < 0.0016$	mean Time(4.5) = -0.26	$p < 0.0004$	$p < 0.0009$
mean Time(5.5) = -0.22		$p < 0.0003$	$p > 0.0132$	mean Time(5.5) = -0.21	$p < 0.0018$	$p > 0.0868$
mean Time(6.5) = -0.24		$p < 0.0003$	$p < 0.0101$	mean Time(6.5) = -0.18	$p < 0.0085$	$p > 0.2512$
mean Time(7.5) = -0.08		$p > 0.1404$	$p > 0.3653$	mean Time(7.5) = -0.13	$p > 0.0472$	$p > 0.28$
mean Time(8.5) = -0.12		$p > 0.0448$	$p > 0.2046$	mean Time(8.5) = -0.08	$p > 0.1634$	$p > 0.1078$
mean Time(9.5) = -0.02		$p > 0.3969$	$p > 0.2332$	mean Time(9.5) = 0.01	$p > 0.4403$	$p > 0.0908$
mean Time(10.5) = 0.09		$p > 0.1163$	$p > 0.0136$	mean Time(10.5) = 0.08	$p > 0.1617$	$p > 0.0113$
mean Time(11.5) = 0.15		$p > 0.0259$	$p > 0.033$	mean Time(11.5) = 0.15	$p > 0.0358$	$p < 0.0012$
mean Time(12.5) = 0.21		$p < 0.004$	$p > 0.0131$	mean Time(12.5) = 0.11	$p > 0.074$	$p > 0.0387$
mean Time(13.5) = 0.22		$p < 0.0027$	$p < 0.0009$	mean Time(13.5) = 0.21	$p < 0.0068$	$p < 0.0006$
mean Time(14.5) = 0.31		$p < 0.0002$	$p < 0.0012$	mean Time(14.5) = 0.27	$p < 0.0012$	$p < 0.0016$
mean Time(15.5) = 0.24		$p < 0.0014$	$p < 0.0079$	mean Time(15.5) = 0.23	$p < 0.0044$	$p > 0.0208$
mean Time(16.5) = 0.23		$p < 0.0028$	$p < 0.0023$	mean Time(16.5) = 0.30	$p < 0.0004$	$p < 0.005$
mean Time(17.5) = 0.39		$p < 0.0001$	$p < 0.0001$	mean Time(17.5) = 0.36	$p < 0.0001$	$p < 0.0025$
mean Time(18.5) = 0.29		$p < 0.0003$	$p < 0.0001$	mean Time(18.5) = 0.30	$p < 0.0002$	$p < 0.0085$
mean Time(19.5) = 0.41		$p < 0.0001$	$p < 0.0002$	mean Time(19.5) = 0.38	$p < 0.0001$	$p > 0.0109$
mean Day(1) = -0.12		$p < 0.0001$	$p > 0.0423$	mean Day(1) = -0.25	$p < 0.0001$	$p < 0.0001$
mean Day(2) = -0.06		$p > 0.0177$	$p > 0.1621$	mean Day(2) = -0.07	$p > 0.0106$	$p < 0.0003$
mean Day(3) = 0.07		$p < 0.0088$	$p > 0.2125$	mean Day(3) = 0.13	$p < 0.0001$	$p > 0.0643$
mean Day(4) = 0.12		$p < 0.0002$	$p > 0.4785$	mean Day(4) = 0.19	$p < 0.0001$	$p > 0.1443$
mean Genoty.(Ctl) = 0.02		$p > 0.1388$	$p > 0.134$	mean Genotype(Ctl) = 0.01	$p > 0.2331$	$p < 0.0001$
mean Genoty.(Abl) = -0.01		$p > 0.2057$	$p > 0.2062$	mean Genotype(Abl) = -0.01	$p > 0.2904$	$p < 0.0001$
mean Stim.(CSA) = 0.33		$p < 0.0001$	$p < 0.0001$	mean Stim(CSA) = 0.34	$p < 0.0001$	$p < 0.0001$
mean Stim.(CSB) = -0.33		$p < 0.0001$	$p < 0.0001$	mean Stim(CSB) = -0.34	$p < 0.0001$	$p < 0.0001$

Abbreviations: SS = sum of square departures; V= Variance; d.f.= degree of freedom. The 4-way analysis of variance was performed in a nonparametric way.

Supplementary Table 2. Pearson raw and partial (age-, sex-, and phenotype-adjusted) correlations between clinical, and structural MRI variables and caudate volume in MS patients.

Variable	Raw r	Raw p	Adjusted r	Adjusted p
Disease duration	-0.37	0.0002	-0.16	0.14
SVZ percentage LV	-0.39	<0.0001	0.33	**0.0011**
SVZ normal-appearing FA	0.22	0.03	0.18	0.09
SVZ intralesional FA	0.45	<0.0001	0.36	**0.0004**
SVZ normal-appearing MD	0.50	<0.0001	-0.44	**<0.0001**
SVZ intralesional MD	-0.51	<0.0001	-0.45	**<0.0001**
NBV	0.64	<0.0001	0.54	**<0.0001**
NGMV	0.60	<0.0001	0.45	**<0.0001**
NWMV	0.39	<0.0001	0.39	**0.0001**
Log T2-hyperintense LV	0.47	<0.0001	-0.39	**0.0001**
Cortical thickness	0.56	<0.0001	0.48	**<0.0001**
WM FA	0.41	<0.0001	0.34	**0.0008**
WM MD	-0.58	<0.0001	-0.47	**<0.0001**

Abbreviations: FA=fractional anisotropy; LV=lesion volume; Log=logarithm; MD=mean diffusivity; NBV=normalized brain volume; NGMV=normalized grey matter volume; NWMV=normalized white matter volume; SVZ=subventricular zone; WM=white matter.

Supplementary Table 3. Age-, sex-, and phenotype-adjusted stepwise regression (dependent variable: caudate volume) using the Schwarz information criterion as inclusion/exclusion criterion for variable selection.

Variable	Standardized estimate	Standard error	t value	p value
*Age [years]**	0.009	0.014	0.09	0.92
*Sex [male]**	-0.17	0.19	-2.29	0.02
*Sex [female]**	0	-	-	-
*Phenotype [progressive MS]**	0.025	0.24	0.32	0.75
*Phenotype [relapsing MS]**	0	-	-	-
NBV	0.32	0.002	2.92	0.004
SVZ percentage LV	-0.16	0.03	-2.17	0.03
Cortical thickness	0.31	1.27	3.34	0.001
SVZ intralesional MD	-0.24	1.85	-2.78	0.007

*Forced into the model.

Abbreviations: LV=lesion volume; MD=mean diffusivity; MS=multiple sclerosis; NBV=normalized brain volume; SVZ=subventricular zone.

Supplementary Table 4. Pearson raw and partial (age-, sex-, and phenotype-adjusted) correlations between clinical, and structural MRI variables and SDMT z-scores in MS patients.

Variable	Raw r	Raw p	Adjusted r	Adjusted p
Disease duration	-0.16	0.11	0.06	0.54
SVZ percentage LV	-0.36	0.0004	-0.31	**0.003**
SVZ normal-appearing FA	0.15	0.14	0.09	0.38
SVZ lesional FA	0.36	0.0004	0.23	**0.03**
SVZ normal-appearing MD	-0.33	0.001	-0.25	**0.02**
SVZ lesional MD	-0.33	0.001	-0.20	0.06
NBV	0.29	0.005	0.05	0.65
NGMV	0.26	0.01	0.05	0.45
NWMV	0.19	0.07	0.15	0.17
*NDGMV**	0.24	0.03	0.02	0.85
N-caudate volume	0.26	0.01	0.06	0.56
Log T2-hyperintense LV	-0.37	0.0002	-0.30	**0.005**
Cortical thickness	0.19	0.07	-0.01	0.92
WM FA	0.20	0.06	0.07	0.53
WM MD	-0.24	0.02	-0.06	0.59

Abbreviations: FA=fractional anisotropy; LV=lesion volume; Log=natural logarithm; MD=mean diffusivity; NBV=normalized brain volume; NGMV=normalized grey matter volume; NWMV=normalized white matter volume; SVZ=subventricular zone; WM=white matter.

Supplementary Table 5. Age-, sex-, and phenotype-adjusted stepwise regression (dependent variable: SDMT z-score) using the Schwarz information criterion as inclusion/exclusion criterion for variable selection.

Variable	Standardized estimate	Standard error	t value	p value
*Age [years]**	-0.37	0.02	-3.81	**0.0003**
*Sex [male]**	0.01	0.26	0.15	0.88
*Sex [female]**	0	-	-	-
*Phenotype [progressive MS]**	0.02	0.31	0.22	0.82
*Phenotype [relapsing MS]**	0	-	-	-
SVZ percentage LV	-0.30	0.04	-3.22	0.002

*Forced into the model.

Abbreviations: LV=lesion volume; MS=multiple sclerosis; SVZ=subventricular zone.

Supplementary Methods

Analysis of the behavioural data

The behavioural data – nose poking frequency during a 20-s significant (CSA+) or dummy (CSB-) sound stimulus, followed in case of CSA+ by the presentation of a food pellet, were examined to detect whether there were differences in general activity and/or in the learning curve between control and NestinTK$^+$GCV$^+$ mice. In order to examine the learning curve a full multifactorial analysis had to be performed, considering that each observation (number of pokes in 30 trials during one second of stimulus A or B on a certain day for a particular mouse genotype) could be predicted as the result of:

- a general overall poking mean frequency (GM)
- an additive effect of the stimulus, E(S) (presumably +E(S) for CSA+, –E(S) for CSB-)
- an additive effect for the day of training E(D) (presumably negative for days 1-2 and positive for days 3-4)
- an additive effect for the time during the stimulus, E(T) (presumably negative at 0 s and increasing toward the end of the stimulus, at least on days 3-4 of training)
- a possible additive effect for genotype, E(G)
- a possible additive effect for each possible interaction between two factors, E(S×D), E(S×T), E(S×G), E(D×T), E(D×G), E(T×G)
- a possible additive effect for each possible interaction among three factors, E(S×D×T), E(S×D×G), E(S×T×G), E(D×T×G)
- a possible additive effect for interaction among all factors E(S×D×T×G)

The behavioural data presented were far from normally distributed, so the multiple-way analysis had to be performed in a nonparametric way.

The data were classified according to four factors (stimulus, genotype, day and time) and the first part of a standard ANOVA was performed, obtaining a grand mean (GM) and a total sum of square errors (SSE); the latter was decomposed into a sum of square errors within groups (SSW), a sum of square errors among the distinct classes for each factor (S_S, SS_G, SS_D, SS_T, for stimulus, genotype, day and time, respsctively) and a sum of square errors for interactions between two factors ($SS_{S\times G}$, $SS_{S\times D}$, etc.) or among three factors ($SS_{S\times G\times D}$, $SS_{S\times G\times T}$, etc.). Because of the non-normal distributions, the corresponding variances were not compared to the variance within groups, using Fisher test (variance ratio), but the probability of obtaining the observed value, for each component of the sum of square errors, was computed numerically, by repeating the same analysis on 10,000 random permutations of the experimental values. This procedure ('bootstrapping') produced a probability

distribution for each component of the sum of square errors, from which the probability and the significance of each observation could be estimated.
Since many estimates and comparisons (close to 50) were performed, the significance threshold was lowered to $p < 0.001$, according to a Bonferroni post-hoc approach.

The results of this preliminary analysis are reported in the Results section; they indicated that ablated animals poked more frequently than controls.

Furthermore the significant interactions of genotype with day and stimulus suggested that the learning curves might also be different between the two groups. However, the described model was additive; spurious interactions would appear if the difference among animals, and between genotypes, were not additive, but rather due to a scale factor (i.e. multiplicative), determined for each animal by its own tendency to poke.

Indeed, there was a significant variability among animals, which seemed to persist along the various trials and days. This is illustrated in the Supplementary Fig. 3.

Consistently, the frequencies for the two groups were highly correlated during the stimuli and across the days (correlation coefficient $R = 0.871$, $p = 1.15e-050$) with a slope of 1.20 (20% higher poking frequencies in ablated than controls). This suggests that the effect of genotype is not additive but rather consists in a scale factor, ablated mice being generally more active in poking.

This might be the origin of the observed interactions of genotype with day and stimulus. In fact, this would modify the original model (1):

- $\text{Frequency} = GM + E(S) + E(D) + E(T) + E(G) + E(S \times D) + E(S \times T) + E(S \times G) + E(D \times T) + E(D \times G) + E(T \times G) + E(S \times D \times T) + E(S \times D \times G) + E(S \times T \times G) + E(D \times T \times G)$

into the model (2):

- $\text{Frequency} = E(G) \cdot [GM + E(S) + E(D) + E(T) + E(S \times D) + E(S \times T) + E(S \times G) + E(D \times T) + E(D \times G) + E(T \times G) + E(S \times D \times T) + E(S \times D \times G) + E(S \times T \times G) + E(D \times T \times G)$

If the interactions of genotype with other factors were genuine (indicating a difference in learning curve) they should maintain a significance in this second model. Thus, in order to more properly analyse possible differences in the learning curves of the animals, the values for each mouse were normalized for the average value of its own overall poking frequency.
The results of the ensuing four-way analysis on the normalized values are reported in the text.

Supplementary Figure 11: unprocessed gels of Supplementary Figures 7e

H3

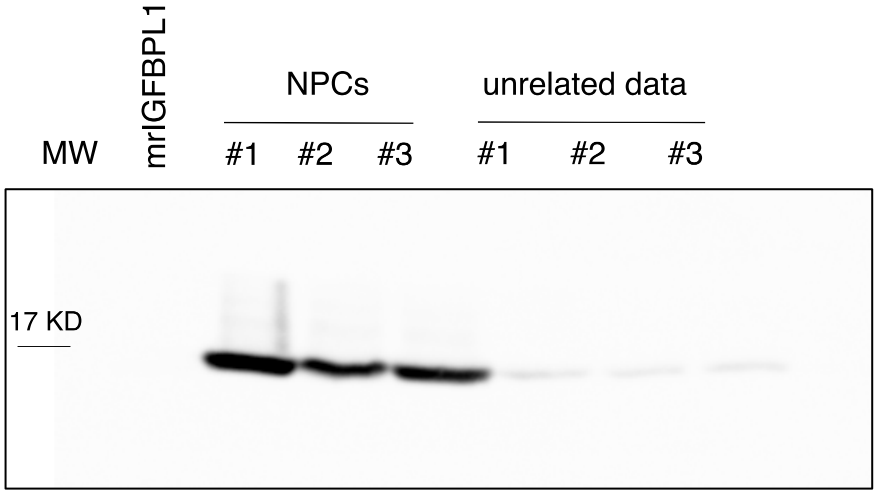

IGFBPL1

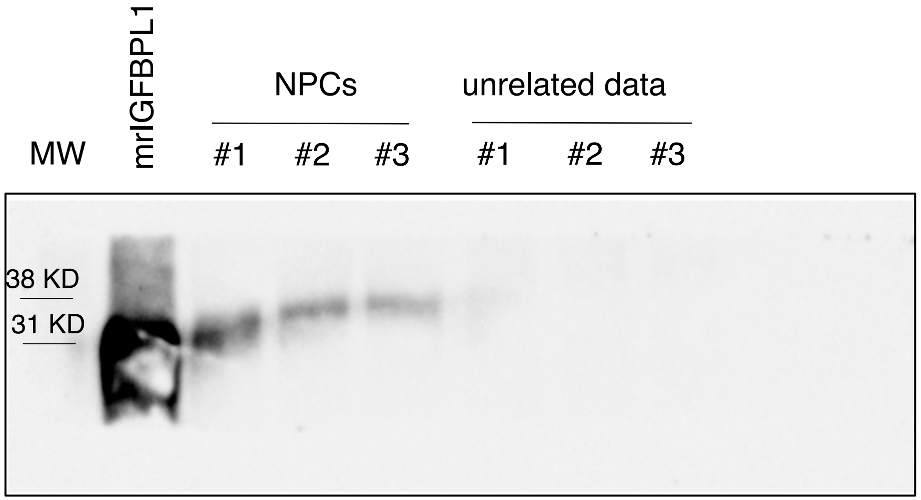

Partial covered on lane mrIGFBPL1 (mouse recombinant IGFBPL1 used as positive control) for optimization of signal detection on lane #1, #2 and #3 since signal of positive control was saturating the image.

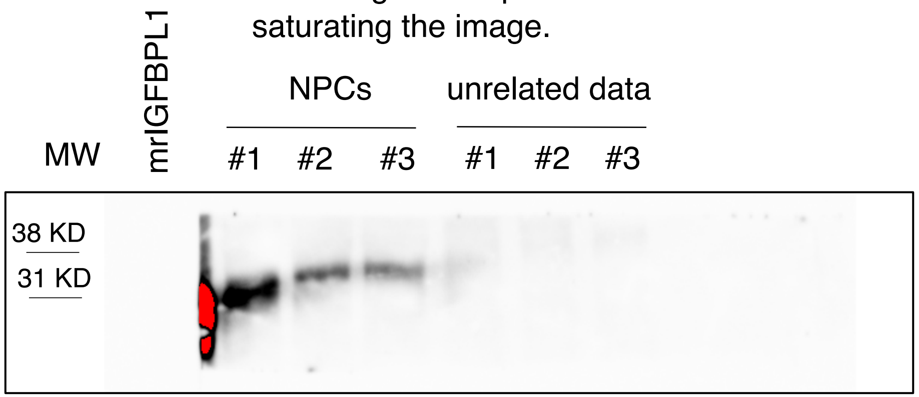

Supplement: Supplementary file 1 — Supplementary Information [file 41467_2022_35341_MOESM1_ESM.pdf]
