# Supplementary material for: Neural precursor cells tune striatal connectivity through the release of IGFBPL1
Source: Nat Commun. 2022 Dec 8;13:7579. doi: 10.1038/s41467-022-35341-y (PMC9731988; doi:10.1038/s41467-022-35341-y)
Supplement: Supplementary file 2 — Reporting Summary [file 41467_2022_35341_MOESM2_ESM.pdf]

nature portfolio

Corresponding author(s): Prof. Gianvito Martino

Last updated by author(s): 15/11/22

Reporting Summary

Nature Portfolio wishes to improve the reproducibility of the work that we publish. This form provides structure for consistency and transparency in reporting. For further information on Nature Portfolio policies, see our Editorial Policies and the Editorial Policy Checklist.

Statistics

For all statistical analyses, confirm that the following items are present in the figure legend, table legend, main text, or Methods section.

n/a	Confirmed	
☐	☒	The exact sample size (*n*) for each experimental group/condition, given as a discrete number and unit of measurement
☐	☒	A statement on whether measurements were taken from distinct samples or whether the same sample was measured repeatedly
☐	☒	The statistical test(s) used AND whether they are one- or two-sided<br>*Only common tests should be described solely by name; describe more complex techniques in the Methods section.*
☐	☒	A description of all covariates tested
☐	☒	A description of any assumptions or corrections, such as tests of normality and adjustment for multiple comparisons
☐	☒	A full description of the statistical parameters including central tendency (e.g. means) or other basic estimates (e.g. regression coefficient) AND variation (e.g. standard deviation) or associated estimates of uncertainty (e.g. confidence intervals)
☐	☒	For null hypothesis testing, the test statistic (e.g. *F*, *t*, *r*) with confidence intervals, effect sizes, degrees of freedom and *P* value noted<br>*Give P values as exact values whenever suitable.*
☒	☐	For Bayesian analysis, information on the choice of priors and Markov chain Monte Carlo settings
☒	☐	For hierarchical and complex designs, identification of the appropriate level for tests and full reporting of outcomes
☒	☐	Estimates of effect sizes (e.g. Cohen's *d*, Pearson's *r*), indicating how they were calculated

Our web collection on statistics for biologists contains articles on many of the points above.

Software and code

Policy information about availability of computer code

Data collection	-Microsoft Excel 2011, version 14.7.2.<br>-For images acquisition: Leica application suite advanced fluorescence software, version 2.7.3.9723.<br>-For behavioural data: Med-Associates Product #ENV-307A associated with a Med-PC controlled all the experimental events and recorded the time at which events occurred with 10-ms resolution.<br>- For electrophysiology data registration and analysis: pClamp10 software (Molecular Devices).<br>- For gene expression data collection: QuantStudio 3 and the Thermofisher ConnectTM software.<br>- For Rnaseq: reads were trimmed using Trimmomatic, version 0.32, to remove adapters and to exclude low-quality reads from the analysis. The remaining reads were then aligned to the reference genome mm10, Gencode version M16, using STAR aligner, version 2.5.3a.<br>- For MRI data collection: 3.0 Tesla Philips Ingenia CX scanner with a dS Head 32-channel receiver coil.
Data analysis	-For data analysis we used Microsoft Excel 2011, version 14.7.2.<br>-For statistical analysis we used Prism 7 version 7.0.<br>-For gene expression analysis we used Quantum Studio software in iCloud on Thermofischer website (Thermofisher ConnectTM).<br>-For Image analyses we used Adobe Photoshop CC software version 14.0 (Adobe Systems Incorporated) or ImageJ 1.52q (NIH software).<br>-For cell tracing and analysis Neurolucida Explorer and using the morphometric analysis provided in the Neurolucida® Explorer software package. Spine length and spine density was determined using the measuring tool on the StereoInvestigator software (MicroBrightField).<br>- For electrophysiological data analysis we used Origin 9.1 (Origin Lab, Northampton, MA, USA).<br>- For RNA-sequencing: reads were trimmed using Trimmomatic, version 0.32, to remove adapters and to exclude low-quality reads from the analysis. The remaining reads were then aligned to the reference genome mm10, Gencode version M16, using STAR aligner, version 2.5.3a. Gene expression read counts were exported and analysed in R environment (v. 3.1.1) to identify differentially expressed genes (DEGs), using the limma Bioconductor library. The gene-sets included in the GSEA analyses were obtained from Canonical Pathways, Hallmark and the Gene Ontology (GO) collections as they are reported in the MSigDB database file://localhost/(https/::www.gsea-msigdb.org:gsea:msigdb:index.jsp).

- Data ATAC-seq: Reads were demultiplexed using cell ranger-atac (v1.0.1). Identification of cell barcodes was performed using umitools (v1.0.1) using R2 as input. Read tags were aligned to hg38 reference genome using bwa mem v0.7.12 [arXiv:1303.3997 (q-bio.GN)]. Gene activities were calculated as per cell coverage over the gene body interval extended to 2kb upstream the TSS, using gencode v30 as gene model. Data were processed using scanpy v1.4.6.
- For single cells RNA sequencing mouse: The full dataset was processed with the Seurat standard pipeline in R environment (R v.4.0.3, Seurat v.3.2.2): Human: raw data were downloaded from ArrayExpress (E-MTAB-10220) and processed with scanpy (doi: 10.1186/s13059-017-1382-0). Droplet doublets were identified with scrublet (doi: 10.1016/j.cels.2018.11.005). Batch removal was performed using Harmony (doi: 10.1038/s41592-019-0619-0).
- Local field potential's epochs were visually examined and power spectra of artefact-free segments were computed using fast Fourier transforms by using the commercial software NeuroExplorer (Plexon).
- MRI data were analysed by FSL SIENAx software and FMRIB's Integrated Registration and Segmentation Tool (FIRST) pipeline (FMRIB, Oxford, UK), FreeSurfer 6.0 software suite (http://surfer.nmr.mgh.harvard.edu/), FSL software (FMRIB, Oxford, UK).

For manuscripts utilizing custom algorithms or software that are central to the research but not yet described in published literature, software must be made available to editors and reviewers. We strongly encourage code deposition in a community repository (e.g. GitHub). See the Nature Portfolio guidelines for submitting code & software for further information.

Data

Policy information about availability of data

All manuscripts must include a data availability statement. This statement should provide the following information, where applicable:
- Accession codes, unique identifiers, or web links for publicly available datasets
- A description of any restrictions on data availability
- For clinical datasets or third party data, please ensure that the statement adheres to our policy

- The data that support the findings of this study are available in the Souce data file and more information could give from the corresponding author on reasonable request.
- The gene-sets included in the GSEA analyses were obtained from Canonical Pathways, Hallmark and the Gene Ontology (GO) collections as they are reported in the MSigDB database (https://www.gsea-msigdb.org/gsea/msigdb/index.jsp). The accession number for these data is GSE165815. We provide a temporary token for editor and reviewers to access the data: gjqbgmespvwhrof. The data will become accessible to readers at the time of the online publication.
- hg38 reference genome v0.7.12: arXiv:1303.3997 (q-bio.GN)
- Single cell SVZ RNAseq: ref. GEO accession number GSE109447, from Mizrak et al.
- Single cell Striatum RNAseq: ref. GEO accession number GSE97478, from Munoz-Manchado et al.
- Human single cell and data ATAC-seq: previously published single cell data for iPSC and NPC (Tedesco et al,) were available from ArrayExpress (https://www.ebi.ac.uk/biostudies/arrayexpress) with the following accession numbers: E-MTAB-10220 for scRNA-seq and E-MTAB-10218 for chromatin accessibility.

Field-specific reporting

Please select the one below that is the best fit for your research. If you are not sure, read the appropriate sections before making your selection.

☒ Life sciences ☐ Behavioural & social sciences ☐ Ecological, evolutionary & environmental sciences

For a reference copy of the document with all sections, see nature.com/documents/nr-reporting-summary-flat.pdf

Life sciences study design

All studies must disclose on these points even when the disclosure is negative.

Sample size	- For behavioural experiments to establish the sample size we used the G-power v3.1.9.4 (Heinrich-Heine-Universität Düsseldorf) with One-way Anova statistical test, with 80% of power and 5% of alpha error. From the analysis we obtained n=12 mice for group.<br>- For determining on immunostainings and the ablation efficacy we used n=4-5 sections for each mouse, n=3 mice for each group; this sample size is sufficient given the effect of the treatment and preliminary experiments and previous publications;<br>- For Golgi staining and Sholl analysis we traced and measured n=6-8 neurons for each mouse, n= 6 mice per group analysing; this sample size is sufficient given the effect of the treatment and preliminary experiments;<br>- For electrophysiological experiments we recorded cells or pairs, n=15-20 per group, with n=3-5 mice per group. We based these sample sizes on the experience and the literature.<br>- For gene expression analysis and RNA-seq we analysed n=5 mice per group. This sample size is sufficient given the effect of the treatment and preliminary experiments and previous publications;<br>- For human MRI we examined n=97 MS patients (mean age 36.8 ± 7.56 years, female/male [F/M] =55/42, median EDSS 2.0, median disease duration 5.0 years) and 43 age- and sex-matched healthy controls (HC, 34.8 ± 6.52 years, F/M=19/24). This sample size is sufficient considering the literature data.<br><br>In our experiments no sample size calculation was performed using statistical software; we decided the sample size on our experience and following our same experiments done in the past and published.
Data exclusions	No data were excluded.
Replication	We repeated the mouse immunostainings for 3 times and we observed a strong reproducibility. Golgi staining, behavioural test, RNAseq and study of local field potential were performed once.

Randomization	In our experiments we used transgenic mice that were allocated randomly to the treatment group; different transgenic groups/ treatments were randomly distributed within cages reducing possible biases; we used both female and male mice which a similar age and allocated them randomly to the treatment group. During experiments analysis was performed randomly between treatment groups. For behaviuoral experiments we used male mice.
Blinding	Data collection, experiments and analysis were performed by a researcher blinded for the treatment/ transgenic group.

Reporting for specific materials, systems and methods

We require information from authors about some types of materials, experimental systems and methods used in many studies. Here, indicate whether each material, system or method listed is relevant to your study. If you are not sure if a list item applies to your research, read the appropriate section before selecting a response.

Materials & experimental systems

n/a	Involved in the study
☐	☒ Antibodies
☐	☒ Eukaryotic cell lines
☒	☐ Palaeontology and archaeology
☐	☒ Animals and other organisms
☐	☒ Human research participants
☒	☐ Clinical data
☒	☐ Dual use research of concern

Methods

n/a	Involved in the study
☒	☐ ChIP-seq
☒	☐ Flow cytometry
☐	☒ MRI-based neuroimaging

Antibodies

Antibodies used	The following primary antibodies were used: goat anti DCX (Santa Cruz Biotechnology, product no: sc-8066, clone C-18, lot: A0614, RRID:AB_2088494), rat anti BrdU (Abcam, product no: ab6326, clone BU1/75 (ICR1)RRID:AB_2088494), rabbit anti DCX (Abcam, product no: ab77450, clone:EPR19997, RRID:AB_2088478), goat anti IGFBPL-1 (R&D Systems, product no: AF4130, RRID:AB_2279980), rabbit anti GFP (ThermoFisher, product no: a11122, lot: 1789911, RRID:AB_221569), mouse anti NeuN (Millipore, product no: MAB377, clone: A60, lot: 3045564, RRID:AB_2298772), anti mouse MBP (Millipore, product no: MAB3861, clone:12), rabbit anti GFAP (Dako, product no: Z0334, lot: 00019620, RRID:AB_10013382), rabbit anti VGAT (Synaptic System, product no: 131002), mouse anti PV (Sigma, product no: P3088, clone: PARV-19, RRID:AB_477329), rabbit anti caspase-3 (Cell Signaling, product no: 9661, clone: Asp 175, RRID:AB_2341188), mouse anti human IGFBPL1 (Santa Cruz, product no: sc-398875, clone: C-5, rabbit anti mouse Olig2 (Millipore, product no: MABN50, clone: 211F1.1, RRID:AB_10807410), mouse anti mouse Nestin (Millipore, product no: MAB353, clone: rat-401, RRID:AB_94911), rabbit anti mouse Sox2 (Abcam, product no: ab69893, clone: 9-9-3, RRID:AB_1270861), mouse anti human Nestin (Millipore, product no: MAB5326, clone: 10C2, RRID:AB_2251134), anti-digoxigenin-AP Fab fragment (product no:11093274910, Roche, RRID:AB_514497), abbit anti S100β (DAKO, product number: z0311, RRID:AB_10013383, anti-rabbit biotinylated secondary antibody (Vector Laboratories, product no: BA-1000).<br>The fluorophore-conjugated secondary antibodies are: Alexa Fluor 488, 546 and 633 Thermo Fisher Scientific. e.g. product no: A-11006, RRID:AB_2534074; product no: A-11055, RRID:AB_2534102, product no: A-11001, RRID:AB_2534069, product no: A-11008, RRID:AB_143165 and product no: A-11003, RRID:AB_2534071).
Validation	All antibodies used in our study are commercially available and validated by manufacturer. https://www.rndsystems.com/datasheet/coa; https://www.merckmillipore.com/IT/it/life-science-research/antibodies-assays/antibodies-overview/Antibody-Development-and-Validation/cFOb.qB.8McAAAFOb64qQvSS,nav; https://www.thermofisher.com/ch/en/home/life-science/antibodies/invitrogen-antibody-validation.html. https://www.abcam.com/primary-antibodies/how-we-validate-our-antibodies, https://www.cellsignal.com/about-us/cst-antibody-validation-principles, https://sysy.com/resources/antibody-validation, https://www.scbt.com/it/resources/protocols/immunofluorescence-cell-staining.

Eukaryotic cell lines

Policy information about cell lines

Cell line source(s)	Cell line source: we used following primary cell cultures:<br>- Mouse NPCs derived from the SVZ of adult C57Bl/6 mice of 6 to 8 weeks old, 18-20 gr.<br>- Human foetal NPC: the BI-0194-008 is a non-immortalized human NPC line obtained from the diencephalic and telencephalic regions of a single human caucasian male foetus at 10–12 weeks gestational age after pregnancy termination. The fetus was provided by Banca Italiana del Cordone Ombelicale Fondazione IRCCS Ca' Granda Ospedale Maggiore Policlinico, Milan, Italy.<br>- Human induced-pluripotent stem cells (iPS) derived NPCs: to avoid genetic biases, fibroblasts were isolated from skin biopsies from two pairs of monozygotic twins discordant for MS (the first pair of twins are females, age 34 at time of tissue donation, 3 years from disease onset in the affected twin; the second pair of twins are females, age 35 at time of tissue donation, 3 years from disease onset in the affected twin).
Authentication	We didn't authenticate our cell lines.

Mycoplasma contamination	All cells used were tested for Mycoplasma by PCR and we confirmed that all cells are negative.
Commonly misidentified lines (See ICLAC register)	Used cell lines are not present in the ICLAC register.

Animals and other organisms

Policy information about studies involving animals; ARRIVE guidelines recommended for reporting animal research

Laboratory animals	Adult female and male C57Bl/6 (6-8 weeks old) and transgenic mice were purchased from Charles River or generated in our animal facility in SPF conditions. The animals were housed in Ventilated Cages. The mice were kept in 12hour/12hour light/dark cycles, at 21.5°C +/- 1.5°C temperature and 55% +/- 15 humidity.
Wild animals	the study didn't involve wild animals.
Field-collected samples	the study didn't involve animals collected from the field.
Ethics oversight	Experimental procedures were approved by the Institutional Animal Care and Use Committee (no. 750, 798, 1071) of the San Raffaele Scientific Institute, Milan (Italy).

Note that full information on the approval of the study protocol must also be provided in the manuscript.

Human research participants

Policy information about studies involving human research participants

Population characteristics	For the MRI experiment, we included healthy controls (HC, mean ± standard deviation age 38.8 ± 6.25 years, female/male=19/24) and patients with multiple sclerosis (MS, mean ± standard deviation age 36.8 ± 7.56 years, female/male =55/42, median EDSS 2.0 interquartile range= 1.0–4.5, median disease duration 5.0 years, interquartile range = 1-12). Exclusion criteria were age > 50 years, history of drug or alcohol abuse, neurologic disorders (excluding MS in patients), psychiatric comorbidities, history of head trauma and contraindications to MRI. The age restriction was chosen to minimize possible confounding effects of unknown chronic small vessel disorders of the brain. Patients had a diagnosis of clinically isolated syndrome (n=4) or MS (n=93), according to 2017 revision of McDonald criteria (Thompson et al., Lancet Neurol 2018).
Recruitment	MS patients were recruited among those regularly followed-up at the MS Center of the San Raffaele Scientific Institute, Milan (Italy). Specifically, at the time of the neurological follow-up, the neurologist systematically asked to patients if they were interested into undergoing an experimental MRI protocol, with no contrast administration, for research purposes.<br>HC were recruited among health care professionals, students, and their acquaintances, therefore, there might have been a selection bias towards higher educational levels. However, since the neuropsychological performance of HC was not analyzed in this paper, this should not have affected the results.
Ethics oversight	- MRI study in humans was approved by the Ethical Committee of the San Raffaele Scientific Institute, Milan (Italy), and written informed consent was obtained from all participants at the time of data acquisition.<br>- permission to use human foetal CNS tissue was granted by the ethical committee of the San Raffaele Hospital (approval on 09/06/2016). Tissue procurement was in accordance with the declaration of Helsinki and in agreement with the ethical guidelines of the European Network for Transplantation (NECTAR).<br>- permission to generate induced-pluripotent stem cells (iPS) from MS patients' fibroblasts was granted by the ethical committee of the San Raffaele Hospital (BIOBANCA-INSPE, approved on March 9th, 2017).<br>- Human brain tissue samples were obtained from the Carlo Besta Neurological Institute (Milan, Italy). Research use of human tissue was in accordance with the Declaration of Helsinki (1964–2008) and the Additional Protocol on the Convention of Human Rights and Biomedicine concerning Biomedical Research (2005).

Note that full information on the approval of the study protocol must also be provided in the manuscript.

Magnetic resonance imaging

Experimental design

Design type	Structural MRI. Between-group comparison of conventional and microstructural measures.
Design specifications	Retrospective evaluation of brain MRI scans acquired from 97 patients with multiple sclerosis (MS) and 43 age- and sex-matched healthy controls (HC). MRI scans were obtained with a standardized protocol in a single session.
Behavioral performance measures	Lesion volume on FLAIR, brain volumes (whole brain, grey matter, white matter and deep grey matter nuclei), cortical thickness, fractional anisotropy and mean diffusivity. Lesion volume and microstructural damage (fractional anisotropy and mean diffusivity) were also assessed within a in-home made mask of the subventricular zone, designed according to anatomical references. Data are presented as mean (standard deviation) or median (interquartile range), according to normality assumption.

Acquisition

Imaging type(s)	Structural MRI (standardize protocol)
Field strength	3.0 Tesla
Sequence & imaging parameters	1) variable flip angle 3D T2-weighted fluid-attenuated inversion recovery (FLAIR) turbo spin echo (repetition time [TR]= 4800 ms; echo time [TE]= 270 ms; inversion time [TI]= 1650 ms; matrix size = 256 × 256; field of view [FOV]= 256 × 256 mm2; echo train length [ETL]= 167; 192 contiguous 1 mm-thick sagittal slices);<br>2) 3D T1-weighted turbo field echo (TR= 7 ms; TE= 3.2 ms; TI= 1000 ms; flip angle= 8°; matrix size= 256 × 256; FOV= 256 × 256 mm2; 204 contiguous 1 mm-thick sagittal slices);<br>3) diffusion-weighted pulsed-gradient spin-echo single-shot echo-planar (TR= 5900 ms, TE= 78 ms; matrix size= 112 × 85; FOV= 240 × 233 mm2; 56 contiguous 2.3 mm-thick slices; number of excitations [NEX]= 1).
Area of acquisition	Whole brain scans.

Diffusion MRI ☒ Used ☐ Not used

Parameters: Diffusion-weighting (b-factor= 700/1000/2850 s/mm2) applied along 6/30/60 noncollinear directions and ten b=0 volumes distributed along the acquisition, plus three additional b=0 volumes with reversed polarity of gradients for distortion correction. No cardiac-gating.

Preprocessing

Preprocessing software	Lesions were segmented with a deep-learning algorithm (Valverde et al., Neuroimage 2017). FSL SIENAx software and FMRIB's Integrated Registration and Segmentation Tool (FIRST) pipeline (FMRIB, Oxford, UK) were used to measure brain volumes, while the FreeSurfer 6.0 software suite (http://surfer.nmr.mgh.harvard.edu/) was used for cortical thickness. Pre-processing of diffusion-weighted images included correction for off-resonance and eddy current induced distortions, as well as for movements using the Eddy tool within the FSL library. The diffusion tensor was estimated from the two lower shells by linear regression using FSL software (FMRIB, Oxford, UK).
Normalization	Brain volumes were normalized according to head-size by the scaling factor obtained from the SIENAX (V-scaling).
Normalization template	No normalization template was used. The mask of the subventricular zone was designed according to anatomical references (Cherubini et al., Neurosciences Letters 2010) in the Montreal Neurological Institute (MNI) space and registered on native 3D T1-weighted and diffusion-weighted images from each subject.
Noise and artifact removal	Only MR images with good quality (no artifact movements) were analyzed. Diffusion-weighted images were corrected for off-sesonance and eddy current induced distortions and movements using the Eddy tool in the FSL library.
Volume censoring	We should have answered no to this question. Indeed we confirm that we did not apply any volume censoring, since all the post-processing steps aimed at producing volume measures were checked. The quality review was applied to the Freesurfer and FSL-sienax pipeline and to the phases to obtain the subventricular zone. Furthermore the presence of outliers was assessed before the statistical analysis without receiving any alert that could justify the use of a censoring threshold.

Statistical modeling & inference

Model type and settings	Age-, sex- and phenotypes-adjusted linear models, partial correlations and stepwise multiple linear regressions were used to assess the damage of the subventricular zone in patients and to identify predictors of caudate volume and cognitive scores (Symbol digit modalities test [SDMT] z-scores).
Effect(s) tested	Associations between damage of the subventricular zone and 1) normalized caudate volume; 2) cognitive performance (SDMT z-score).

Specify type of analysis: ☐ Whole brain ☐ ROI-based ☒ Both

Anatomical location(s): A mask of the subventricular zone was designed in the Montreal Neurological Institute (MNI) space based on the anatomical references described by Cherubini et al., Neurosciences Letters 2010.

Statistic type for inference (See Eklund et al. 2016)	This does not apply to the study since this is not a voxel-wise analysis.
Correction	We did not include any correction at this stage (i.e., imaging analysis), since this was not voxel-wise. Head-size normalized brain volumes were afterwards included in a canonical statistical analysis (i.e., linear models, partial correlations and stepwise multiple linear regression), adjusted for the major covariates (i.e., age, sex, and phenotypes).

Models & analysis

n/a	Involved in the study
☒	☐ Functional and/or effective connectivity
☒	☐ Graph analysis
☐	☒ Multivariate modeling or predictive analysis

Multivariate modeling and predictive analysis	We used partial correlations and stepwise multiple linear regressions.<br>Dependent variables: 1) normalized caudate volume, 2) z-scores of the SDMT.<br>Independent variables: subventricular zone percentage lesion volume, fractional anisotropy in the normal appearing tissue of subventricular zone, intralesional fractional anisotropy in the subventricular zone, mean diffusivity in the normal appearing tissue of subventricular zone, intralesional mean diffusivity in the subventricular zone, normalized brain volume, normalized grey matter volume, normalized white matter volume, logarithm of T2-hyperintense lesion volume, normalized deep grey matter volume (excluding the caudate), normalized caudate volume (for the dependent variable=SDMT), white matter fractional anisotropy, white matter mean diffusivity, mean cortical thickness and cortical thickness of the frontal, parietal, temporal, occipital and cingulate lobes.
